# Anion Sensing through Redox‐Modulated Fluorescent Halogen Bonding and Hydrogen Bonding Hosts**

**DOI:** 10.1002/anie.202315959

**Published:** 2024-01-04

**Authors:** Andrew J. Taylor, Robert Hein, Sophie C. Patrick, Jason J. Davis, Paul D. Beer

**Affiliations:** ^1^ Department of Chemistry University of Oxford South Parks Road Oxford OX1 3QZ UK

**Keywords:** Anion Sensing, BODIPY, Halogen Bonding, Photoinduced Electron Transfer, Spectroelectrochemistry

## Abstract

Anion sensing via either optical or electrochemical readouts has separately received enormous attention, however, a judicious combination of the advantages of both modalities remains unexplored. Toward this goal, we herein disclose a series of novel, redox‐active, fluorescent, halogen bonding (XB) and hydrogen bonding (HB) BODIPY‐based anion sensors, wherein the introduction of a ferrocene motif induces remarkable changes in the fluorescence response. Extensive fluorescence anion titration, lifetime and electrochemical studies reveal anion binding‐induced emission modulation through intramolecular photoinduced electron transfer (PET), the magnitude of which is dependent on the nature of both the XB/HB donor and anion. Impressively, the XB sensor outperformed its HB congener in terms of anion binding strength and fluorescence switching magnitude, displaying significant fluorescence turn‐OFF upon anion binding. In contrast, redox‐inactive control receptors display a turn‐ON response, highlighting the pronounced impact of the introduction of the redox‐active ferrocene on the optical sensing performance. Additionally, the redox‐active ferrocene motif also serves as an electrochemical reporter group, enabling voltammetric anion sensing in competitive solvents. The combined advantages of both sensing modalities were further exploited in a novel, proof‐of‐principle, fluorescence spectroelectrochemical anion sensing approach, enabling simultaneous and sensitive read out of optical and electrochemical responses in multiple oxidation states and at very low receptor concentration.

## Graphical abstract

Fluorescence‐spectroelectrochemical anion sensing is introduced as a powerful method to enable anion detection via simultaneous emission and voltammetry readouts. The combined advantages of both approaches facilitate anion sensing at very low sensor concentrations and in a competitive solvent system using a redox‐active ferrocene‐containing BODIPY anion receptor, wherein photoinduced electron transfer (PET) modulates fluorescence emission.

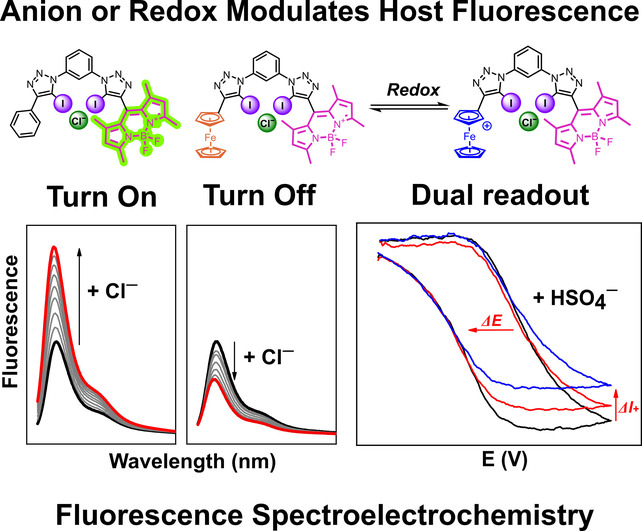

## Introduction

As ubiquitous constituents of nature, anions play vital roles in a myriad of technological, environmental and biological settings, necessitating their sensitive and selective detection. To this end, increasing efforts have been directed at the development of anion sensors, wherein the use of reversible non‐covalent interactions in supramolecular host–guest systems has emerged as a particularly promising approach for the construction of potent and reusable anion sensors.^[1]^ Sigma‐hole interactions, in particular halogen bonding (XB) and chalcogen bonding (ChB) have, as a result of their bonding strength and directionality, been recognised as powerful non‐covalent interactions to exploit in anion recognition.^[2]^

This has spurred on the development of a variety of derived XB^[3]^ or ChB^[4]^ anion sensors, whose properties are typically significantly enhanced, both in terms of selectivity and sensitivity, in comparison to sensors relying on more classical hydrogen bonding (HB) interactions.^[5]^ Particularly, redox‐active electrochemical voltammetric[^3c^, ^4a^, ^6^] and optical fluorescent^[7]^ sigma‐hole anion sensors have independently received significant attention and have enabled the development of increasingly advanced sensing systems, including those capable of repeat/continuous operation.^[8]^

Nevertheless, as has been previously noted, the response of XB fluorescent anion sensors to anion binding is often complicated,^[7d]^ which has hindered efforts to probe the mechanisms by which anion binding affects fluorescence emission. The need to obtain a fundamental understanding of these mechanisms is critical to designing anion sensors in which the ON or OFF direction of anion binding‐induced fluorescence changes can be strategically controlled. Photo‐induced electron transfer (PET) is one such mechanism of fluorescence modulation that has been deployed in the context of anion sensing.^[9]^ However, studies which seek to establish, in detail, the mechanisms by which anion binding modulates PET in anion sensors are scarce.

A combination of both optical and electrochemical approaches in anion sensing design remains underdeveloped. While various dual electrochemical/optical anion sensors have been reported,[^7e^, ^10^] where anion presence can be detected via either readout separately, a true *combination* of the advantages of both, specifically the oxidation‐induced anion binding enhancement of redox‐active receptors and the high sensitivity of fluorescence read‐outs, remains unexplored.

Herein, we address these challenges in the preparation and systematic study of XB and HB redox‐active, fluorescent anion receptors containing boron‐dipyrromethane (BODIPY) and ferrocene motifs.^[11]^ We not only demonstrate that the introduction of ferrocene into these fluorescent anion receptors has a profound impact on their optical anion sensing properties, but, through in‐depth photophysical and electrochemical studies, elucidate the mechanisms of fluorescence response to anion binding in unprecedented detail,^[12]^ including delineating the kinetic and thermodynamic contributions to PET modulation. Finally, we conduct preliminary investigations into fluorescence spectroelectrochemical anion sensing, a novel technique which allows the sensitive and *simultaneous* measurement of both optical and electrochemical responses upon anion binding—effectively permitting electrochemical anion sensing at nanomolar host concentrations.^[13]^

## Results and Discussion

**Figure 1:**
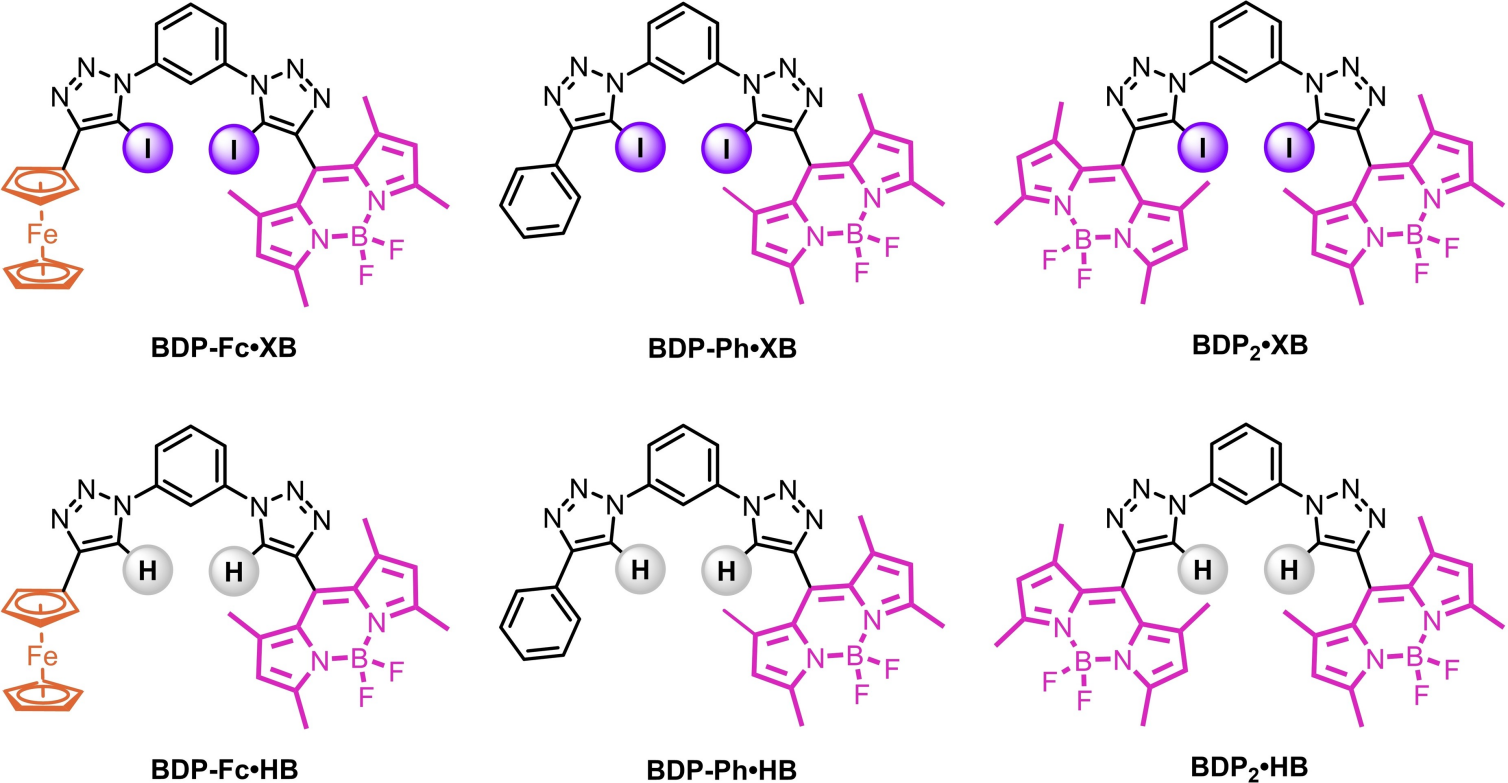
Structures of the title compounds **BDP‐Fc** ⋅ **XB/HB**, **BDP‐Ph** ⋅ **XB/HB**, and **BDP_2_** ⋅ **XB/HB**.

The target anion receptors feature the well‐established 1,3‐bis‐(iodo)triazole‐benzene anion binding scaffold, enabling strong XB or HB‐mediated anion recognition[^7e^, ^14^] and allowing facile modular synthesis of a family of six novel fluorescent XB and HB anion sensors, **BDP‐Fc** ⋅ **XB/HB**, **BDP‐Ph** ⋅ **XB/HB** and **BDP_2_** ⋅ **XB/HB** (Figure 1). In all cases, the BODIPY fluorophore was incorporated via a XB iodo‐triazole or HB proto‐triazole linkage in the BODIPY‐*meso* position. It was anticipated that the incorporation of the redox‐active ferrocene moiety in **BDP‐Fc** ⋅ **XB/HB** would lead to PET quenching of the BODIPY emission,^[15]^ the magnitude of which could conceivably be modulated by anion binding, due to both changes to the electronic environment as well as the receptor conformation. **BDP‐Ph** ⋅ **XB/HB** and **BDP_2_** ⋅ **XB/HB** served as control compounds, allowing comparisons with receptors without this mechanism of fluorescence modulation.

**Scheme 1:**
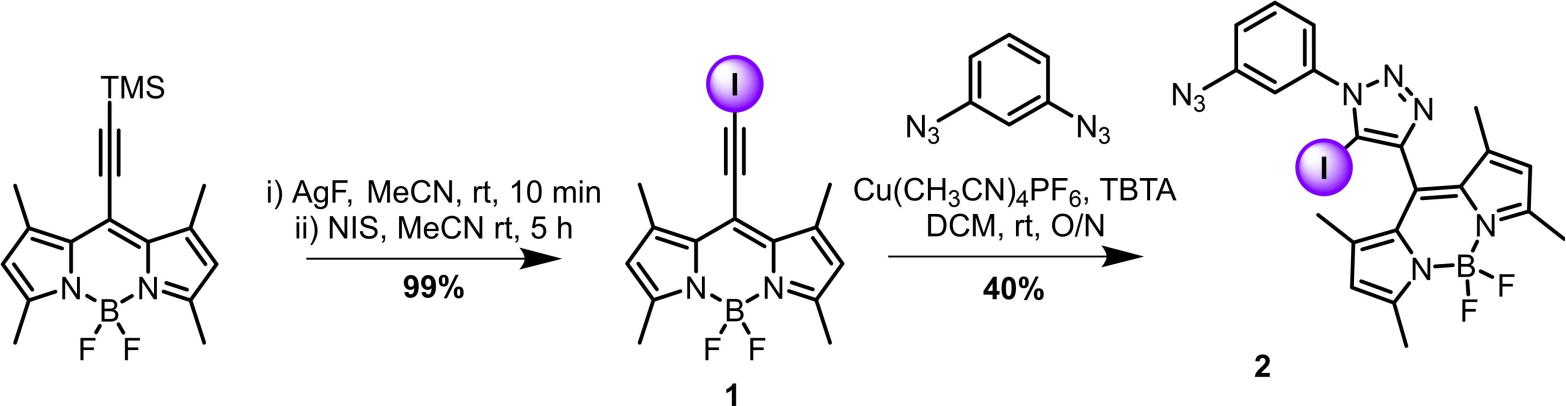
Synthesis of the mono‐click intermediate **2**.

**Scheme 2:**
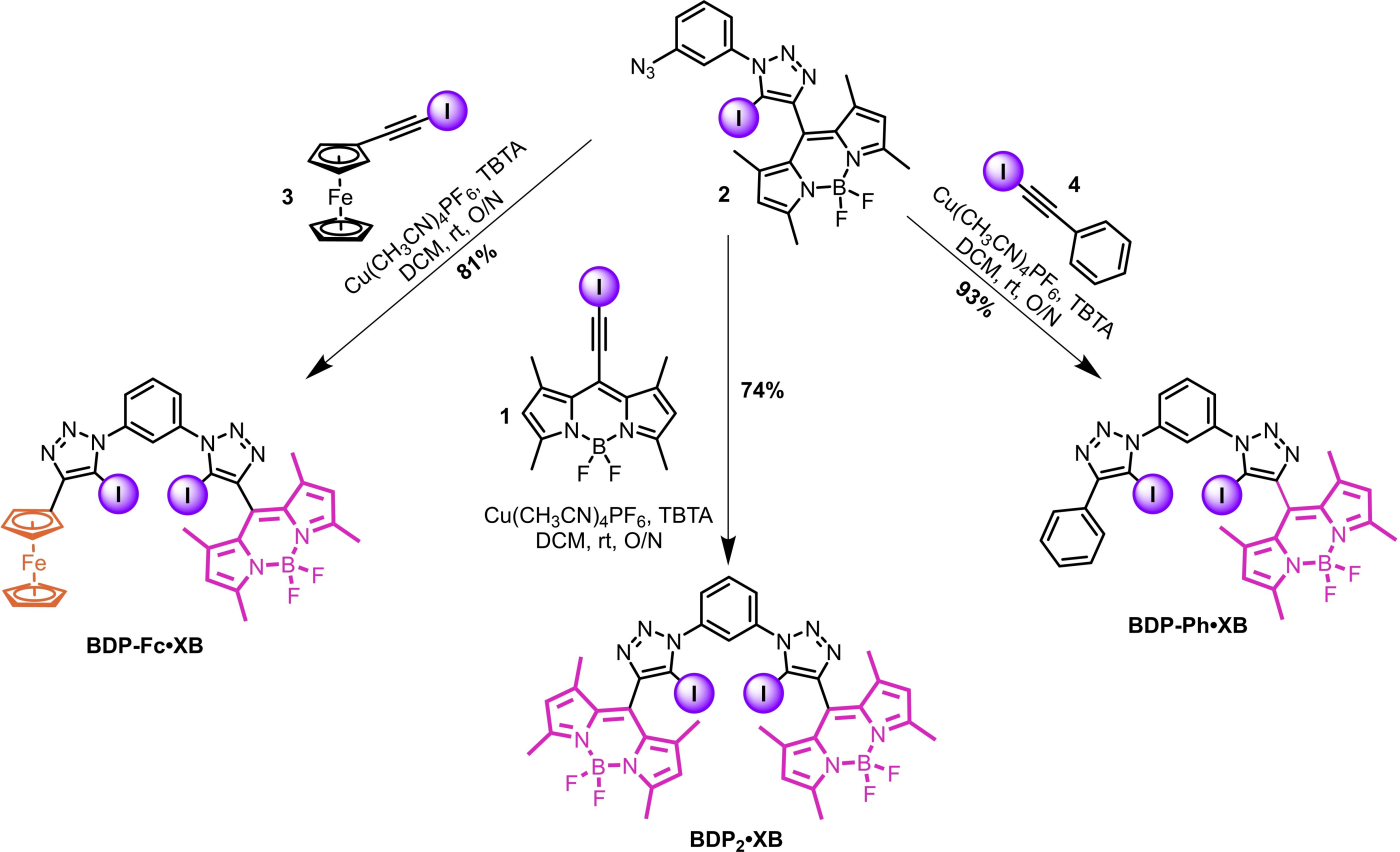
Synthesis of the title compounds **BDP‐Fc** ⋅ **XB**, **BDP_2_** ⋅ **XB**, and **BDP‐Ph** ⋅ **XB**.

Synthesis of the XB compounds (Schemes 1 and 2) began from TMS protected 8‐ethynyl‐BODIPY.^[16]^ TMS deprotection and iodination to 8‐(iodoethynyl)‐BODIPY **1** was achieved by treatment with N‐iodo‐succinimide (NIS) and AgF, in quantitative yield. This synthon was then subjected to typical CuAAC (‘click‘) conditions with 1,3‐diazido‐benzene,^[17]^ statistically producing the mono‐click product **2** in 40 % yield (Scheme 1).

Compound **2** was then re‐subjected to CuAAC conditions with either **1**, **3**, or **4** to afford **BDP_2_** ⋅ **XB**, **BDP‐Fc** ⋅ **XB**, and **BDP‐Ph** ⋅ **XB** in 74 %, 81 % and 93 % yields respectively (Scheme 2), whereby the requisite iodo‐alkyne CuAAC coupling partners **3** and **4** were both generated in quantitative yield by treatment of the parent proto‐alkyne with KOH and I_2_.

Synthesis of the analogous HB compounds (Figure 1) was achieved in high yields using a similar route, instead using the parent proto‐alkynes as the CuAAC coupling partners, as described in detail in the Supporting Information (Schemes S1–2). All novel compounds were characterised by ^1^H and ^13^C NMR spectroscopy as well as HR‐MS (see SI, Section S2).

**Figure 2:**
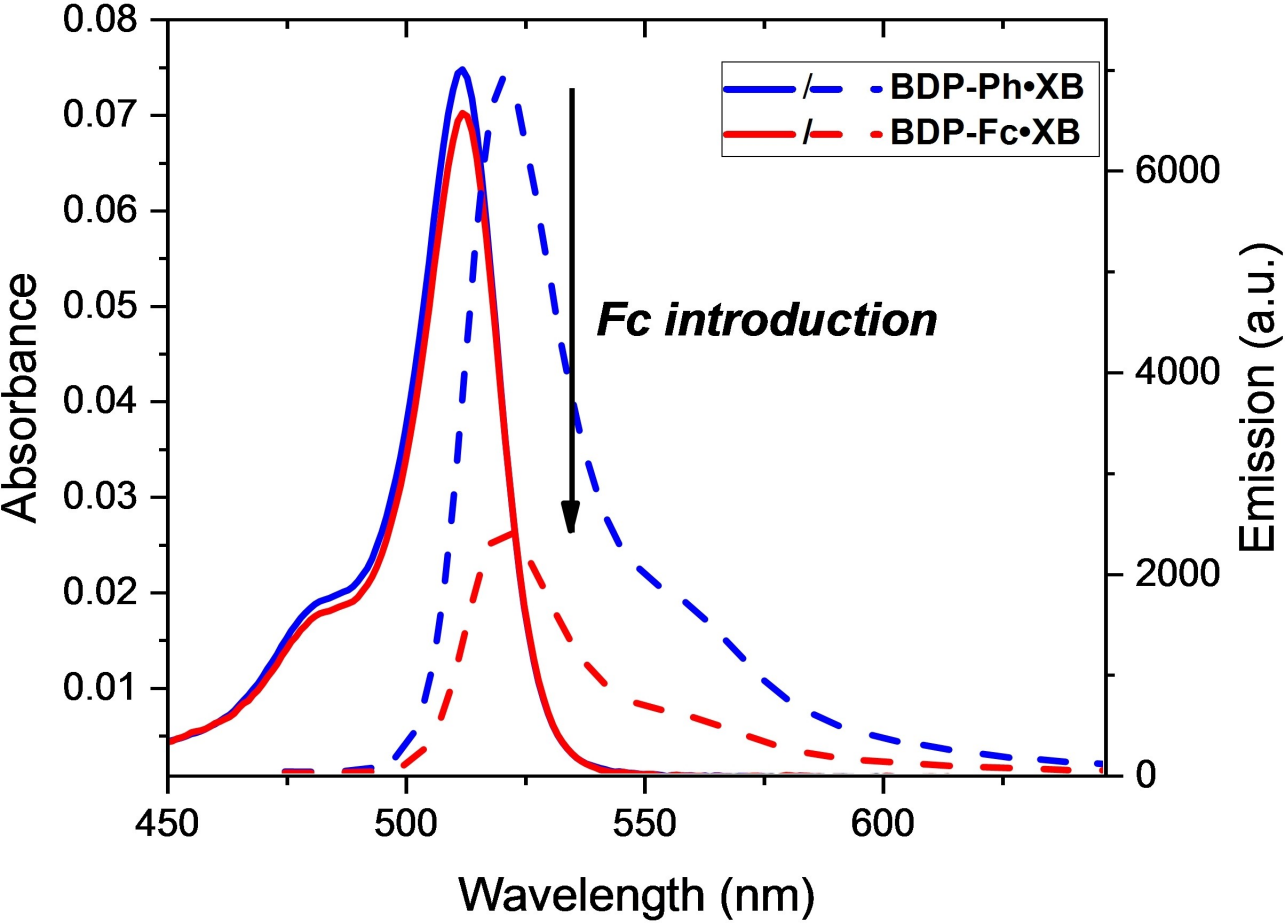
Absorbance (solid lines) and fluorescence emission intensity (dashed lines) of 1 μM **BDP‐Ph** ⋅ **XB** (blue) and **BDP‐Fc** ⋅ **XB** (red) in acetone.

As shown in Figure 2 and summarised in Table 1, in acetone all six receptors displayed typical BODIPY absorption and emission features, with absorbance and emission maxima around 510 nm and 520 nm, respectively. These maxima are identical within each set of XB and HB receptors, but the latter display small hypsochromic shifts of ≈2 nm in comparison to their XB counterparts.

**Table 1:** Photophysical properties of receptors in acetone.

	**BDP‐Ph**		**BDP_2_**		**BDP‐Fc**	
	XB	HB	XB	HB	XB	HB
**λ_max, abs_ (nm)**	512	509	512	509	512	510
**ϵ (M^−1^cm^−1^)**	74700	89500	140500	192000	76900	92600
**λ_max, em_ (nm)**	521	519	521	520	521	519
**Φ (%)^[a]^**	17	4	14	4	6	3

[a] Measured against fluorescein as standard in 0.1 M NaOH.

These observations confirm that the Fc/Ph/BDP substituents have no significant effect on the wavelength of the absorbance and emission features of the receptors, and, similarly, that the difference in polarity of the XB/HB (iodo)triazole motif does not strongly affect the appended BODIPY reporter group. The molar absorption coefficient ϵ is somewhat larger for all HB congeners (≈90000 M^−1^ cm^−1^ per BODIPY) than for the XB receptors (≈75000 M^−1^ cm^−1^ per BODIPY). More importantly, the fluorescence quantum yields (Φ) display noteworthy differences between the receptors, with generally identical quantum yields for the BDP_2_ or BDP‐Ph congeners of the same XB/HB donor motif. For example, Φ was identical, within error, for the XB receptors **BDP‐Ph** ⋅ **XB** (Φ=0.17) and **BDP_2_** ⋅ **XB** (Φ=0.14), again indicative of minimal interactions between the BDP units in the BDP_2_ receptors. Interestingly, the same trend was also observed for the corresponding HB receptors, albeit with much lower quantum yields of Φ=0.04 for both **BDP‐Ph** ⋅ **HB** and **BDP_2_** ⋅ **HB**. These ≈4‐fold lower quantum yields for the HB receptors are somewhat surprising as multiple literature reports demonstrate the opposite trend, wherein heavy‐atom effects are often invoked to rationalise lower quantum yields for XB iodotriazole‐containing receptors.^[5]^

In the receptors developed herein, we propose that the bulky iodine substituent on the triazole motifs significantly restricts rotation around the *meso*‐BODIPY bond, thereby reducing non‐radiative decay pathways and boosting fluorescence, as observed in other *meso*‐BODIPY‐functionalised systems.^[11b]^ In contrast, the HB receptors display less inhibited rotation about the *meso‐*BODIPY bond, and hence lower fluorescence quantum yields. To further probe this, the fluorescence emission of **BDP‐Ph** ⋅ **HB** was investigated as a function of solvent viscosity. As shown in Figure S29, addition of increasing amounts of glycerol to the receptor in MeOH induced marked increases in emission intensity, the magnitude of which correlated well with the solvent viscosity (Figure S30). These observations are in agreement with recent studies on a structurally related *meso*‐functionalised BODIPY and confirm that this rotational decay pathway is significant.^[18]^ The molecular rotor nature of the receptors might lead to these compounds also finding application as (ion‐responsive) viscosity probes.^[19]^

The enhanced quantum yields of **BDP‐Ph** ⋅ **XB** and **BDP_2_** ⋅ **XB** in comparison to their HB congeners can thus be attributed to steric inhibition of rotation of the *meso*‐BODIPY bond due to the bulky iodo‐triazole motif, thereby suppressing non‐radiative decay pathways. Importantly, the magnitude of this effect is large enough to overcome any emission quenching arising from heavy atom effects in the XB systems.

While the aforementioned substitution of a BODIPY group for a phenyl group has no significant effect on the quantum yields of the receptors, the introduction of the redox‐active ferrocene (Fc) motif does importantly alter the fluorescence properties of both **BDP‐Fc** ⋅ **XB** and **BDP‐Fc** ⋅ **HB**, which exhibit diminished quantum yields of Φ=0.06 and Φ=0.03, respectively. This significant fluorescence quenching is consistent with PET from the ferrocene to the (photo‐excited) BODIPY, as observed in previous studies[^11a^, ^15^, ^20^] and as described in more detail below.

**Figure 3:**
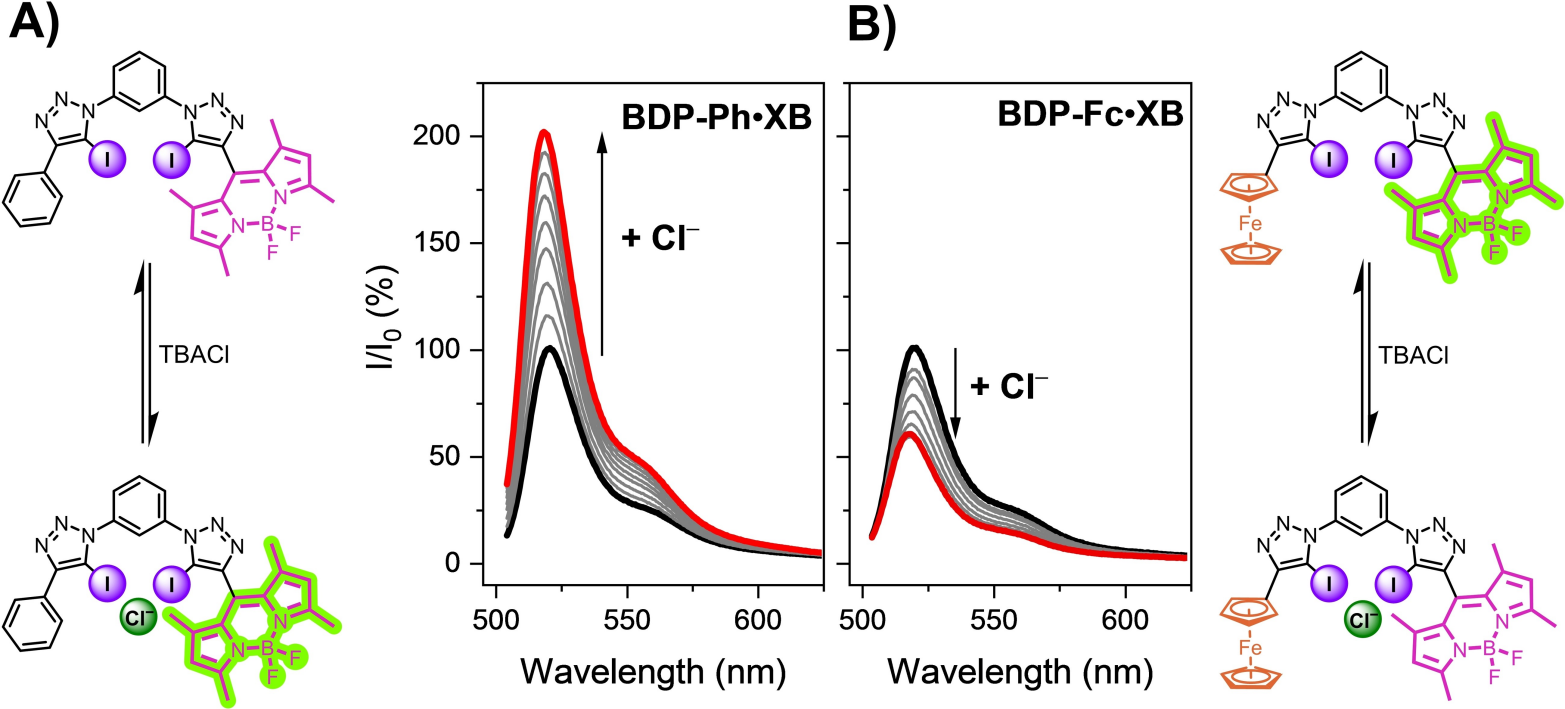
Normalised fluorescence emission response of 1 μM A) **BDP‐Ph** ⋅ **XB** and B) **BDP‐Fc** ⋅ **XB** upon addition of increasing concentrations of Cl^−^ (up to ca. 2.8 mM) in acetone.

The anion sensing properties of all receptors were probed in acetone solution by addition of aliquots of tetrabutylammonium halide salts (TBAX, X=Cl, Br, I). As representatively shown in Figure 3A, chloride and bromide anions induced large fluorescence turn‐ON of up to 100 % for both **BDP_2_** ⋅ **XB** and **BDP‐Ph** ⋅ **XB**, with no significant changes in either absorbance or emission maxima. Iodide induced more moderate fluorescence turn‐ON, which was somewhat larger for **BDP_2_** ⋅ **XB** (27 %) than **BDP‐Ph** ⋅ **XB** (7 %).

We hypothesise that this turn‐ON response arises from anion binding‐induced receptor rigidification, specifically restriction of *meso*‐BODIPY bond rotation, as discussed in more detail below.

**Figure 4:**
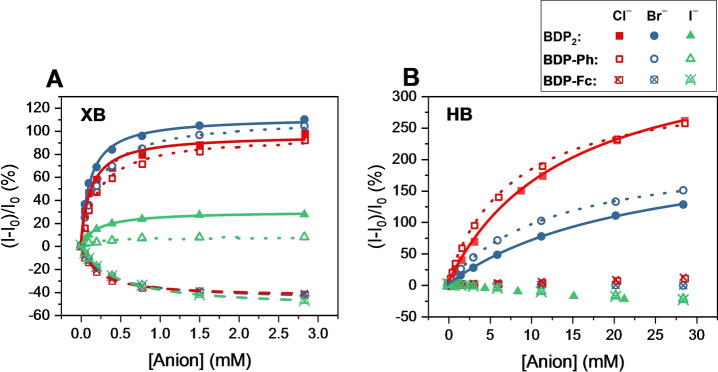
Relative fluorescence response of 1 μM **BDP_2_** (filled symbols), **BDP‐Ph** (empty symbols) and **BDP‐Fc** (empty, crossed symbols) receptors in acetone upon addition of halide anions. A) XB receptors, B) HB receptors. Lines represent fits according to a 1 : 1 host–guest stoichiometric binding model. Note the different concentration ranges for both graphs.

Interestingly, the anion sensing behaviour of the redox‐active **BDP‐Fc** ⋅ **XB** differed markedly from the other two receptors, with significant fluorescence *quenching* of ≈45 % for all three halide anions (Figure 3B and 4). The simple substitution of a Ph (or BDP) substituent for ferrocene thus induces a complete fluorescence response reversal towards all anions from turn‐ON to turn‐OFF.

It is important to note that this reversal in sensing behaviour must arise from altered fluorescence response pathways and not differing anion binding preferences. Specifically, fitting of the fluorescence response isotherms to a 1 : 1 host–guest stoichiometric binding model afforded anion binding constants, which were in all cases identical, within error, for **BDP‐Fc** ⋅ **XB** and **BDP‐Ph** ⋅ **XB** (Table 2 and Figure 4A), highlighting that ferrocene substitution does not affect the strength of anion binding. For example, both receptors displayed a modest binding preference for chloride, the most charge‐dense anion tested, with K≈4400 M^−1^ and relatively weaker binding for bromide (≈3900 M^−1^) and iodide (≈2200 M^−1^). Due to the electron‐withdrawing effect of the BODIPY motif, the anion binding strength of **BDP_2_** ⋅ **XB** was 2‐fold augmented for all halide anions, with a preference for Br^−^ (K=8600 M^−1^).

**Table 2:** Halide association constants K (M^−1^)^[a]^ of all receptors.

	**BDP_2_**		**BDP‐Ph**		**BDP‐Fc**	
	XB	HB	XB	HB	XB	HB
**Cl−**	7860	70	4480	126	4390	n/a
**Br−**	8600	28	4030	78	3770	n/a
**I−**	4550	n/a	2340	n/a	2040	n/a

[a] Determined in acetone at 298 K by global fitting of fluorescence isotherms to 1 : 1 host–guest stoichiometric binding model. All errors are <6 %. n/a: not applicable due to small perturbations.

Importantly, the anion binding induced photo‐response behaviour of all HB receptors differed significantly from the above‐discussed turn‐ON/OFF response switching of the XB receptor analogues. In analogy to their XB counterparts, both **BDP_2_** ⋅ **HB** and **BDP‐Ph** ⋅ **HB** displayed identical and large fluorescence enhancements upon binding of chloride (turn‐ON of >250 %) and bromide (turn‐ON of >125 %), albeit only at significantly higher anion concentrations, a reflection of their much weaker anion binding affinities (K≈30–130 M^−1^), in agreement with previous studies highlighting the increased anion binding potency of XB receptors over HB receptor analogues (Figures 4A and 4B, respectively).[^2a^, ^2b^, ^5^] In contrast, iodide induced only minor fluorescence decreases (up to −25 %), most likely as a result of heavy‐atom, non‐specific quenching.

Unexpectedly, **BDP‐Fc** ⋅ **HB** displayed virtually no fluorescence response towards all halides. In analogy to the **BDP‐Ph** ⋅ **XB/BDP‐Fc** ⋅ **XB** system, for which near identical anion binding strength was determined, we surmise that the anion binding affinity of **BDP‐Ph** ⋅ **HB** and **BDP‐Fc** ⋅ **HB** is at least in a similar range. This was confirmed by ^1^H NMR titration of **BDP‐Fc** ⋅ **HB** with bromide in acetone‐*d_6_*, revealing an anion binding constant of K=152 M^−1^ (Figures S31–32) which is very similar to that of **BDP‐Ph** ⋅ **HB** (K=78 M^−1^) as determined by fluorescence titrations (Table 2). Hence, the negligible halide anion responsiveness of **BDP‐Fc** ⋅ **HB** does not arise from lack of binding, but most likely from simultaneous, and identical, opposing turn‐ON and turn‐OFF transducing mechanisms through different pathways which are discussed in more detail below.

To gain further insights into the unexpected and contrasting anion binding response behaviour of the receptors, fluorescence lifetime and electrochemical studies were carried out. The former revealed mono‐exponential lifetime decays in the ps‐ns range. As summarised in Table 3, the addition of bromide as a model anion to all receptors induced significant changes in the lifetimes of the receptors, with notable lifetime increases for the **BDP‐Ph** ⋅ **XB/HB** and **BDP_2_** ⋅ **XB/HB** receptors and a significant lifetime decrease for **BDP‐Fc** ⋅ **XB**. These observations confirm that fluorescence modulation by anion binding occurs through a dynamic quenching mechanism, which is further supported by no observed changes in receptor absorbance upon anion binding.

**Table 3:** Fluorescence lifetimes^[a]^ (ns) of all receptors in the presence and absence of excess TBABr^[b]^.

	**BDP_2_**		**BDP‐Ph**		**BDP‐Fc**	
	XB	HB	XB	HB	XB	HB
**Free Host**	1.14	0.28	1.28	0.25	0.41	0.17
**Host ⋅ Br^−^**	2.32	0.97	2.49	0.92	0.24	0.18

[a] Determined in acetone at 298 K. Errors <5%. [b] 3 mM for XB and 30 mM for HB.

It is noteworthy that for the XB receptors the changes in fluorescence lifetime (*τ_f_*) upon anion binding are strongly correlated with the magnitude of the fluorescence intensity changes observed over the course of the anion titrations.^[21]^ For example, **BDP‐Fc** ⋅ **XB** displays a 43 % quenching in fluorescence intensity in the presence of excess bromide, and a decrease in lifetime of 41 %. Conversely, **BDP_2_** ⋅ **XB** shows a 109 % increase in fluorescence intensity at the end of the titration, and an increase in fluorescence lifetime of 103 %.

Therefore, for a given XB receptor, *τ_f_* and fluorescence intensity appear to be modulated by a similar factor upon bromide binding, which suggests that anion binding does not have a significant effect on the rate of emission from the S_1_ state of the BODIPY fluorophore, but instead significantly affects the rate of other, non‐radiative decay pathways that are available from the S_1_ state (*k′*) (see Supporting Information Section S5). In the case of **BDP‐Ph** ⋅ **XB** and **BDP_2_** ⋅ **XB**, this can be ascribed to anion binding‐induced rigidification of the structure decreasing the rate of internal conversion, decreasing *k′* and hence increasing both *τ_f_* and fluorescence intensity. In analogy to prior discussions on the influence of the steric bulk in the vicinity of the *meso*‐BODIPY bond and the resulting restriction of rotation (see above), we surmise that anion binding induces a further “locking” of rotation, thereby enhancing quantum yields. The same rigidification mechanism is presumably responsible for the increase in emission intensity observed in **BDP‐Ph** ⋅ **HB** and **BDP_2_** ⋅ **HB**, which both also show an increase in fluorescence lifetime.

**Figure 5:**
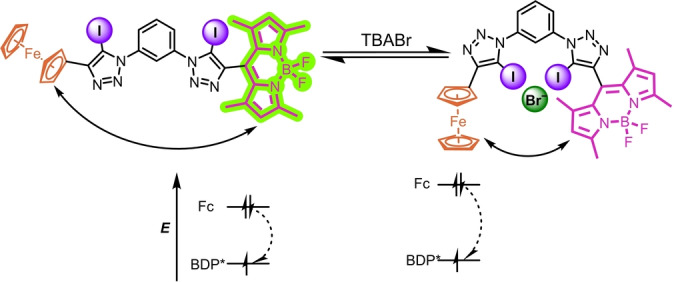
Schematic representation showing the kinetic and thermodynamic enhancement of PET in **BDP‐Fc** ⋅ **XB** upon anion binding, via receptor conformational changes and an increase in the electron density at the ferrocene donor, respectively.

In the case of the redox‐active receptors **BDP‐Fc** ⋅ **XB** and **BDP‐Fc** ⋅ **HB**, an *additional*, opposing effect overrides this rigidification effect. For **BDP‐Fc** ⋅ **XB** an overall increase in *k′* is observed, thus decreasing *τ_f_* and fluorescence intensity.^[21]^ We hypothesise that this is a result of an enhancement of PET, which may arise, at least in part, from conformational changes that enhance the kinetics of PET by bringing the Fc quencher and the BODIPY into closer proximity upon convergent anion binding by both XB donor iodo‐triazole motifs (Figure 5). This is supported by comparisons of the rate of electron transfer k_ET_ from the ferrocene moiety to the BODIPY fluorophore between the free **BDP‐Fc** ⋅ **XB/HB** host and in the presence of bound bromide. Specifically, k_ET_ can be calculated from the fluorescence lifetimes by comparison of the **BDP‐Fc** hosts to the “reference” **BDP‐Ph** via:^[22]^

(1) $$k_{ET}=\frac{1}{\tau_{BDP-Fc}}-\frac{1}{\tau_{BDP-Ph}}\;$$

For both **BDP‐Fc** ⋅ **XB** and **BDP‐Fc** ⋅ **HB** the initial electron transfer rate in the unbound state is largely identical with $k_{ET}=1.66\;\times 10^{9}\;s^{-1}$ and $1.88\;\times 10^{9}\;s^{-1}$ , respectively. In both cases the $k_{ET}$ increases significantly upon anion binding to $3.77\;\times 10^{9}\;s^{-1}$ and $4.47\;\times 10^{9}\;s^{-1}$ , respectively, indicative of kinetically enhanced PET upon anion binding (Table 4). In addition, the PET efficiency $\theta_{ET}$ can also be considered via:^[22]^

(2) $$\theta_{ET}=1-\frac{\tau_{BDP-Fc}}{\tau_{BDP-Ph}}$$

**Table 4:** Rate of electron transfer (k_ET_), PET efficiency (θ_ET_) and Gibbs free energy of electron transfer (ΔG_ET_) of **BDP‐Fc** ⋅ **XB/HB** in the absence and presence of excess TBABr^[a]^ obtained from Equations1‐3.

	**k_ET_** ^**[b]**^ **(s^−1^)**		**θ_ET_** ^**[b]**^		**ΔG_ET_** ^**[c]**^ **(eV)**	
	XB	HB	XB	HB	XB	HB
**BDP‐Fc**	1.66×10^9^	1.88×10^9^	0.68	0.68	−0.903	−0.935
**BDP‐Fc** ⋅ **Br^−^**	3.77×10^9^	4.47×10^9^	0.90	0.80	−0.933	n.d.

[a] 3 mM for XB and 30 mM for HB. [b] In acetone. [c] In acetonitrile in the presence of 0 or up to 50 mM TBABr. n.d.: Not determined.

As collated in Table 4, this PET efficiency increases for both receptors in the presence of the bromide guest.

Additionally, anion binding might also render PET more thermodynamically feasible. To quantify the PET process from a thermodynamic point of view, the Gibbs free energy of electron transfer $\Delta G_{ET}$ was determined via:[^15c^, ^22^, ^23^]

(3) $$\Delta G_{ET}=e\left[E_{Ox,\;Don}-E_{Red,\;Acc}\right]-E_{00}-\Delta G_{S}\;$$

Where, E_Ox_ and E_Red_ are the half‐wave potentials of the donor (Fc) and acceptor (BODIPY), respectively, $E_{00}\;$ the photoexcitation energy for the BODIPY and $\Delta G_{S}$ the Coulombic energy of the charge separated state, which is negligible herein, as discussed in more detail in the Supporting Information (Section S7). The photoexcitation energy $E_{00}\;$ of the BODIPY motif was obtained from the midpoint between absorption and emission maxima and was found to be almost identical for **BDP‐Fc** ⋅ **XB** and **BDP‐Fc** ⋅ **HB** at 2.40 eV and 2.41 eV, respectively (see Supporting Information Section S7).^[24]^

The oxidation and reduction potentials of **BDP‐Fc** ⋅ **XB/HB** were obtained via voltammetry in anhydrous, degassed acetonitrile in the presence of 100 mM TBAPF_6_. Both the ferrocene/ferrocenium as well as the reductive BODIPY couple (BODIPY/BODIPY^−^) of **BDP‐Fc** ⋅ **XB/HB** display good electrochemical reversibility, while the oxidation of BODIPY is irreversible in both cases (Figure S33). As summarised in Table 5, the BODIPY reduction occurs at identical potentials for both receptors, while Fc oxidation (and BODIPY oxidation), is less facile in the XB system. This is indicative of an enhanced electron‐withdrawing effect of the iodo‐triazole group and is in good agreement with related systems.[^3c^, ^6a^, ^25^]

**Table 5:** Half‐wave potentials (*E*_1/2_) in V^[a]^ vs. Fc/Fc^+^ of all electrochemical couples **of BDP‐Fc** ⋅ **XB/HB** in anhydrous, degassed ACN obtained by square‐wave voltammetry (SWV). The reversibility of the oxidative BDP couple is poor; the stated potentials correspond to the anodic peak potential.

	**BDP/BDP^−^**	**Fc/Fc^+^**	**BDP/BDP^+^**
**BDP‐Fc** ⋅ **XB**	−1.400	0.097	0.843
**BDP‐Fc** ⋅ **HB**	−1.402	0.073	0.803

[a] Estimated error ±2 mV.

From these potentials and $E_{00}$ , the Gibbs free energy of electron transfer ($\Delta G_{ET}$ ) was calculated as −0.903 eV and −0.935 eV for **BDP‐Fc** ⋅ **XB** and **BDP‐Fc** ⋅ **HB**, respectively, confirming that in both cases PET is thermodynamically viable (see SI, Section S7). We further assessed the *change* in $\Delta G_{ET}$ upon anion binding. To this end voltammetric anion binding titrations were carried out for **BDP‐Fc** ⋅ **XB** with bromide in acetonitrile (see Supporting Information Figures S34–35 and Section S6 for further details and discussions). Even in this more polar solvent system, notable cathodic voltammetric shifts of both the Fc/Fc^+^ and BDP/BDP^−^ were observed, whereby the latter were ≈30 mV smaller (Figure S35). Thus, even though both couples are perturbed, the absolute value of ${e[E}_{Ox,\;Don}-E_{Red,\;Acc}]$ overall becomes less positive, leading to a more favorable $\Delta G_{ET}$ of −0.933 eV for **BDP‐Fc** ⋅ **XB** in the presence of bromide (Table 4 and Figure 5).

Together, these results suggest that anion binding enhances PET quenching of **BDP‐Fc** ⋅ **XB**/**HB** both kinetically, presumably due to conformational changes, and thermodynamically (Figure 5). However, only in the case of **BDP‐Fc** ⋅ **XB** does this lead to an overall turn‐OFF fluorescence response towards the halide anions, while **BDP‐Fc** ⋅ **HB** is largely unresponsive (Figure 4B). One possibility is that this observation arises from an *inherently* higher anion binding‐induced fluorescence turn‐ON through receptor rigidification in the HB system, such that the PET enhancement is not able to *over*‐compensate this inherent turn‐ON response (as is the case for **BDP‐Fc** ⋅ **XB**). This seems likely, given the larger fluorescence turn‐ON of both **BDP‐Ph** ⋅ **HB** and **BDP_2_** ⋅ **HB** in comparison to their XB congeners (Figure 4).

Having established that the ferrocene moiety has a significant effect on BODIPY emission in its native, neutral state, attention turned to investigating the effect of ferrocene oxidation on the fluorescence properties of **BDP‐Fc/Fc^+^**. This was initially probed by chemical oxidation of **BDP‐Fc** ⋅ **XB** with Fe(ClO_4_)_3_ as oxidant in acetone. Interestingly, this oxidation to the ferrocenium state (**BDP‐Fc^+^** ⋅ **XB**) did not induce any significant changes in absorbance nor the fluorescence emission maximum, but led to further fluorescence quenching by a factor of ≈2 (Φ≈0.03, in acetone). Oxidation of Fc‐BODIPY systems is typically associated with recovery of fluorescence (by turn‐OFF of PET),[^15^, ^20^] however ferrocenium has also been reported as a (better) fluorescence quencher, such that oxidation sometimes induces the opposite effect.^[26]^ It has, for example, been suggested that ferrocenium can quench fluorescence via oxidative PET.^[26a]^

**Figure 6:**
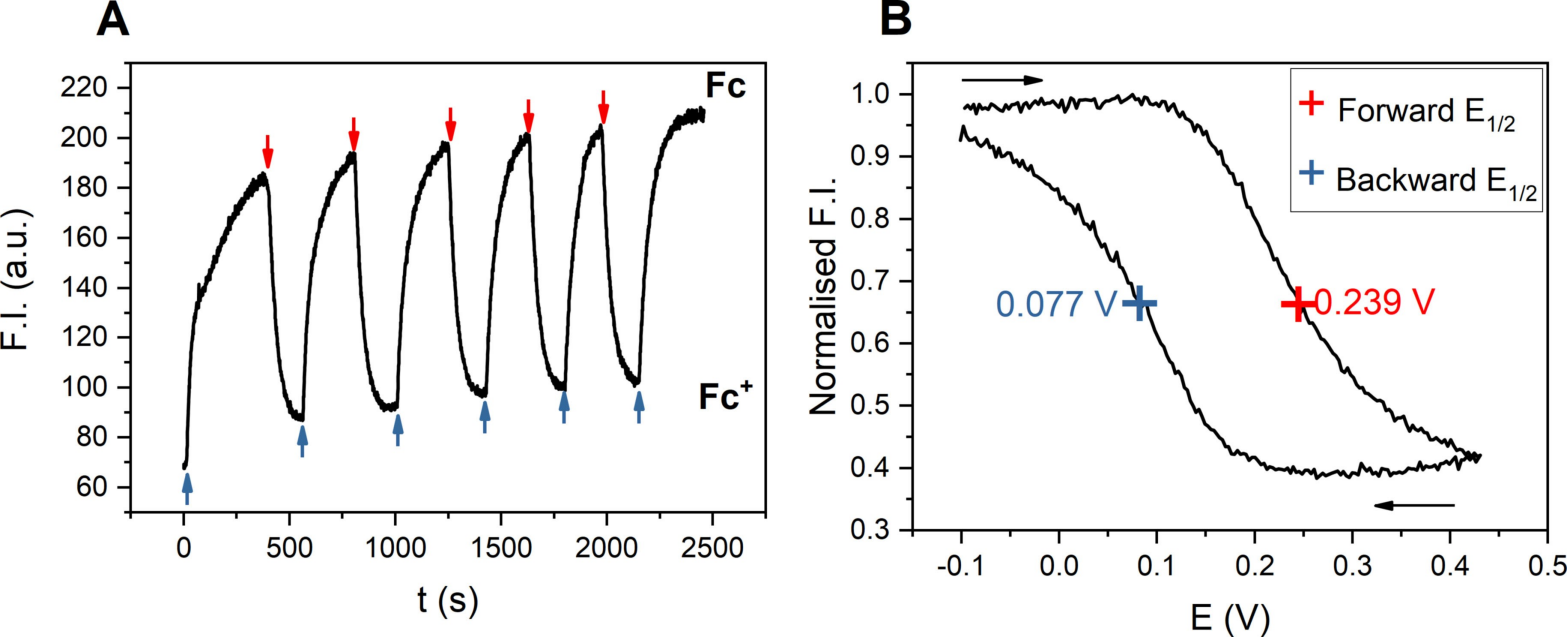
Fluorescence spectroelectrochemical experiments. The emission was monitored at λ_max_ in all cases. All potentials are with respect to Ag|AgNO_3_. A) Fluorescence of 100 nM **BDP‐Fc** ⋅ **XB** in ACN/1 % H_2_O, 100 mM TBAClO_4_, 100 μM HClO_4_ under alternating reductive (blue arrows) and oxidative potentials (red arrows). Note under these conditions the receptor is initially present in its oxidised state. B) Normalised fluorescence emission of 500 nM **BDP‐Fc** ⋅ **HB** in ACN, 100 mM TBAClO_4_, 200 μM HClO_4_ during one CV scan between −0.1 and 0.45 V at 0.75 mV/s. The black arrows indicate the scan direction.

Having established that **BDP‐Fc** fluorescence is highly sensitive to not only the presence of the (neutral) ferrocene but also to its oxidation state, attention turned to exploiting the BODIPY fluorescence as a sensitive readout of the receptor redox state. To this end, the fluorescence spectroelectrochemical^[27]^ properties of 100 nM **BDP‐Fc** ⋅ **XB** were first probed in ACN/1 % H_2_O, 100 mM TBAClO_4_, 100 μM HClO_4_. These solvent/electrolyte conditions were chosen to enhance the chemical stability of the generated ferrocenium receptor state upon multiple cycles of bulk electrolysis and to circumvent formation of unwanted electrolysis by‐products. As shown in Figure 6A, repeat bulk electrolysis of a rapidly stirred solution of 100 nM **BDP‐Fc** ⋅ **XB** in a standard fluorescence cuvette induced well‐defined, highly reversible fluorescence switching upon application of alternating oxidative (lower fluorescence, **BDP‐Fc^+^**) and reductive potentials (higher fluorescence, **BDP‐Fc**). These observations confirm a reversible redox‐dependent fluorescence modulation of **BDP‐Fc** ⋅ **XB** with a two‐fold emission intensity switching magnitude. Importantly, the high sensitivity of the fluorescence readout enables use of very low probe concentrations (<1 μM), thereby enabling fast bulk electrolysis.

Further experiments exploited this reversible fluorescence modulation to probe the electrochemical properties of the ferrocene‐containing receptors via fluorescence spectroscopy. By slowly, cyclically varying the potential applied to the cell and monitoring the emission of 500 nM **BDP‐Fc** ⋅ **HB** in ACN, 100 mM TBAClO_4_, 200 μM HClO_4_, a ‘fluorescence CV’ could be obtained (Figure 6B), which is a direct optical readout of the Nernstian redox distribution imposed on the bulk cell. Due to a lag between applying the potential and the establishment of bulk equilibrium, presumably due to slow mass transport in the crowded cuvette, the forward and backward scans do not overlap.

However, the half‐wave potential of the receptor can still be determined from this experiment by taking the mean of the two forward and backward potentials, affording *E*_1/2_=0.158 V (vs. Ag|AgNO_3_), which is identical to the *E*_1/2_ of 0.16 V, obtained by traditional electrochemical means under near identical conditions (Figure S36). Of particular note is this fluorescence readout sensitively reports on the redox state distribution of the probe at much lower host concentrations (here 500 nM) than are attainable by standard voltammetric techniques (typically >100 μM host concentration).

Encouraged by these results, we sought to simultaneously measure anion binding‐induced optical and electrochemical responses in this manner. The latter are typically determined by standard voltammetric methods, wherein in situ electrochemical oxidation of redox‐active ferrocenyl XB or HB receptors is associated with both cathodic voltammetric shifts of the ferrocene couple as well as anion binding enhancements in the oxidised receptor state, enabling sensing in more polar, aqueous solvent systems.[^1a^, ^3c^, ^6a^] This behaviour was expectedly also observed for **BDP‐Fc** ⋅ **XB/HB**, which display well‐defined cathodic voltammetric perturbations in both acetone and even in ACN/1 % H_2_O in standard electrochemical anion sensing studies, as shown and discussed in more detail in the Supporting Information (Section S8, Figures S37–38 and Table S1). The largest cathodic shift (up to Δ*E*=−37 mV in the presence of 50 mM anion) in the aqueous organic solvent system was observed for bisulfate recognition with **BDP‐Fc** ⋅ **HB**, which was thus chosen as a model system for further fluorescence spectroelectrochemical studies. Additionally, this anion is not redox active in the relevant potential window, thereby preventing the generation of potentially interfering electrolysis by‐products under repeat, exhaustive bulk electrolysis.

**Figure 7:**
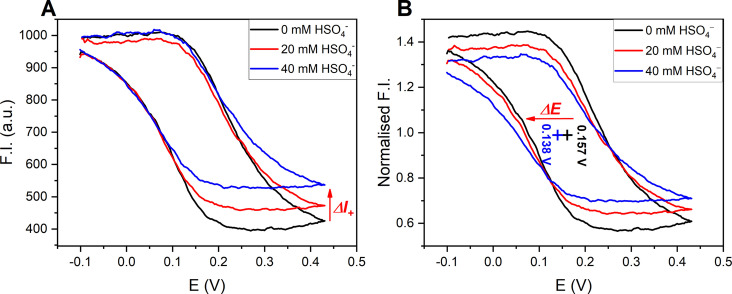
‘Fluorescence cyclic voltammograms of 500 nM **BDP‐Fc** ⋅ **HB** in ACN, 100 mM TBAClO_4_, 200 μM HClO_4_ in the presence of various concentrations of TBAHSO_4_. The emission was monitored at λ_max_ in all cases. All potentials are with respect to Ag|AgNO_3_. A) Absolute fluorescence intensity. The red arrow indicates the change in the fluorescence emission in the oxidised receptor state. B) Normalised fluorescence intensity. Each set of data is normalised by the fluorescence intensity at *E*_1/2_ to highlight the cathodic potential shift (pluses and red arrow).^[28]^

Gratifyingly, addition of increasing concentrations of this anion to **BDP‐Fc** ⋅ **HB** induced, in successive “fluorescence CV” scans, notable cathodic shifts of −11 and −19 mV in the presence of 20 and 40 mM HSO_4_^−^, respectively (Figure 7B). While somewhat smaller than the shifts observed under similar conditions by standard voltammetry (Figure S38), these results nevertheless confirm that this novel detection approach enables *simultaneous* readout of voltammetric *and* fluorescence response patterns. The latter is easily obtained by comparison of the absolute fluorescence intensity at the vertices of the CV scan, wherein the observed change at the anodic vertex corresponds to the fluorescence response of the cationic **BDP‐Fc^+^** ⋅ **HB** (turn‐ON, Figure 7A).

There is no change in fluorescence emission at the cathodic vertex, as the neutral receptor is not expected to bind HSO_4_^−^ in the competitive ACN solvent system. Indeed, bisulfate binding to the related **BDP‐Ph** ⋅ **HB** is already weak in acetone (*K*=43 M^−1^, see Figure S39) and is negligible in the (spectro)electrochemical solvent system (see Figures S40–41 and associated discussions).

This also highlights one of the crucial advantages of this novel methodology: electrochemical oxidation is associated with anion binding enhancement that enables sensing in the polar solvent medium and in situ generation of a novel, cationic receptor state, neither of which can be achieved by fluorescence sensing alone, while the latter enables spectroelectrochemical sensing at much lower receptor concentrations than what is achievable by voltammetric means alone (herein 500‐fold lower). The spectroelectrochemical “fluorescence CV” readout thus not only combines the advantages of fluorescence (high sensitivity) and electrochemical techniques (redox‐induced binding modulation) in one experiment, but also enables facile simultaneous readout of *three different* sensing parameters (ΔE, ΔI_Neutral_ and ΔI_Oxidised_), which can otherwise only be obtained separately or are difficult to access at all (ΔI_Oxidised_). Current efforts in our laboratory are focused on a further exploration of this approach.

## Conclusion

Through the synthesis and extensive photophysical and electrochemical studies of a family of novel XB and HB redox‐active, fluorescent anion receptors, this work not only provides detailed insights into the mechanisms of (redox‐modulated) fluorescence anion sensors, but also introduces a powerful spectroelectrochemical anion sensing methodology which is characterised by the concurrent read‐out of multiple optical and electrochemical sensing parameters.

Based on detailed fluorescence studies of the simpler **BDP_2_** or **BDP‐Ph** redox‐*inactive* receptors we first demonstrated halide anion sensing that is characterised by large emission turn‐ON in all cases, attributable to suppression of non‐radiative decay by receptor rigidification. The additional introduction of the redox‐active ferrocene into **BDP‐Fc** ⋅ **XB** induces a complete response reversal to fluorescence turn‐OFF, that arises from PET which is enhanced both kinetically and thermodynamically upon anion binding and which is strong enough to overcome the inherent turn‐ON response due to rigidification.

In contrast, the PET mechanism also induces quenching in **BDP‐Fc** ⋅ **HB**, which is however of such a magnitude that it cancels the inherent turn‐ON, leading to no overall anion responsiveness. The XB sensors thus not only display an expectedly higher anion binding affinity than the HB receptors but also enable directional anion response control (turn‐ON/OFF), a rare observation with potential implications in the design of advanced optical ion sensors, in particular ratiometric probes.

Importantly, the appended ferrocene also serves as an electrochemical voltammetric reporter for anion binding to **BDP‐Fc** ⋅ **XB/HB**, with cathodic shifts of the Fc/Fc^+^ *E*_1/2_ in the presence of anions. This redox process is not only associated with oxidation‐induced anion binding enhancement, but also significantly alters the fluorescence properties of the sensors. The high sensitivity of the BODIPY fluorophore on the redox‐state of the ferrocene then enabled the first proof‐of‐principle spectroelectrochemical sensing of bisulfate with **BDP‐Fc** ⋅ **HB**. Specifically, the fluorescence response of the sensor under rapid bulk electrolysis was sensitively dependent on the application of a cyclical potential perturbation, thereby enabling a conversion of a typical CV readout into the optical domain.

Thus, in one combined “fluorescence‐CV” experiment both the optical response of the sensor in two different oxidation states *and* the voltammetric response of the redox probe can be read out simultaneously, combining the advantages of both the optical technique (high sensitivity/low sensor concentration) and the electrochemical approach (redox‐modulation of binding and in situ generation of a charged receptor state). To the best of our knowledge this presents an entirely novel sensing approach that may not only enable the construction of increasingly sophisticated sensors but is also expected to be translatable to any sensor probe wherein the redox‐state of a redox‐reporter affects the fluorescence of an optical reporter.

## Supporting Information

The authors have cited additional references within the Supporting Information.^[29]^

## Conflict of interest

The authors declare no conflict of interest.

1

## Supporting information

As a service to our authors and readers, this journal provides supporting information supplied by the authors. Such materials are peer reviewed and may be re‐organized for online delivery, but are not copy‐edited or typeset. Technical support issues arising from supporting information (other than missing files) should be addressed to the authors.

Supporting Information

## Data Availability

The data that support the findings of this study are available from the corresponding author upon reasonable request.

## References

[1a] R. Hein , P. D. Beer , J. J. Davis , Chem. Rev. 2020, 120, 1888–1935; 31916758 10.1021/acs.chemrev.9b00624

[1b] P. A. Gale , C. Caltagirone , Chem. Soc. Rev. 2015, 44, 4212–4227. 24975326 10.1039/c4cs00179f

[2a] J. Y. C. Lim , P. D. Beer , Chem 2018, 4, 731–783;

[2b] J. Pancholi , P. D. Beer , Coord. Chem. Rev. 2020, 416, 213281;

[2c] S. Benz , M. Macchione , Q. Verolet , J. Mareda , N. Sakai , S. Matile , J. Am. Chem. Soc. 2016, 138, 9093–9096; 27433964 10.1021/jacs.6b05779

[2d] H. Zhao , F. P. Gabbaï , Nat. Chem. 2010, 2, 984–990; 20966957 10.1038/nchem.838

[2e] A. Borissov , I. Marques , J. Y. C. Lim , V. Félix , M. D. Smith , P. D. Beer , J. Am. Chem. Soc. 2019, 141, 4119–4129. 30730716 10.1021/jacs.9b00148

[3a] R. Hein , A. Borissov , M. D. Smith , P. D. Beer , J. J. Davis , Chem. Commun. 2019, 55, 4849–4852; 10.1039/c9cc00335e 30950463

[3b] S. C. Patrick , R. Hein , P. D. Beer , J. J. Davis , J. Am. Chem. Soc. 2021, 143, 19199–19206; 34730337 10.1021/jacs.1c09743

[3c] S. C. Patrick , R. Hein , A. Docker , P. D. Beer , J. J. Davis , Chem. Eur. J. 2021, 27, 10201–10209. 33881781 10.1002/chem.202101102 PMC8360193

[4a] R. Hein , A. Docker , J. J. Davis , P. D. Beer , J. Am. Chem. Soc. 2022, 144, 8827–8836; 35522996 10.1021/jacs.2c02924 PMC9121379

[4b] J. Y. Lim , I. Marques , V. Félix , P. D. Beer , Chem. Commun. 2018, 54, 10851–10854. 10.1039/c8cc06400h 30199082

[5] R. Hein , P. D. Beer , Chem. Sci. 2022, 13, 7098–7125. 35799814 10.1039/d2sc01800d PMC9214886

[6a] R. Hein , X. Li , P. D. Beer , J. J. Davis , Chem. Sci. 2021, 12, 2433–2440; 10.1039/d0sc06210c PMC8179314 34164009

[6b] R. Oliveira , S. Groni , C. Fave , M. Branca , F. Mavre , D. Lorcy , M. Fourmigue , B. Schollhorn , Phys. Chem. Chem. Phys. 2016, 18, 15867–15873; 27231819 10.1039/c6cp02219g

[6c] C. Fave , B. Schöllhorn , Curr. Opin. Electrochem. 2019, 15, 89–96;

[6d] H. Hijazi , A. Vacher , S. Groni , D. Lorcy , E. Levillain , C. Fave , B. Schollhorn , Chem. Commun. 2019, 55, 1983–1986; 10.1039/c8cc08856j 30687859

[6e] F. Zapata , A. Caballero , P. Molina , Eur. J. Inorg. Chem. 2017, 237–241.

[7a] B. R. Mullaney , A. L. Thompson , P. D. Beer , Angew. Chem. Int. Ed. 2014, 53, 11458–11462; 10.1002/anie.201403659 25044414

[7b] F. Zapata , A. Caballero , N. G. White , T. D. W. Claridge , P. J. Costa , V. t. Félix , P. D. Beer , J. Am. Chem. Soc. 2012, 134, 11533–11541; 22703526 10.1021/ja302213r

[7c] A. Docker , X. Shang , D. Yuan , H. Kuhn , Z. Zhang , J. J. Davis , P. D. Beer , M. J. Langton , Angew. Chem. Int. Ed. 2021, 60, 19442–19450; 10.1002/anie.202107748 PMC8456845 34185375

[7d] R. Kampes , R. Tepper , H. Görls , P. Bellstedt , M. Jäger , U. S. Schubert , Chem. Eur. J. 2020, 26, 14679–14687; 32686111 10.1002/chem.202002738 PMC7756348

[7e] Y. Cheong Tse , R. Hein , E. J. Mitchell , Z. Zhang , P. D. Beer , Chem. Eur. J. 2021, 27, 14550–14559; 34319624 10.1002/chem.202102493 PMC8596797

[7f] J. Y. Lim , I. Marques , V. Félix , P. D. Beer , Angew. Chem. 2018, 130, 593–597; 10.1002/anie.201711176 29178623

[7g] E. J. Mitchell , A. J. Beecroft , J. Martin , S. Thompson , I. Marques , V. Félix , P. D. Beer , Angew. Chem. Int. Ed. 2021, 60, 24048–24053; 10.1002/anie.202110442 PMC8596634 34494708

[7h] S. Mondal , A. Rashid , P. Ghosh , J. Organomet. Chem. 2021, 952, 122027.

[8a] A. K. A. Jaini , L. B. Hughes , M. M. Kitimet , K. J. Ulep , M. C. Leopold , C. A. Parish , ACS Sens. 2019, 4, 389–397; 30672707 10.1021/acssensors.8b01246

[8b] L. Cui , Y. Gong , X. Yu , C. Lv , X. Du , J. Zhao , Y. Che , ACS Sens. 2021, 6, 2851–2857; 34291907 10.1021/acssensors.1c01185

[8c] S. C. Patrick , R. Hein , M. Sharafeldin , X. Li , P. D. Beer , J. J. Davis , Chem. Eur. J. 2021, 27, 17700–17706; 34705312 10.1002/chem.202103249 PMC9297856

[8d] J. G. Weis , J. B. Ravnsbæk , K. A. Mirica , T. M. Swager , ACS Sens. 2016, 1, 115–119.

[9a] T. Gunnlaugsson , H. D. P. Ali , M. Glynn , P. E. Kruger , G. M. Hussey , F. M. Pfeffer , C. M. G. Dos Santos , J. Tierney , J. Fluoresc. 2005, 15, 287–299; 15986154 10.1007/s10895-005-2627-y

[9b] L. R. Ortega-Valdovinos , J. Valdes-García , I. J. Bazany-Rodríguez , J. C. Lugo-González , A. Dorazco-González , A. K. Yatsimirsky , New J. Chem. 2021, 45, 15618–15628;

[9c] G. W. Lee , N. Singh , D. O. Jang , Tetrahedron Lett. 2008, 49, 1952–1956;

[9d] E. A. Kataev , Chem. Commun. 2023, 59, 1717–1727. 10.1039/d2cc06194e 36722999

[10a] F. Zapata , A. Caballero , A. Tárraga , P. Molina , J. Org. Chem. 2010, 75, 162–169; 19968276 10.1021/jo9023446

[10b] T. Romero , A. Caballero , A. Tárraga , P. Molina , Org. Lett. 2009, 11, 3466–3469; 19572750 10.1021/ol901308z

[10c] M. Alfonso , A. Tárraga , P. Molina , Org. Lett. 2011, 13, 6432–6435; 22077401 10.1021/ol202723d

[10d] M. Alfonso , A. Espinosa Ferao , A. Tárraga , P. Molina , Inorg. Chem. 2015, 54, 7461–7473; 26171653 10.1021/acs.inorgchem.5b01071

[10e] D. Maity , S. Das , S. Mardanya , S. Baitalik , Inorg. Chem. 2013, 52, 6820–6838. 23724852 10.1021/ic3022326

[11a] D. Brunel , G. Noirbent , F. Dumur , Dyes Pigm. 2019, 170, 107611;

[11b] A. Loudet , K. Burgess , Chem. Rev. 2007, 107, 4891–4932. 17924696 10.1021/cr078381n

[12] N. Boens , V. Leen , W. Dehaen , Chem. Soc. Rev. 2012, 41, 1130–1172. 21796324 10.1039/c1cs15132k

[13] Note that a range of emission-spectroelectrochemical (ion) sensors have been developed, however these operate via fundamentally different transduction mechanisms. See for example:

[13a] S. Chatterjee , A. S. Del Negro , M. K. Edwards , S. A. Bryan , N. Kaval , N. Pantelic , L. K. Morris , W. R. Heineman , C. J. Seliskar , Anal. Chem. 2011, 83, 1766–1772; 21294535 10.1021/ac1030368

[13b] D. Martín-Yerga , A. Pérez-Junquera , D. Hernández-Santos , P. Fanjul-Bolado , Anal. Chem. 2017, 89, 10649–10654; 28892373 10.1021/acs.analchem.7b01734

[13c] D. Martín-Yerga , A. Pérez-Junquera , M. B. González-García , D. Hernández-Santos , P. Fanjul-Bolado , Anal. Chem. 2018, 90, 7442–7449; 29775045 10.1021/acs.analchem.8b00942

[13d] S. Chatterjee , M. S. Fujimoto , Y. H. Cheng , R. Kargupta , J. A. Soltis , R. K. Motkuri , S. Basuray , Sens. Actuators B 2019, 284, 663–674;

[13e] J. Garoz-Ruiz , J. V. Perales-Rondon , A. Heras , A. Colina , Electroanalysis 2019, 31, 1254–1278;

[13f] K. Węgrzyn , J. Kalisz , E. Stelmach , K. Maksymiuk , A. Michalska , Anal. Chem. 2021, 93, 10084–10089. 34264066 10.1021/acs.analchem.1c00857 PMC8382224

[14a] A. Docker , Y. C. Tse , H. M. Tay , A. J. Taylor , Z. Zhang , P. D. Beer , Angew. Chem. Int. Ed. 2022, 61, e202214523; 10.1002/anie.202214523 PMC10100147 36264711

[14b] A. J. Taylor , A. Docker , P. D. Beer , Chem. Asian J. 2023, 18, e202201170. 36516344 10.1002/asia.202201170 PMC10107604

[15a] T. K. Khan , R. R. Pissurlenkar , M. S. Shaikh , M. Ravikanth , J. Organomet. Chem. 2012, 697, 65–73;

[15b] O. Galangau , I. Fabre-Francke , S. Munteanu , C. Dumas-Verdes , G. Clavier , R. Méallet-Renault , R. Pansu , F. Hartl , F. Miomandre , Electrochim. Acta 2013, 87, 809–815;

[15c] X. Wu , W. Wu , X. Cui , J. Zhao , M. Wu , J. Mater. Chem. C 2016, 4, 2843–2853;

[15d] Y. V. Zatsikha , N. O. Didukh , T. Blesener , M. P. Kayser , Y. P. Kovtun , D. A. Blank , V. N. Nemykin , Eur. J. Inorg. Chem. 2017, 318–324.

[16] M. Albrecht , A. Lippach , M. P. Exner , J. Jerbi , M. Springborg , N. Budisa , G. Wenz , Org. Biomol. Chem. 2015, 13, 6728–6736. 25994282 10.1039/c5ob00505a

[17] H. Gallardo , A. J. Bortoluzzi , D. M. P. De Oliveira Santos , Liq. Cryst. 2008, 35, 719–725.

[18] Y. V. Zatsikha , N. O. Didukh , R. K. Swedin , V. P. Yakubovskyi , T. S. Blesener , A. T. Healy , D. E. Herbert , D. A. Blank , V. N. Nemykin , Y. P. Kovtun , Org. Lett. 2019, 21, 5713–5718. 31283252 10.1021/acs.orglett.9b02082

[19a] M. K. Kuimova , G. Yahioglu , J. A. Levitt , K. Suhling , J. Am. Chem. Soc. 2008, 130, 6672–6673; 18457396 10.1021/ja800570d

[19b] S.-C. Lee , J. Heo , H. C. Woo , J.-A. Lee , Y. H. Seo , C.-L. Lee , S. Kim , O.-P. Kwon , Chem. Eur. J. 2018, 24, 13706–13718. 29700889 10.1002/chem.201801389

[20] Y. V. Zatsikha , E. Maligaspe , A. A. Purchel , N. O. Didukh , Y. Wang , Y. P. Kovtun , D. A. Blank , V. N. Nemykin , Inorg. Chem. 2015, 54, 7915–7928. 26220063 10.1021/acs.inorgchem.5b00992

[21] The fluorescence lifetimes of the HB receptors are too short to be quantitatively analysed, but show the same qualitative trends.

[22] J. Ding , K. Feng , C.-H. Tung , L.-Z. Wu , J. Phys. Chem. C 2011, 115, 833–839.

[23] Y. V. Zatsikha, T. S. Blesener, A. J. King, A. T. Healy, P. C. Goff, N. O. Didukh, D. A. Blank, Y. P. Kovtun, V. N. Nemykin, *J. Phys. Chem. B* **2021**, *125*, 360–371. 10.1021/acs.jpcb.0c10074 33370123

[24a] K. Vandewal , J. Benduhn , V. C. Nikolis , Sustain. Energy Fuels 2018, 2, 538–544;

[24b] R. Marcus , J. Phys. Chem. 1989, 93, 3078–3086.

[25a] J. Y. C. Lim , M. J. Cunningham , J. J. Davis , P. D. Beer , Chem. Commun. 2015, 51, 14640–14643; 10.1039/c5cc05704c 26289779

[25b] J. Y. C. Lim , P. D. Beer , Eur. J. Inorg. Chem. 2017, 220–224.

[26a] X.-B. Xia , Z.-F. Ding , J.-Z. Liu , J. Photochem. Photobiol. A 1995, 88, 81–84;

[26b] J. Gan , H. Tian , Z. Wang , K. Chen , J. Hill , P. A. Lane , M. D. Rahn , A. M. Fox , D. D. C. Bradley , J. Organomet. Chem. 2002, 645, 168–175;

[26c] M. Tropiano , N. L. Kilah , M. Morten , H. Rahman , J. J. Davis , P. D. Beer , S. Faulkner , J. Am. Chem. Soc. 2011, 133, 11847–11849; 21761827 10.1021/ja203069s

[26d] J. Lehr , M. Tropiano , P. D. Beer , S. Faulkner , J. J. Davis , Chem. Commun. 2015, 51, 6515–6517. 10.1039/c5cc01097g 25773962

[27] O. Alévêque , E. Levillain , Luminescence in Electrochemistry: Applications in Analytical Chemistry, Physics and Biology, Springer, Berlin, 2017, pp. 1–19.

[28] Note that the observed changes upon anion addition cannot arise from signal decay, as the initial fluorescence intensity is regained at the cathodic vertex in each run.

[29a] C. Würth , M. Grabolle , J. Pauli , M. Spieles , U. Resch-Genger , Nat. Protoc. 2013, 8, 1535–1550; 23868072 10.1038/nprot.2013.087

[29b] K. M. Bąk , S. C. Patrick , X. Li , P. D. Beer , J. J. Davis , Angew. Chem. Int. Ed. 2023, 62, e202300867; 10.1002/anie.202300867 PMC10946961 36749115

[29c] A. S. K. Hashmi , R. Döpp , C. Lothschütz , M. Rudolph , D. Riedel , F. Rominger , Adv. Synth. Catal. 2010, 352, 1307–1314;

[29d] S. Toliautas , J. Dodonova , A. Žvirblis , I. Čiplys , A. Polita , A. Devižis , S. Tumkevičius , J. Šulskus , A. Vyšniauskas , Chem. Eur. J. 2019, 25, 10342–10349. 30998263 10.1002/chem.201901315

