# Supplementary material for: Anion Sensing through Redox‐Modulated Fluorescent Halogen Bonding and Hydrogen Bonding Hosts
Source: Angew Chem Int Ed Engl. 2024 Jan 4;63(6):e202315959. doi: 10.1002/anie.202315959 (PMC10952190; doi:10.1002/anie.202315959)
Supplement: Supplementary file 1 — Supporting Information [file ANIE-63-0-s001.pdf]

Angewandte Chemie
Eine Zeitschrift der Gesellschaft Deutscher Chemiker

Supporting Information

Anion Sensing through Redox-Modulated Fluorescent Halogen Bonding and Hydrogen Bonding Hosts

A. J. Taylor, R. Hein, S. C. Patrick, J. J. Davis, P. D. Beer**

Supporting Information

Table of Contents

1. Instrumentation and General Experimental Details 2

2. Synthesis and Characterisation of Compounds 6

3. Effect of Solvent Viscosity on BDP-Ph•HB 35

4. ^{1}H NMR Titration of BDP-Fc•HB with TBABr 36

5. Fluorescence Lifetime Measurements... 38

6. Voltammetric Characterisation of BDP-Fc•XB/HB.. 40

7. Gibbs Free Energy of Electron Transfer 42

8. Voltammetric Titrations... 44

9. Bisulfate binding studies with neutral HB receptors 46

References 49

1. Instrumentation and General Experimental Details

General Information

All commercially available chemicals and solvents were used as received without further purification. Dry solvents were degassed with N_2 and dried on a Mbraun MPSP-800 column. Ultrapure water was obtained from a Milli-Q system (18.2 MΩcm). Mass spectrometry was performed on a Bruker micrOTOF. NMR spectra were recorded on Bruker NMR spectrometers (AVIII HD 500 or AVIII HD 400). Chromatography was performed using silica gel (particle size: 40-63 µm). All data analysis and fitting was carried out with OriginPro 2017.

Optical Measurements

UV-vis and fluorescence measurements were carried out on a Duetta (Horiba) using quartz cuvettes with a path length of 10 nm. Unless otherwise noted, all fluorescence spectra were acquired with a wavelength of excitation of 485 nm, 5 nm excitation and emission slits and were recorded in, at least, triplicate repeat measurements to ensure signal stability. Anion titration studies were carried out by titrating a 1 µM solution of the receptor with aliquots of a concentrated solution of TBA-anion in the same receptor solution to ensure a constant receptor concentration.

Fluorescence lifetimes were measured on an Edinburgh Instruments FS5 spectrofluorometer operating Fluoracle® software, using in time-correlated single photon counting (TCSPC) mode using a picosecond pulsed diode laser (EPL-475) as the excitation source. The detector was a R13456 PMT detector (200–950 nm spectral coverage, Hamamatsu). Measurements were conducted at 298 K.

Spectroscopic grade acetone and acetonitrile (Alfa Aesar) was used throughout.

Determination of Quantum Yields

Fluorescence quantum yields were determined according to established procedures reported by Resch-Genger and coworkers.[1]

Briefly, the quantum yield was calculated according to equation S1 relative to fluorescein in 0.1 M NaOH as standard (Φ_{St} = 0.89).[1]

$$\theta_{BDP} = \theta_{St} \frac{F_{BDP}}{F_{St}} \frac{f_{St}}{f_{BDP}} \frac{n_{BDP}^2}{n_{St}^2} \qquad \text{(eqn. S1)}$$

Where F is the integral photon flux of the standard and the BODIPY receptor, Φ_{St} the quantum yield of the standard, n the refractive index of the solvent and $f = 1 - 10^{-A_{Exc}}$. With n_{BDP} (acetone) = 1.3586 and n_{St} (water) = 1.3300.

Identical measurement parameters were used for both the standard and receptor samples: λ_{Exc} = 465 nm, 5/5 nm emission and excitation slits, 20 accumulations. The emission was measured, and integrated, in the range between 475 – 850 nm. The absorbance at the excitation wavelength A_{465} was kept around ≈0.1 for both the BODIPY samples and the standard. Both absorbance and emission were measured multiple times to ensure signal stability. All quantum yields were rounded to the nearest percent.

Electrochemical Measurements

All experiments were carried out with an Autolab Potentiostat (Metrohm) or a PalmSens4 Potentiostat with a three-electrode setup comprising a glassy carbon (GC) working electrode (3 mm, BASi), a Pt wire counter electrode and a Ag|$AgNO_3$ reference electrode (with an inner filling solution of 10 mM $AgNO_3$, 100 mM $TBAClO_4$ in ACN). Prior to each experiment the GC working electrode was mechanically polished with an alumina slurry (0.05 µm particle size), followed by sonication in 1:1 H_2O/EtOH, prior to each experiment. 100 mM $TBAPF_6$ or 100 mM $TBAClO_4$ were used as supporting electrolyte, as indicated. Cyclic voltammograms (CVs) were recorded using a step potential of 2.4 mV and at a scan rate of 100 mV/s, unless otherwise noted. Square wave voltammograms (SWVs) were recorded using a step potential of 2 mV, a 20 mV amplitude and at a frequency of 25 Hz. The receptors` half-wave potentials ($E_{1/2}$) were determined as the peak potential by SWV and were referenced to Fc as an internal standard.

Voltammetric titration experiments were carried at a constant receptor concentration of 0.1 mM or 0.25 mM, as indicated. In all cases the overall ionic strength was kept constant (at

100 mM) by titration of an initial host solution containing 100 mM $TBAClO_4$ with an anion solution in the same solvent system containing 100 mM TBA-anion. In select cases up to 200 µM aqueous $HClO_4$ was also added to the electrolyte system to stabilise the ferrocenium redox state, as required for spectroelectrochemical experiments.[2]

All electrochemical titrations were followed by SWV. Iodide was not tested as an anion in these voltammetric titrations as its inherent redox activity overlaps with that of the ferrocene reporter group. We instead included HSO_4^- as an additional analyte.

Fluorescence-Spectroelectrochemical Measurements

Spectroelectrochemical experiments were conducted in a standard quartz fluorescence cuvette (1 x 1 x 3 cm), using a 3D printed cap, which held another three-electrode setup (Pt grid working electrode, non-aqueous Ag|$AgNO_3$ reference electrode (as above), Pt wire counter electrode) in the solution but above the light path. The solution was stirred rapidly throughout measurements, using a stirrer bar below the light path (Figure S1). The sample was excited at 485 nm, and the emission at 521 nm was continually monitored throughout all experiments. Reversible oxidation and reduction over multiple cycles was representatively demonstrated for **BDP-Fc•XB** (Figure 6A) upon application of alternating oxidative and reductive potentials, whereby the potentials were each at least 200 mV anodic or cathodic of the $E_{1/2}$ respectively. Fluorescence CV scans and anion binding titrations on **BDP-Fc•HB** were carried out at a scan rate of 0.75 mV/s. Fluorescence CV scans were conducted in ACN, containing 100 mM $TBAClO_4$, 200 µM $HClO_4$ and 0.02% H_2O. Data for fluorescence CV scans were smoothed using a 5-point weighted moving average method. $E_{1/2}$ values from fluorescence CVs were obtained by finding the value of E at the midpoint between the minimum and maximum fluorescence intensities for the forward and backward scan and taking the arithmetic mean.

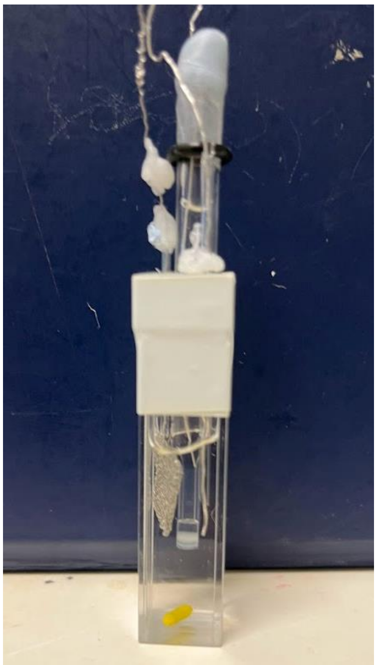

Figure S1. The cuvette used for spectroelectrochemical experiments. All electrodes were held in place with a 3D-printed holder such that they did not reach into the light path.

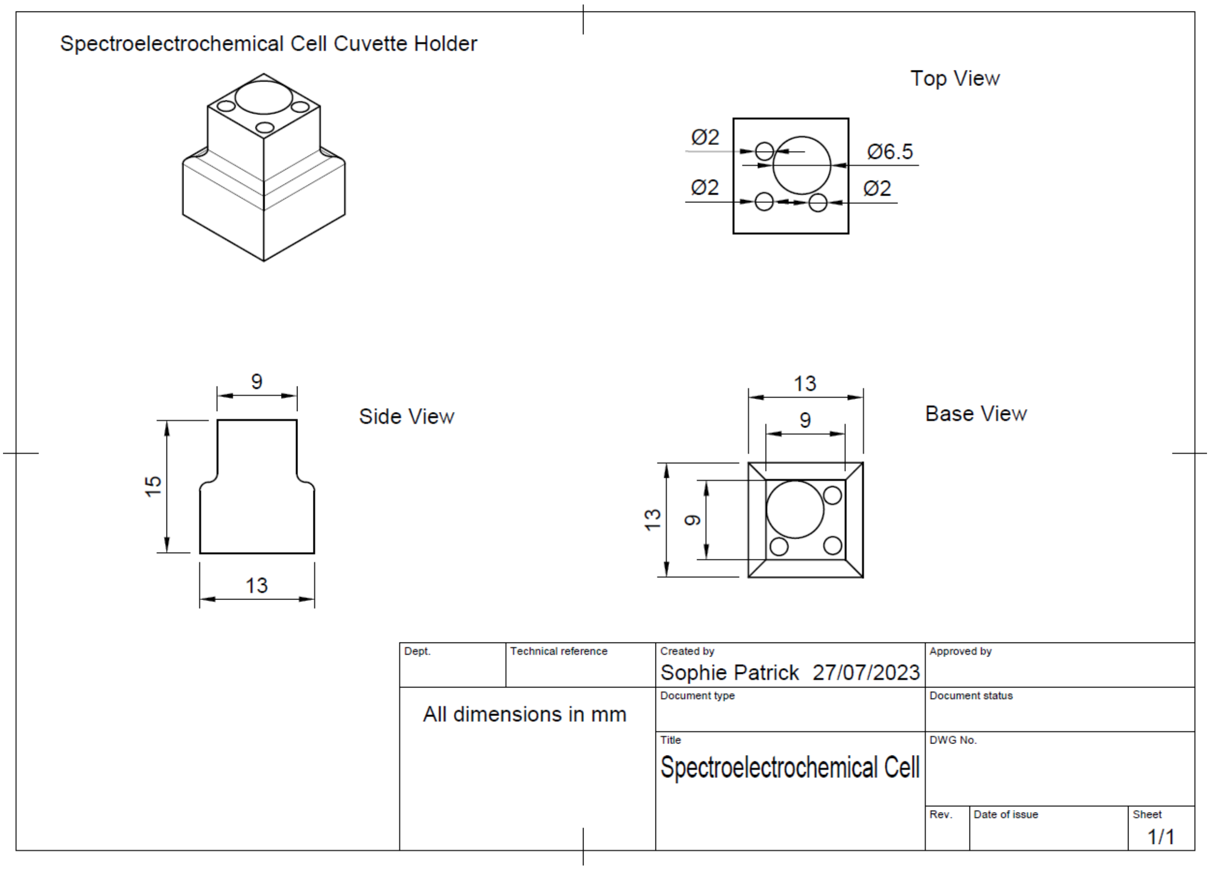

Figure S2. Blueprint for 3D-printed holder for spectroelectrochemical experiments.

2. Synthesis and Characterisation of Compounds

Synthesis of HB Analogues

8-ethynyl-BODIPY[3] was subjected to typical CuAAC ('click') conditions with 1,3-diazidobenzene,[4] statistically producing the mono-click product **5** in 42% yield (Scheme S1). **5** was then resubjected to CuAAC conditions with either ethynylferrocene, 8-ethynyl-BODIPY or phenylacetylene to afford **BDP-Fc•HB**, **BDP$_2$•HB**, and **BDP-Ph•HB** in 98%, 86% and 99% yields respectively (Scheme 2).

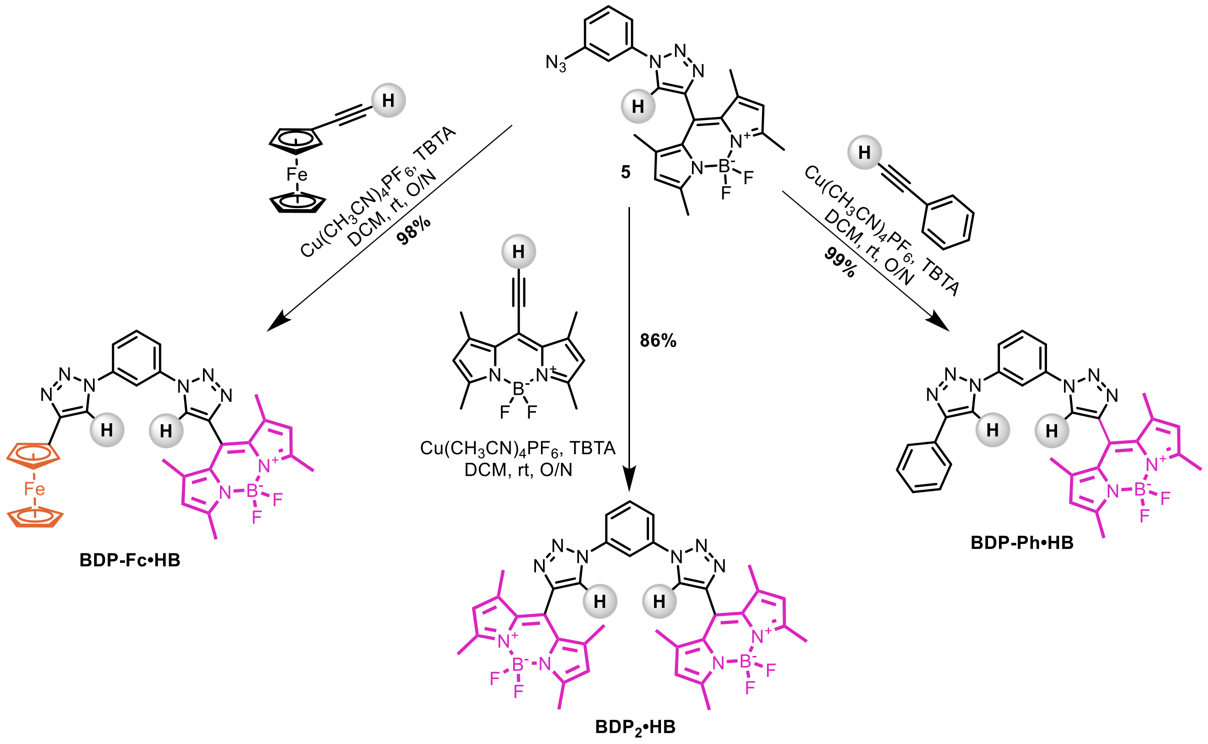

Scheme S1. Synthesis of the title compounds **BDP-Fc•HB, BDP$_2$•HB,** and **BDP-Ph•HB.**

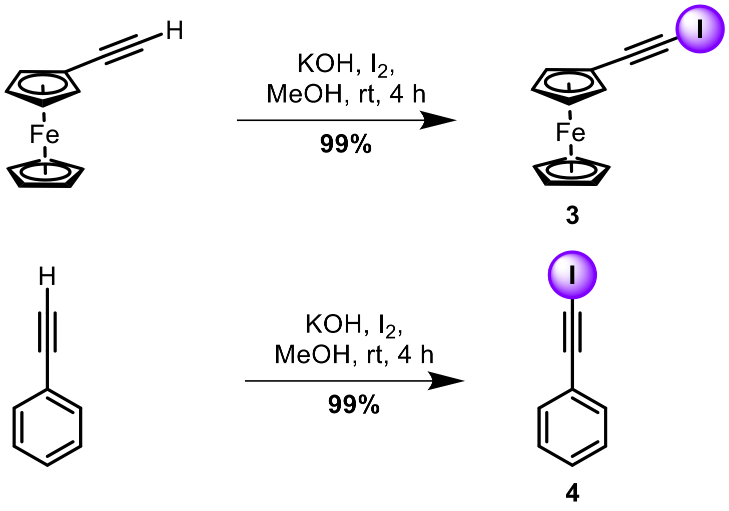

Scheme S2. Synthesis of iodo-alkynes **3** and **4.**

General Synthetic Procedure 1

$Cu(MeCN)_4PF_6$ (0.04 mmol) and TBTA (0.04 mmol) were dissolved in degassed CH_2Cl_2 (≈ 2 mL) and left to stir for 10 minutes. The requisite alkyne precursor (**1** or 8-ethynyl-BODIPY) (0.37 mmol) was added to the solution of the copper complex, followed by 1,3-diazidobenzene (0.75 mmol). The resultant mixtures were left to stir until TLC analysis confirmed full consumption of the alkyne precursor. The crude reaction mixture was diluted with CH_2Cl_2 (15 mL) and the organic layer washed with aqueous 0.01 M NH_4OH/EDTA solution (10 mL). The resultant aqueous layer was back extracted with CH_2Cl_2 (2 x 10 mL) and the combined organic phases washed with water before they were dried over $MgSO_4$ and concentrated in vacuo to obtain the crude product mixture. The relevant products were isolated via silica-gel chromatography (DCM/EtOAc).

General Synthetic Procedure 2

$Cu(MeCN)_4PF_6$ (0.02 mmol) and TBTA (0.02 mmol) were dissolved in degassed CH_2Cl_2 (≈ 1 mL) and left to stir for 10 minutes. The requisite alkyne precursor (**1**, **3**, **4**, ethynylferrocene, 8-ethynyl-BODIPY or phenylacetylene) (0.15 mmol) was added to the solution of the copper complex, followed by the azide coupling partner (**2** or **5**) (0.15 mmol). The resultant mixtures were left to stir until TLC analysis confirmed full consumption of the alkyne precursor. The crude reaction mixture was diluted with CH_2Cl_2 (15 mL) and the organic layer washed with

aqueous 0.01 M NH_4OH/EDTA solution (10 mL). The resultant aqueous layer was back extracted with CH_2Cl_2 (2 x 10 mL) and the combined organic phases washed with water before they were dried over $MgSO_4$ and concentrated in vacuo to obtain the crude product mixture. The relevant products were isolated via silica-gel chromatography (DCM/EtOAc).

8-(iodo)ethynyl-BODIPY 1

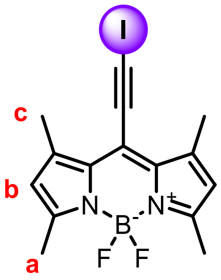

TMS protected 8-ethynyl-BODIPY[3] (60 mg, 0.174 mmol, 1 equiv.) and AgF (22 mg, 0.174 mmol, 1 equiv.) were dissolved in dry degassed MeCN (3 mL). NIS (42 mg, 0.174 mmol, 1 equiv.) was added and the reaction mixture left to stir in darkness for 5 h. The mixture was filtered through a silica plug and the filtrate reduced in vacuo. The crude was dissolved in DCM (20 mL) and washed with H_2O (3 x 20 mL) before drying over $MgSO_4$. The solvent was removed in vacuo and the product was purified by column chromatography (EtOAc/hexanes) and isolated as a red powder (69 mg, 94%).

^{1}H NMR (500 MHz, $CDCl_3$) δ 6.06 (s, 2H, H_b), 2.52 (s, 6H, H_c), 2.41 (s, 6H, H_a).

^{13}C NMR (151 MHz, $CDCl_3$) δ 155.05, 142.21, 134.11, 121.07, 119.69, 90.49, 28.44, 15.35, 14.80.

HRMS – *unable to obtain due to decomposition*.

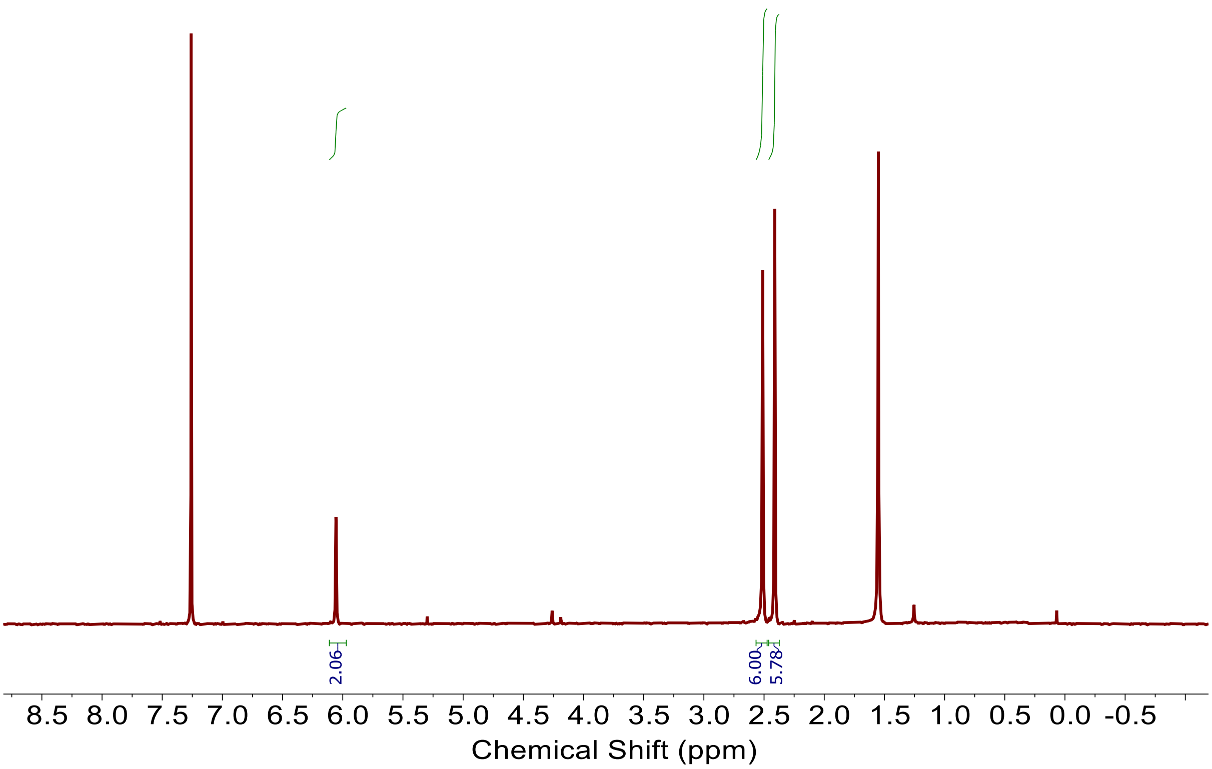

Figure S3. ^{1}H NMR spectrum of **1.**

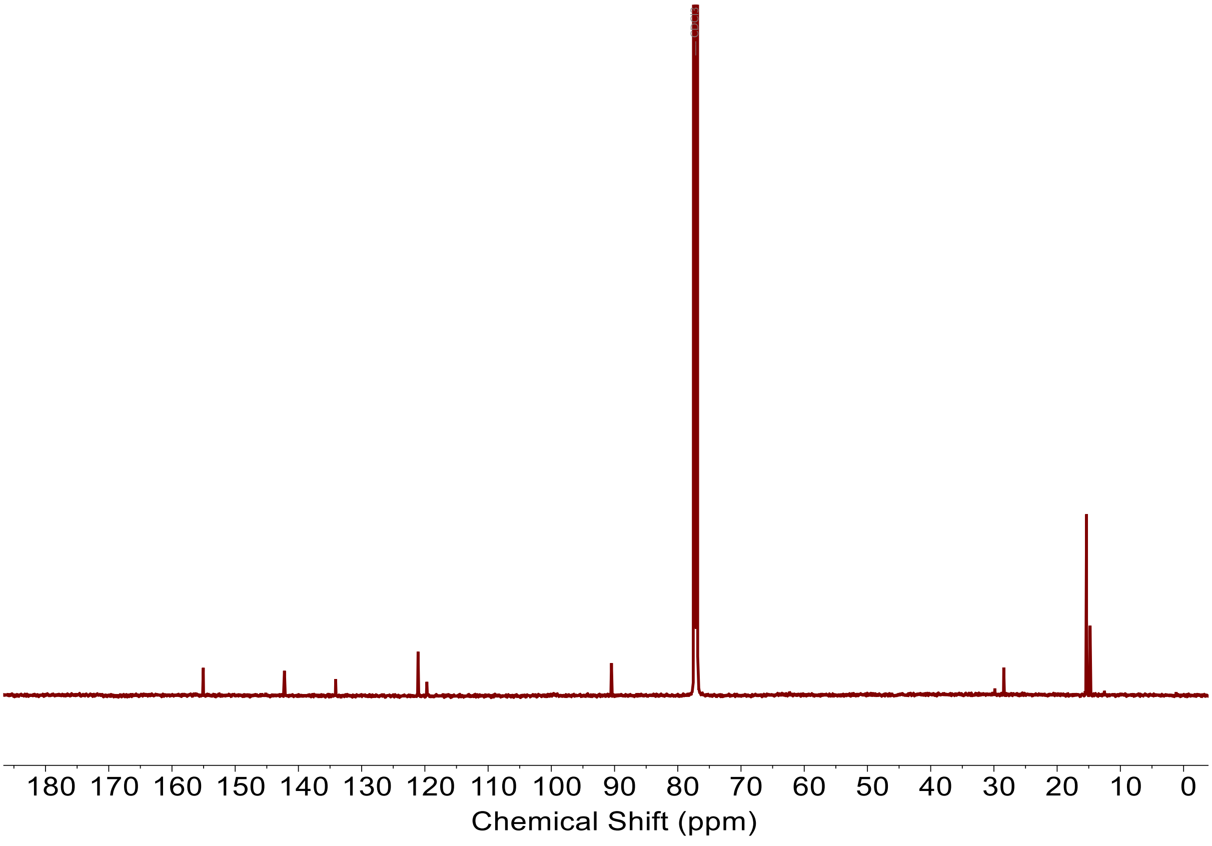

Figure S4. ^{13}C NMR spectrum of **1.**

XB Synthon 2

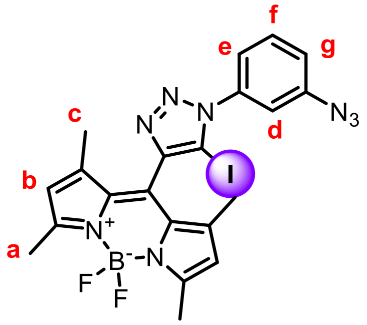

Synthon **2** was prepared from 1,3-diazidobenzene and **1** according to General Synthetic Procedure 1, affording **2** in 40% yield.

^{1}H NMR (400 MHz, $CDCl_3$) δ 7.60 (t, *J* = 8.1 Hz, 1H, H_f), 7.34 (dd, 1H, H_e), 7.30 (dd, 1H, H_g), 7.22 (t, *J* = 2.1 Hz, 1H, H_d), 6.05 (s, 2H, H_b), 2.58 (s, 6H, H_c). *H_a not observed as coincident with residual H_2O peak.*

^{13}C NMR (151 MHz, $CDCl_3$) δ 157.43, 146.76, 142.27, 141.95, 137.66, 132.25, 130.85, 127.16, 122.10, 121.79, 120.83, 116.75, 82.39, 14.84, 13.84.

HRMS (ESI +ve) m/z: 559.0833, ([M+H]+, $C_{21}H_{19}BF_2IN_8$ requires 559.0835).

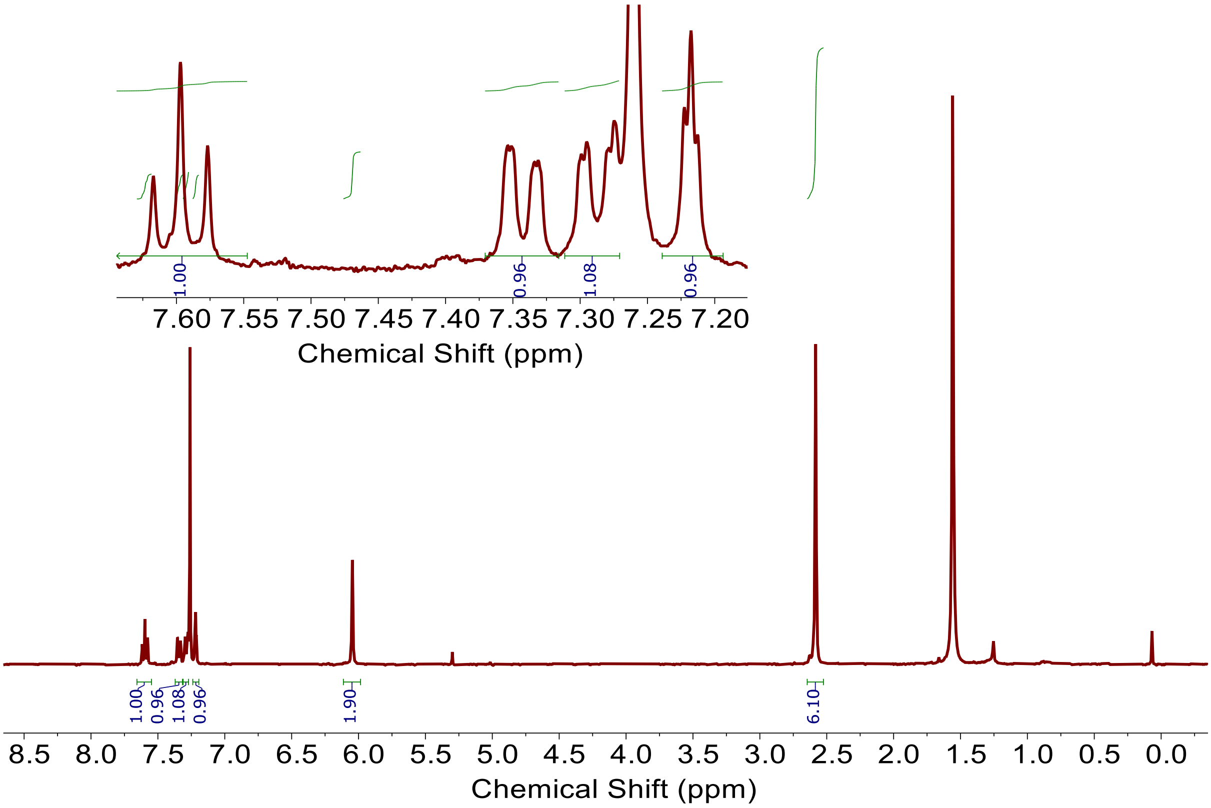

Figure S5. ^{1}H NMR spectrum of **2.**

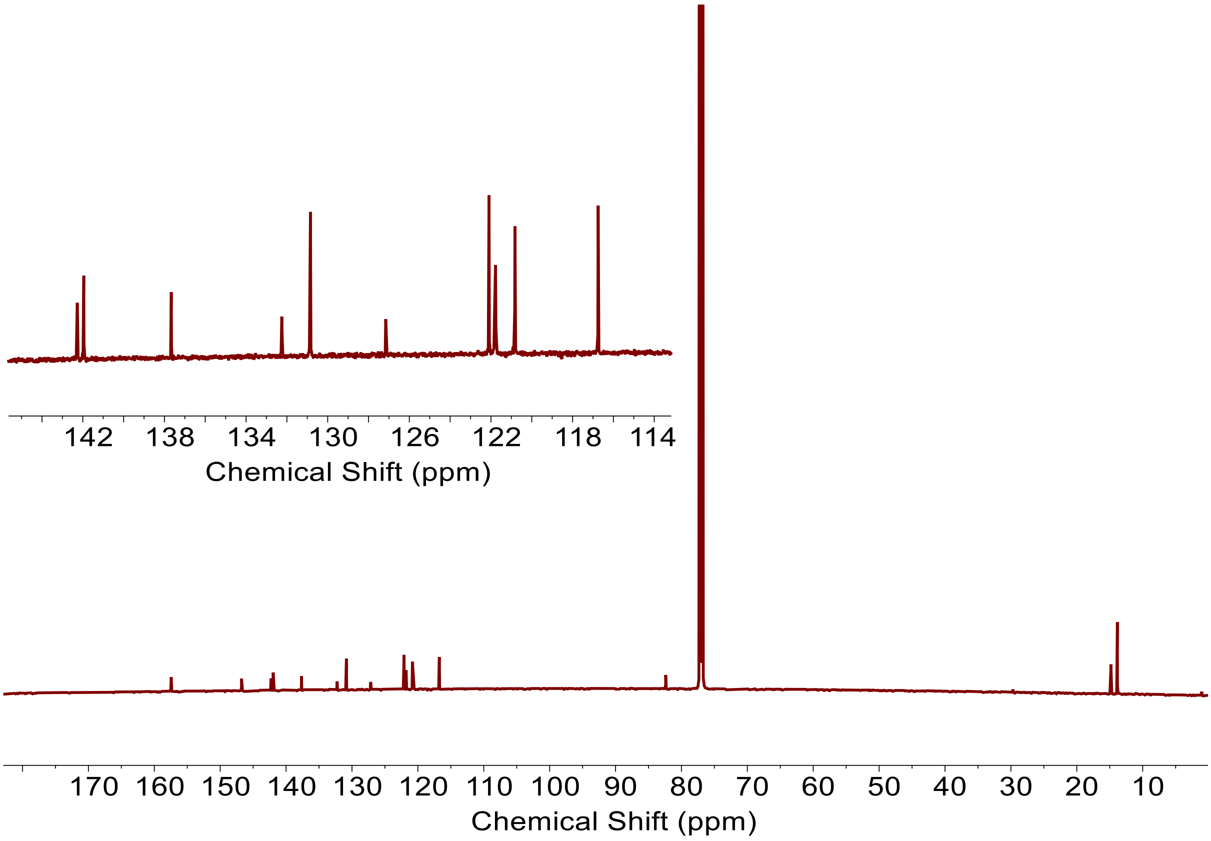

Figure S6. ^{13}C NMR spectrum of **2.**

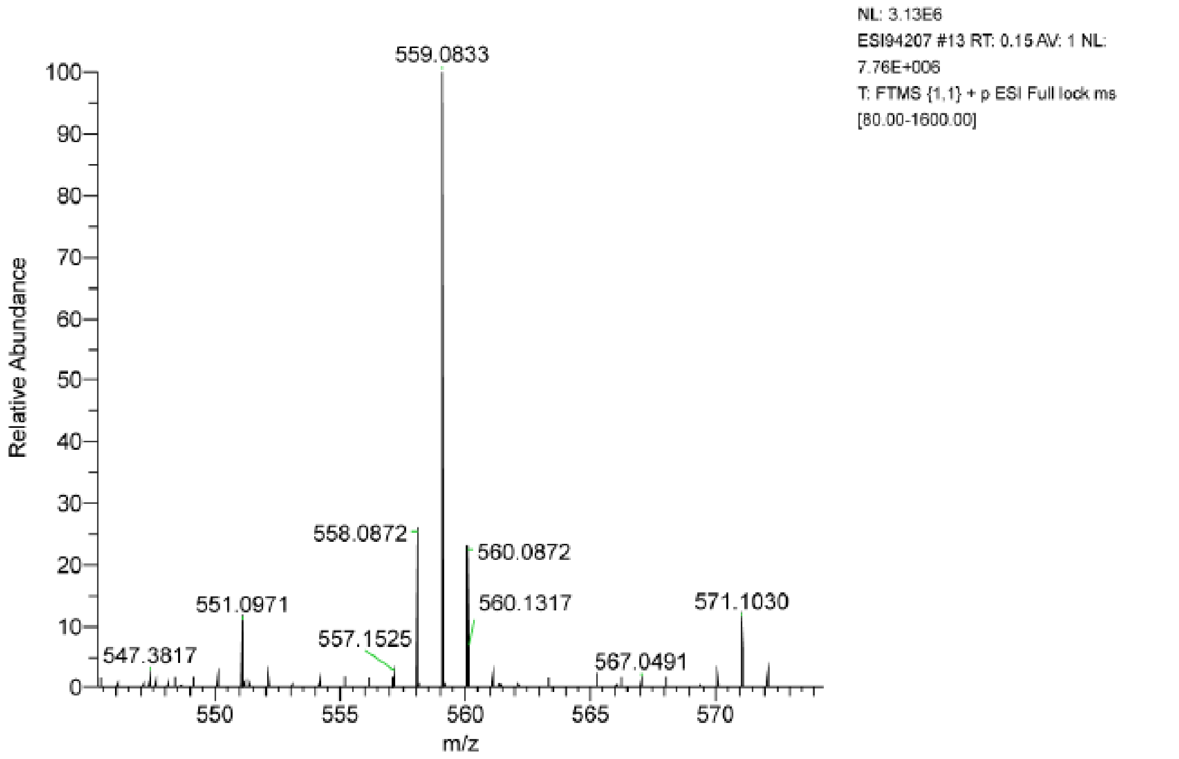

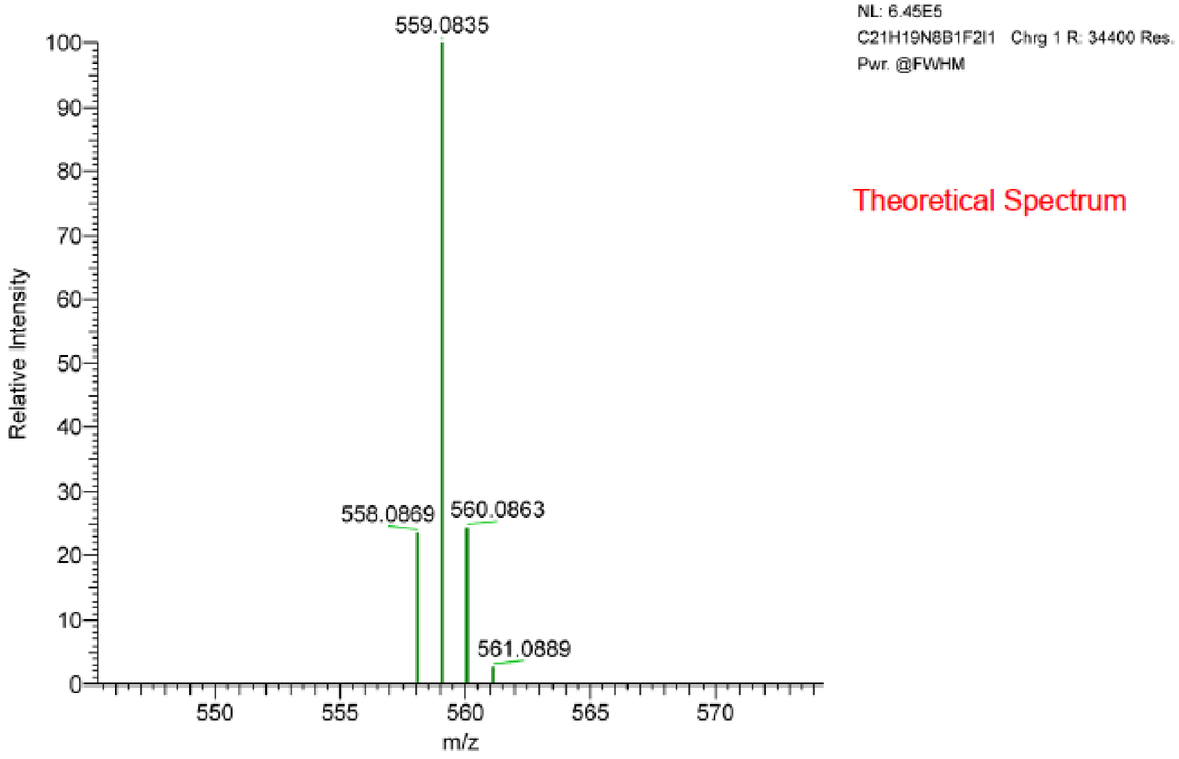

Figure S7. Experimental and theoretical mass spectra of **2**.

XB Synthon 3

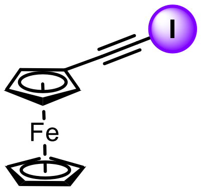

Synthon **3** was prepared from ethynylferrocene as previously reported.[5] ^{1}H NMR and MS was in accordance with previously published data.

XB Synthon 4

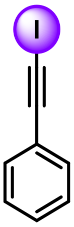

Synthon **4** was prepared from phenylacetylene as previously reported.[6] ^{1}H NMR and MS was in accordance with previously published data.

HB Synthon 5

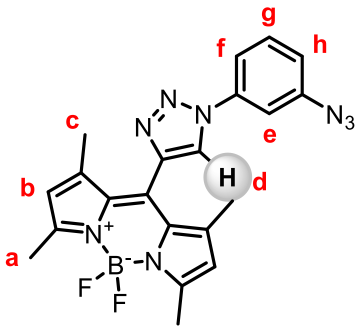

Synthon **5** was prepared from 1,3-diazidobenzene and 8-ethynyl-BODIPY according to General Synthetic Procedure 1, affording **5** in 42% yield.

^{1}H NMR (400 MHz, $CDCl_3$) δ 8.04 (s, 1H, H_d), 7.60 – 7.48 (m, 3H, $H_{f,g,h}$), 7.21 – 7.14 (m, 1H, H_e), 6.02 (s, 2H, H_b), 2.56 (s, 6H, H_c), 1.54 (s, 6H, H_a).

^{13}C NMR (151 MHz, $CDCl_3$) δ 157.17, 142.85, 142.58, 141.43, 137.92, 132.53, 131.48, 127.93, 121.89, 121.49, 119.69, 116.41, 111.46, 14.90, 14.61.

HRMS (ESI +ve) m/z: 433.1878, ([M+H]+, $C_{21}H_{20}BF_2N_8$ requires 433.1868).

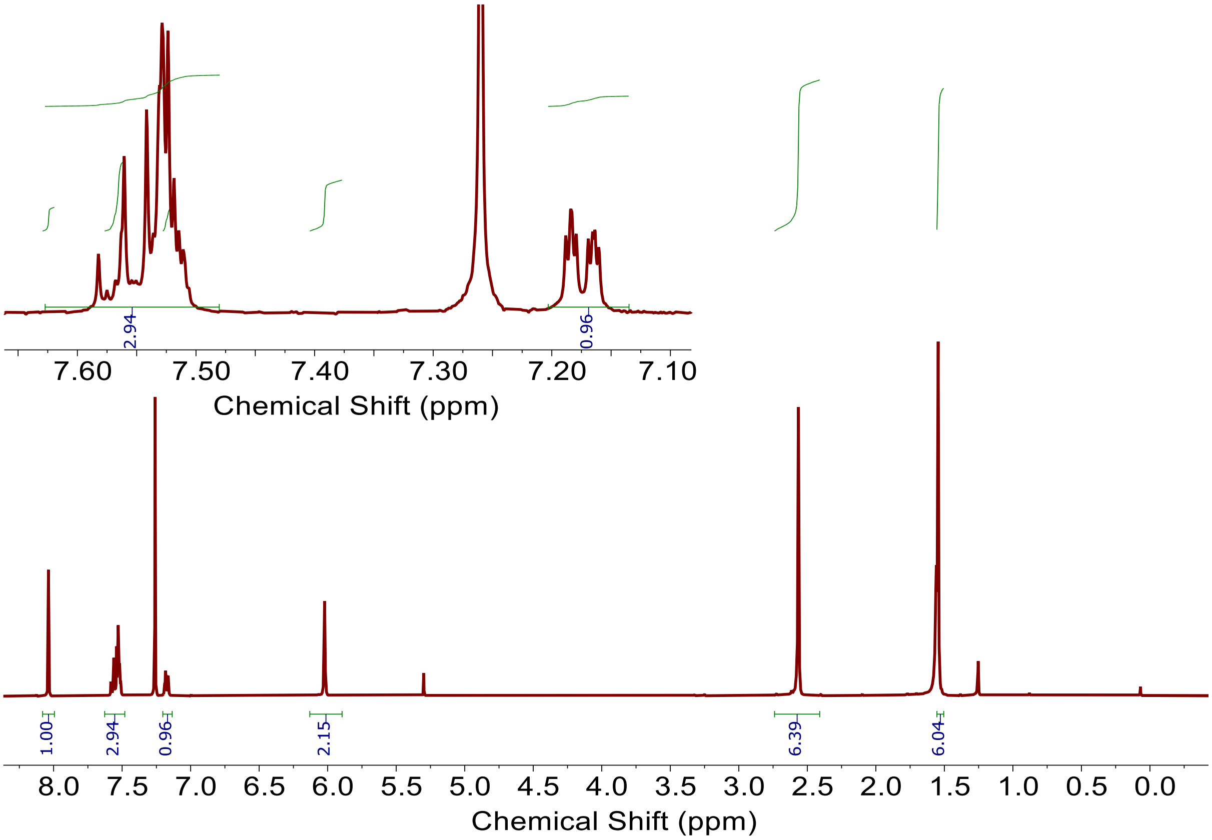

Figure S8. ^{1}H NMR spectrum of **5.**

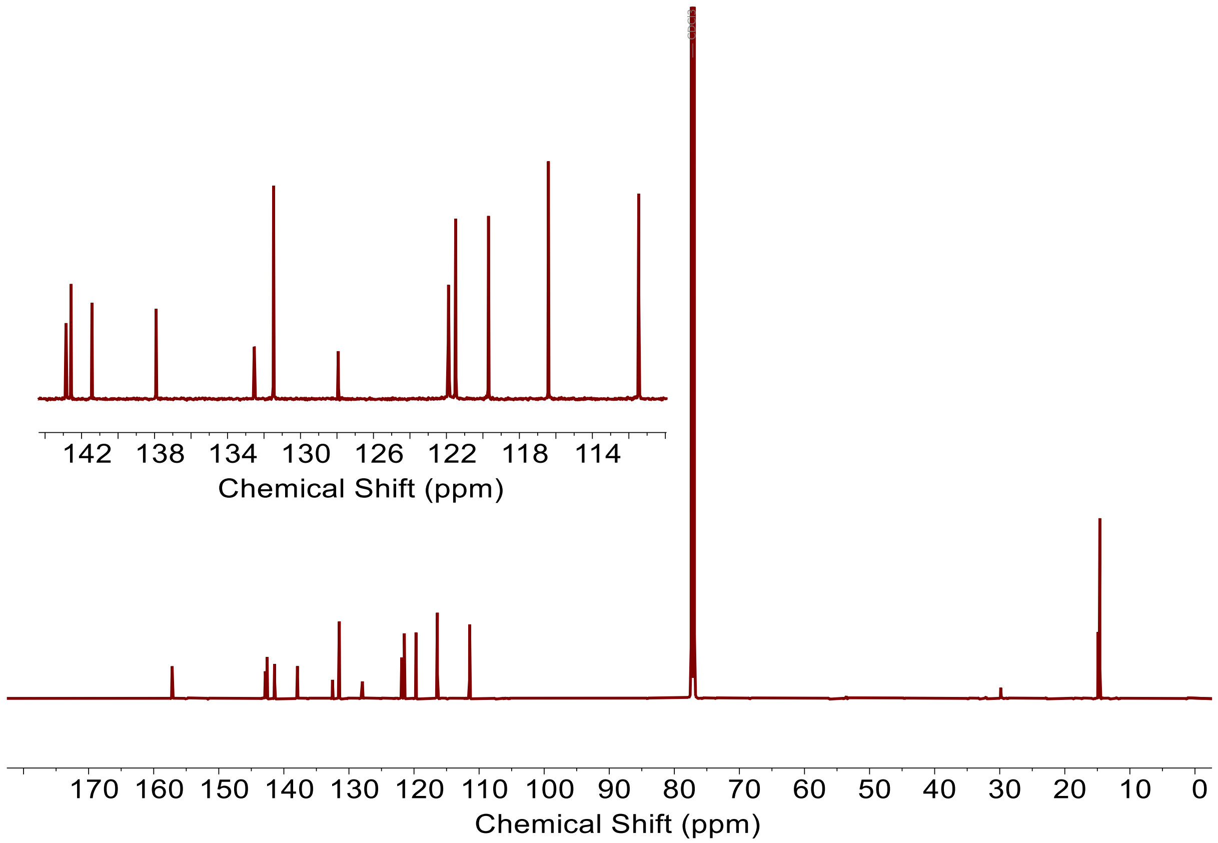

Figure S9. ^{13}C NMR spectrum of **5.**

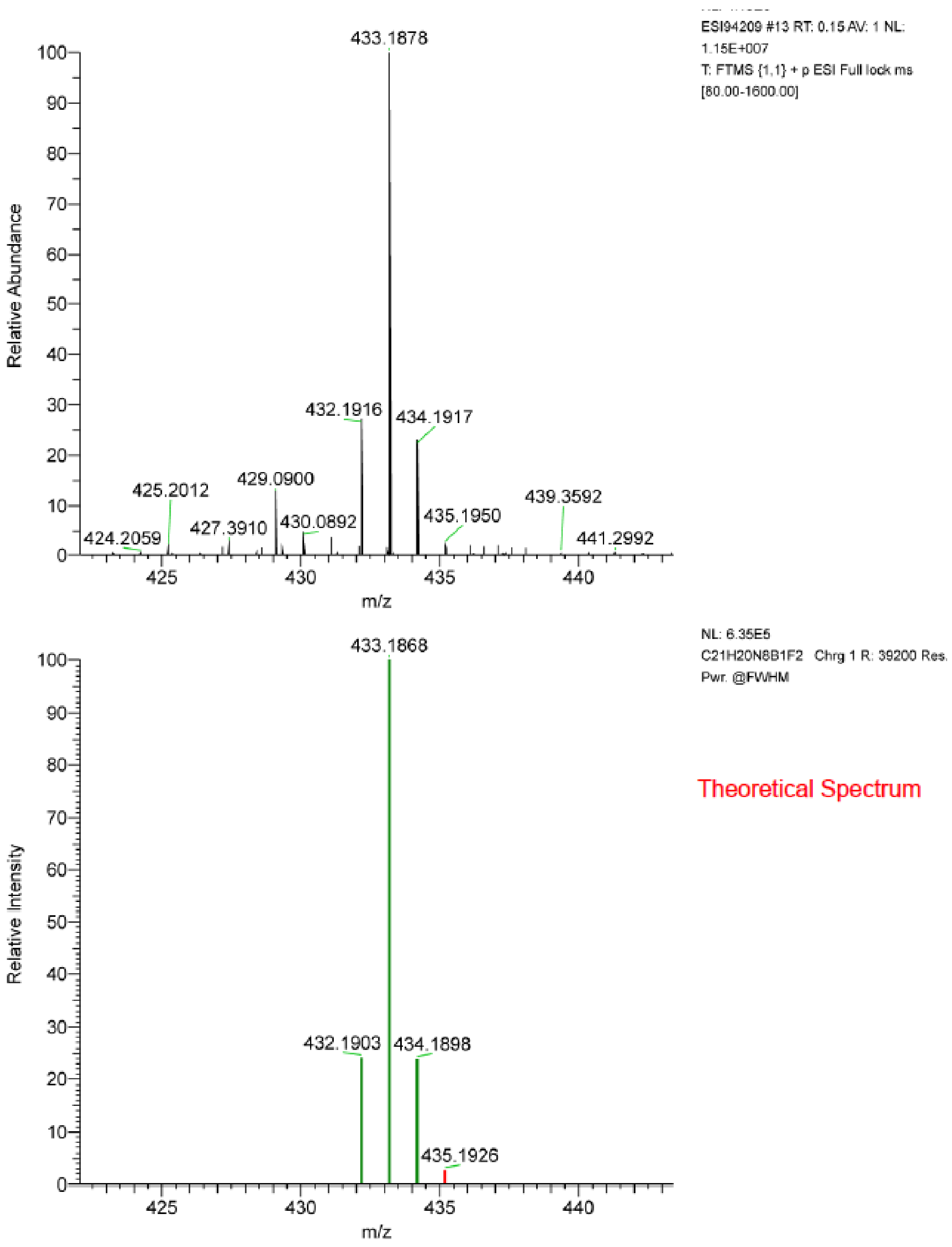

Figure S10. Experimental and theoretical mass spectra of **5**.

BDP-Fc•HB

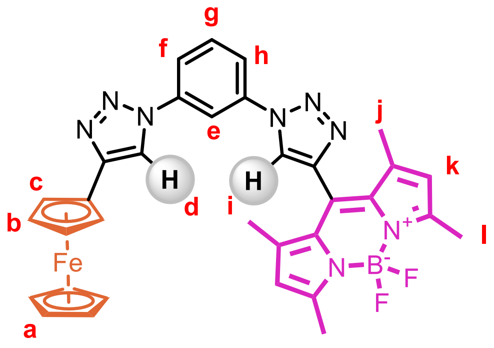

BDP-Fc•HB was prepared from **5** and ethynylferrocene according to General Synthetic Procedure 2, affording **BDP-Fc•HB** in 98% yield.

^{1}H NMR (600 MHz, DMSO-d_6) δ 9.29 (s, 1H, H_i), 9.07 (s, 1H, H_d), 8.60 (t, J = 2.1 Hz, 1H, H_e), 8.13 (m, 2H, $H_{f,h}$), 7.90 (t, J = 8.2 Hz, 1H, H_g), 6.27 (s, 2H, H_k), 4.82 (t, J = 1.9 Hz, 2H, H_c), 4.38 (t, J = 1.9 Hz, 2H, H_b), 4.11 (s, 5H, H_a), 1.55 (s, 6H, H_l). *H_c not observed as coincident with residual DMSO peak.*

^{13}C NMR (151 MHz, DMSO-d_6) δ 156.39, 147.03, 142.87, 139.64, 137.72, 137.28, 131.81, 131.58, 128.69, 124.02, 121.89, 119.98, 119.74, 118.68, 111.48, 74.96, 69.40, 69.38, 68.66, 66.50, 14.39, 14.00.

HRMS (ESI +ve) m/z: 643.1987, ([M+H]+, $C_{33}H_{30}BF_2FeN_8$ requires 643.1998).

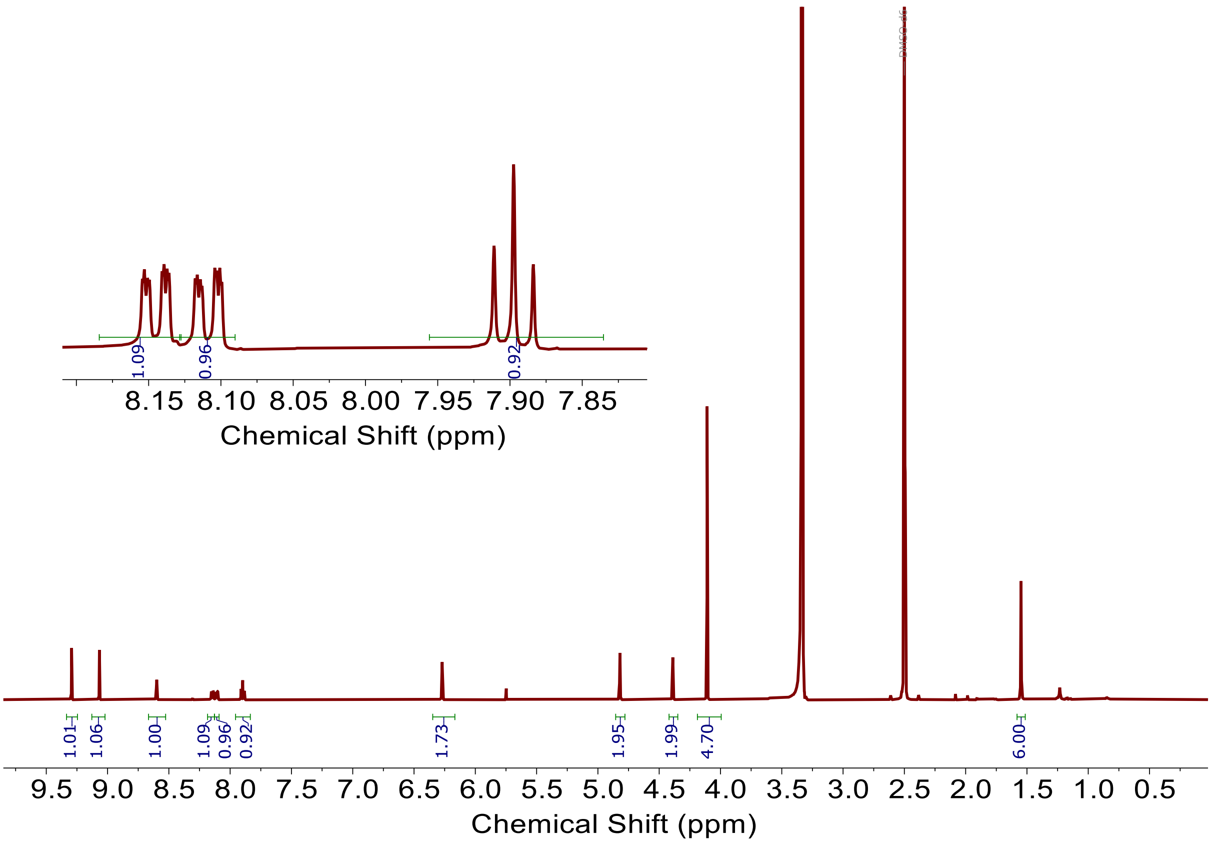

Figure S11. ^{1}H NMR spectrum of **BDP-Fc•HB.**

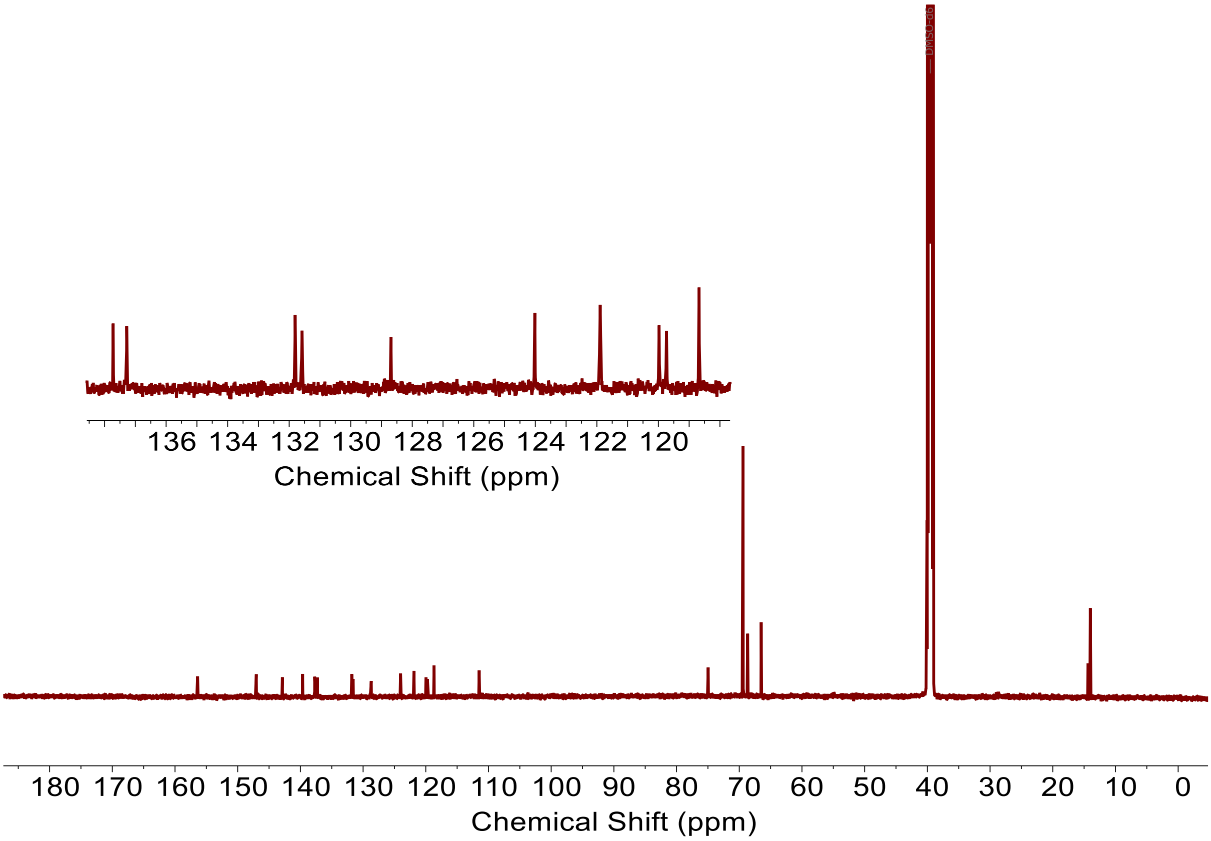

Figure S12. ^{13}C NMR spectrum of **BDP-Fc•HB.**

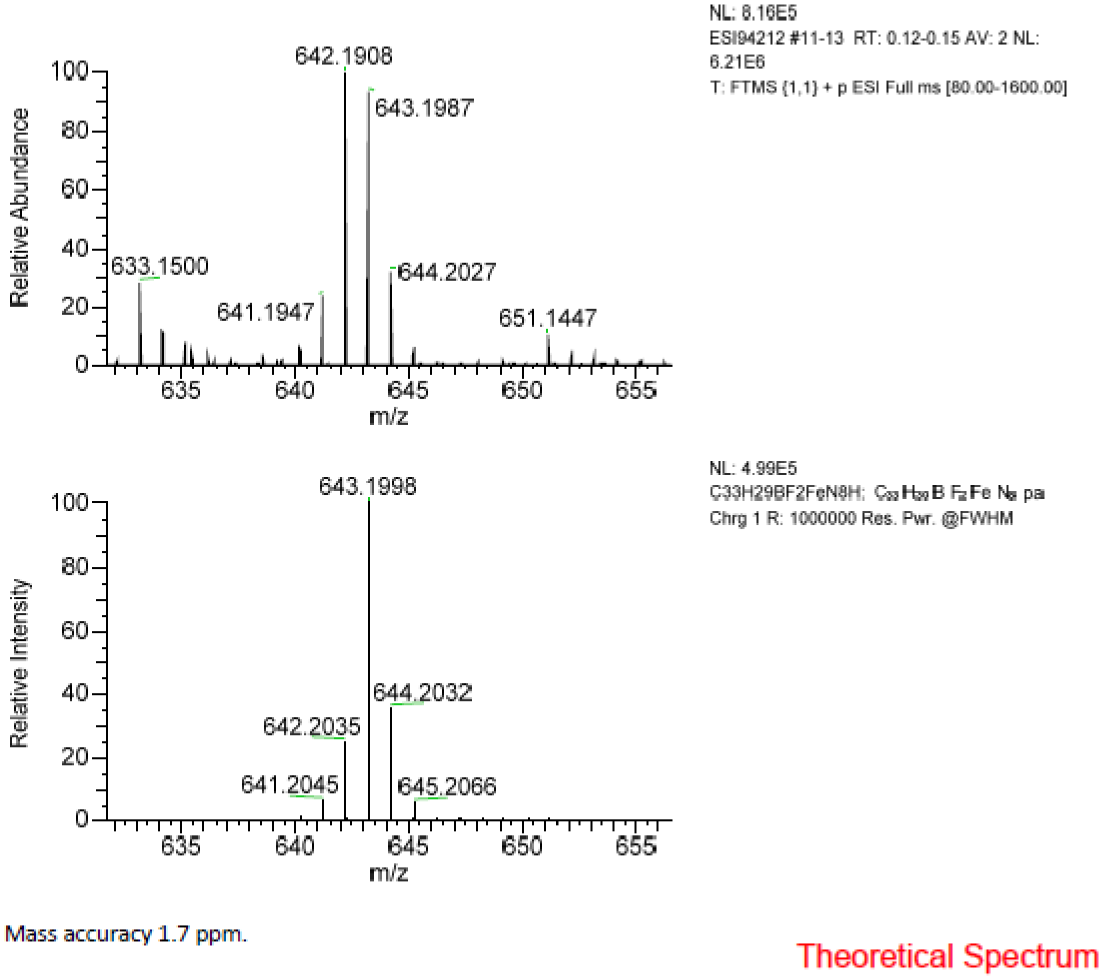

Figure S13. Experimental and theoretical mass spectra of **BDP-Fc•HB**.

BDP$_2$•HB

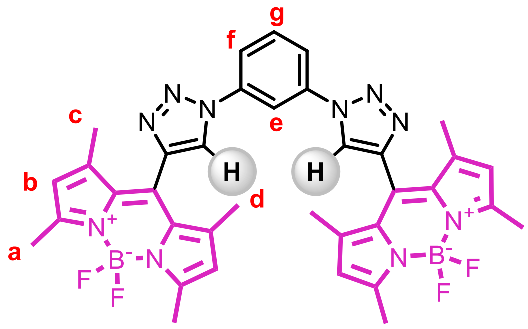

BDP$_2$•HB was prepared from **5** and 8-ethynyl-BODIPY according to General Synthetic Procedure 2, affording **BDP$_2$•HB** in 86% yield.

^{1}H NMR (600 MHz, DMSO-d_6) δ 9.29 (s, 2H, H_d), 8.69 (t, J = 2.1 Hz, 1H, H_e), 8.18 (dd, J = 8.2, 2.1 Hz, 2H, H_f), 7.93 (t, J = 8.2 Hz, 1H, H_g), 6.26 (s, 4H, H_b), 2.48 (s, 12H, H_c), 1.54 (s, 12H, H_a).

^{13}C NMR (151 MHz, DMSO-d_6) δ 156.39, 142.86, 139.64, 137.37, 132.02, 131.56, 128.62, 124.06, 121.88, 120.46, 112.10, 14.38, 13.96.

HRMS (ESI +ve) m/z: 703.3007, ([M+H]+, $C_{36}H_{33}B_2F_4N_{10}$ requires 703.3017).

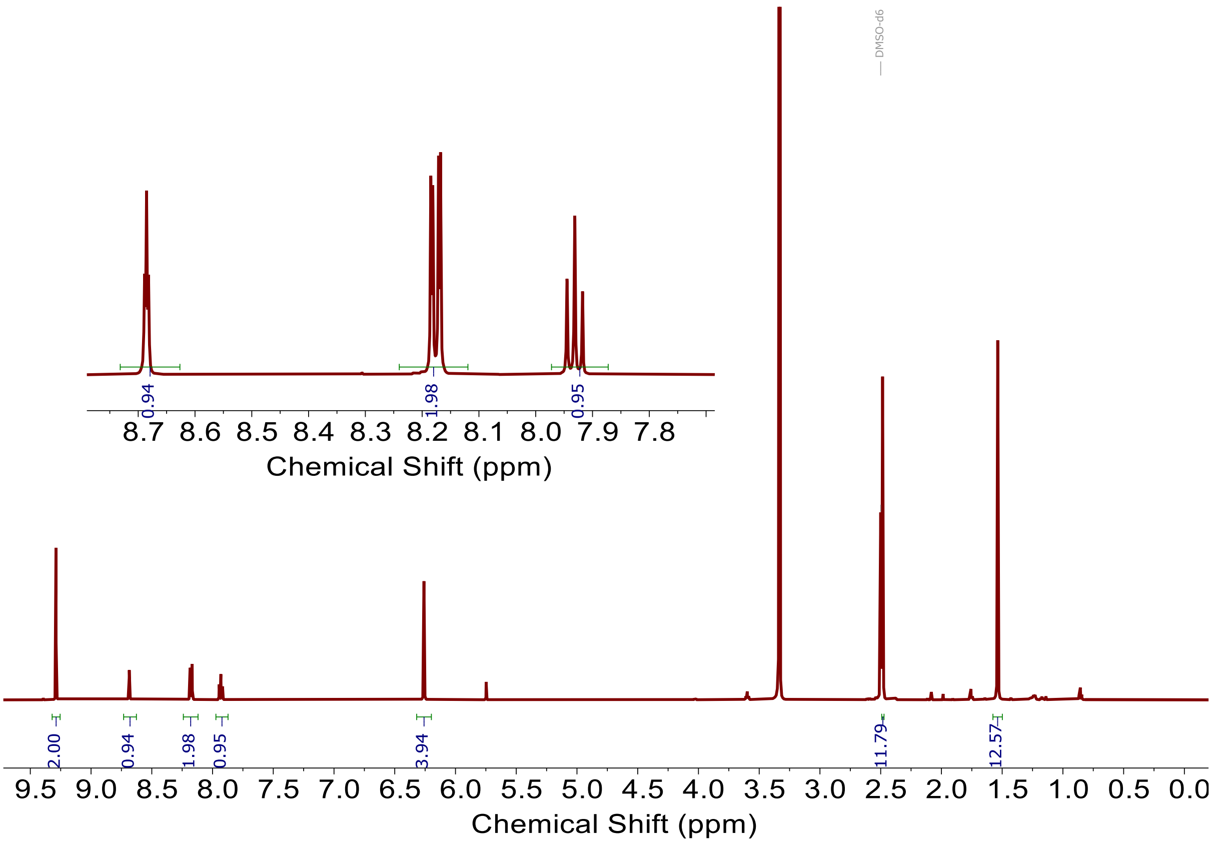

Figure S14. ^{1}H NMR spectrum of **BDP₂•HB.**

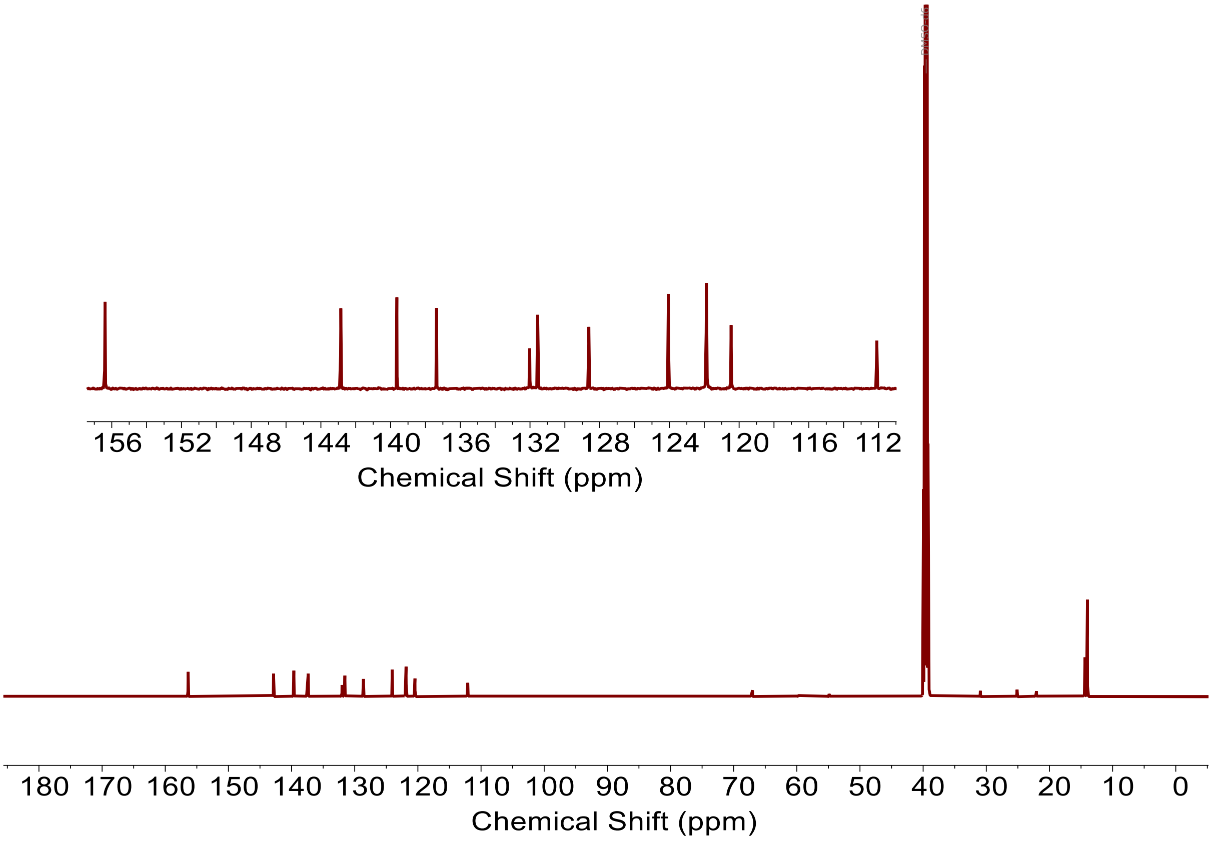

Figure S15. ^{13}C NMR spectrum of **BDP₂•HB.**

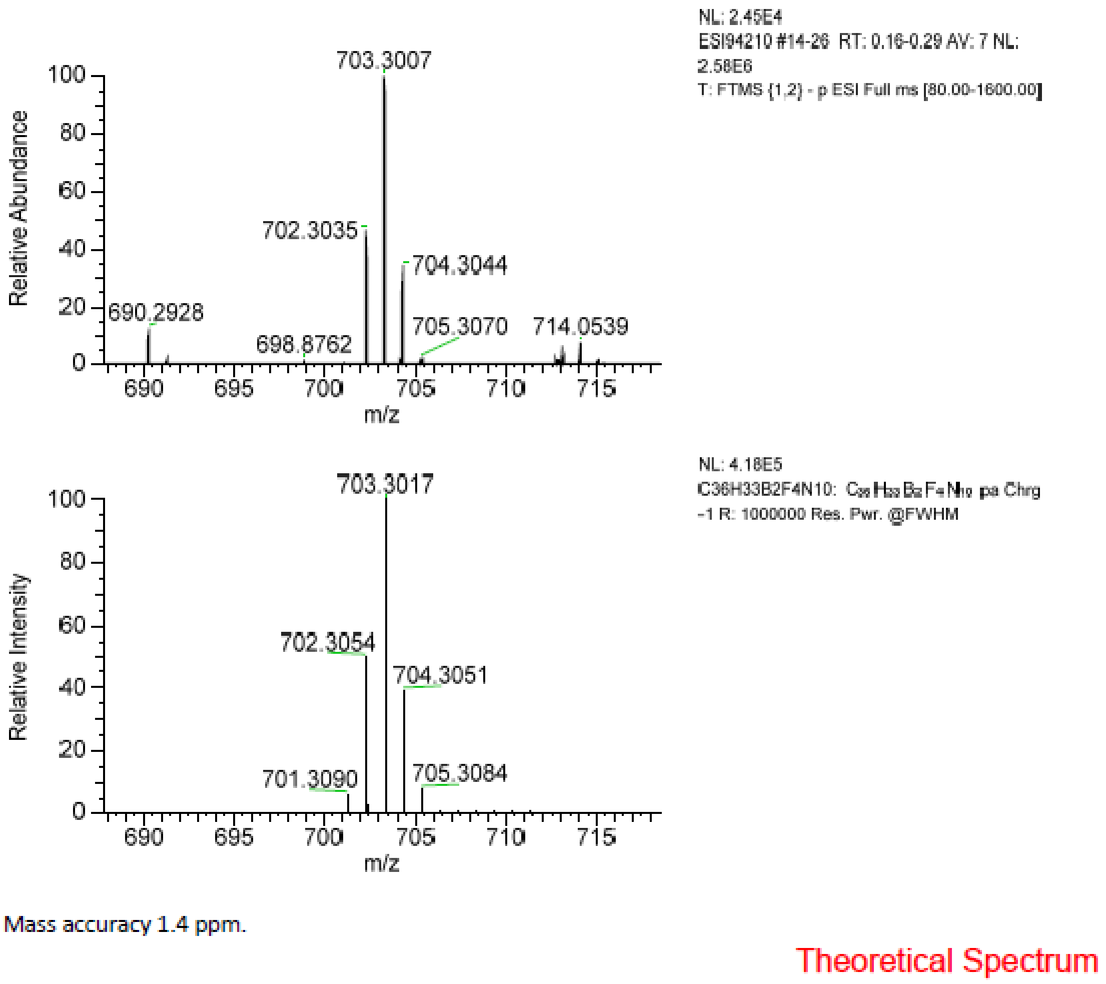

Figure S16. Experimental and theoretical mass spectra of **BDP₂•HB**.

BDP-Ph•HB

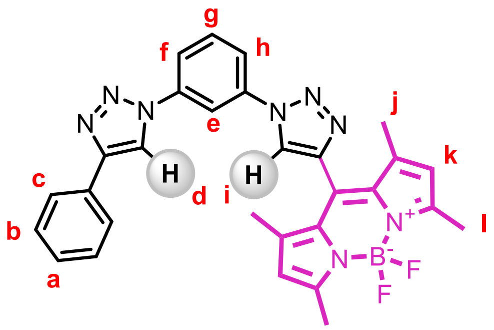

BDP-Ph•HB was prepared from **5** and phenylacetylene according to General Synthetic Procedure 2, affording **BDP-Ph•HB** in 99% yield.

^{1}H NMR (400 MHz, $CDCl_3$) δ 8.40 (t, *J* = 2.1 Hz, 1H, H_e), 8.34 (s, 1H, H_i), 8.18 (s, 1H, H_d), 8.00 – 7.85 (m, 4H, $H_{b,c}$), 7.78 (t, *J* = 8.1 Hz, 1H, H_g), 7.53 – 7.44 (m, 2H, $H_{f,h}$), 7.44 – 7.36 (m, 1H, H_a), 6.02 (s, 2H, H_k), 2.57 (s, 6H, H_j), 1.56 (s, 6H, H_l).

^{13}C NMR (151 MHz, $CDCl_3$) δ 157.26, 149.20, 142.79, 141.73, 138.52, 137.85, 132.51, 131.79, 129.87, 129.20, 128.97, 127.67, 126.10, 121.94, 121.61, 120.57, 119.94, 117.53, 112.43, 14.90, 14.65.

HRMS (ESI +ve) m/z: 557.2177, ([M+Na]+, $C_{29}H_{25}BF_2N_8Na$ requires 557.2156).

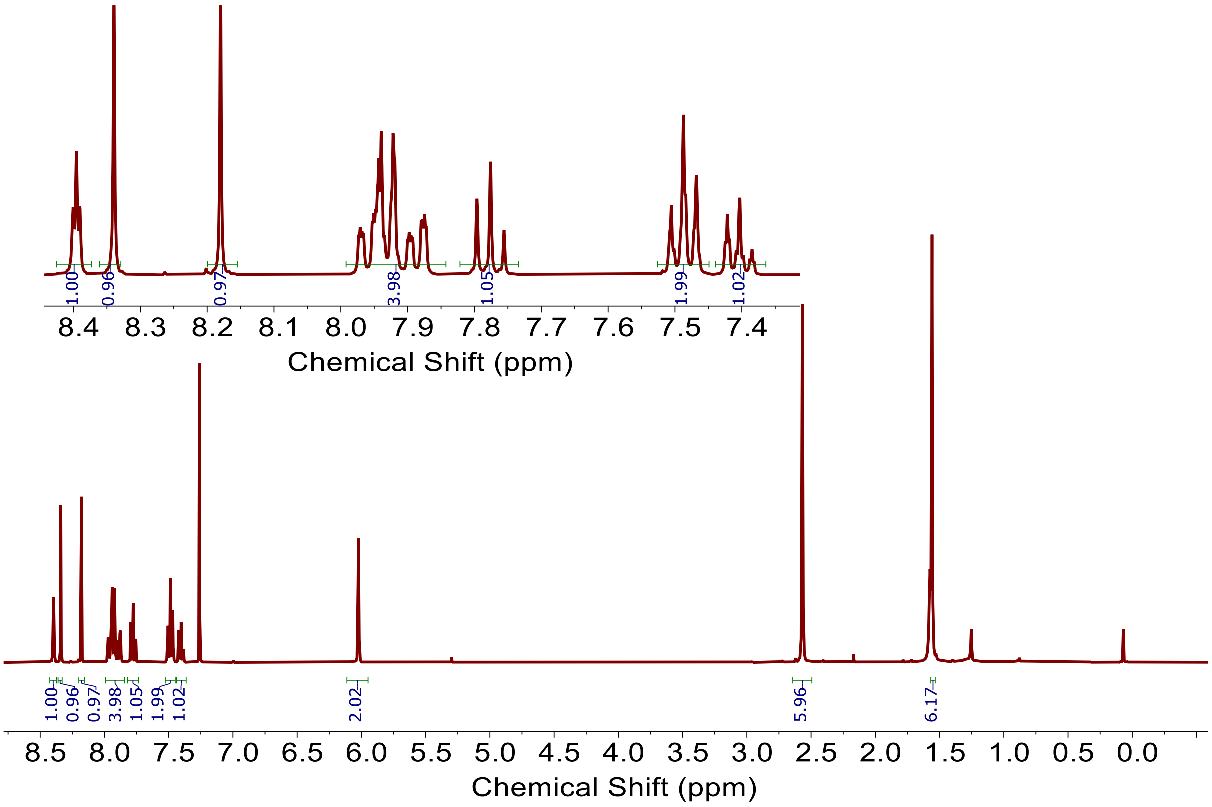

Figure S17. ^{1}H NMR spectrum of **BDP-Ph•HB.**

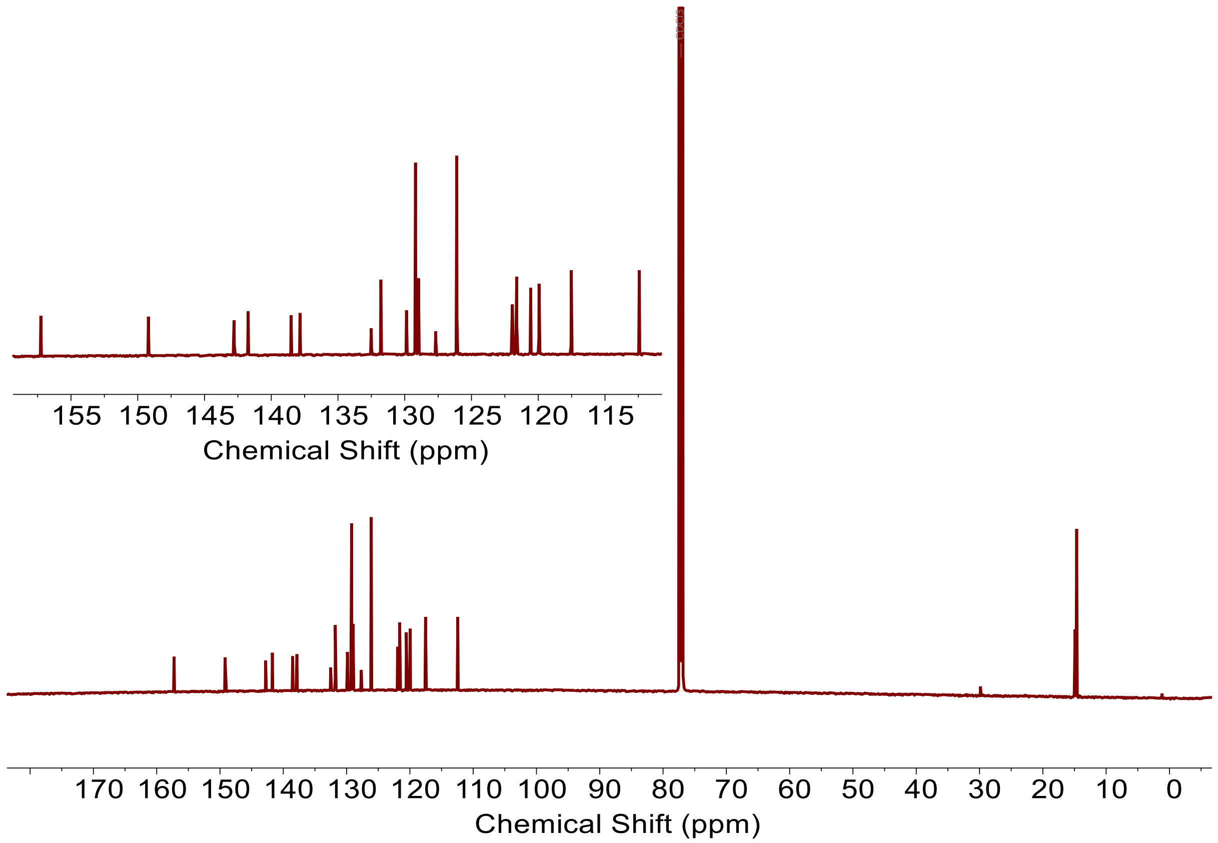

Figure S18. ^{13}C NMR spectrum of **BDP-Ph•HB.**

Expanded Spectrum RT 0.17, NL 688225, Peak [1], Target Mass 557.2156

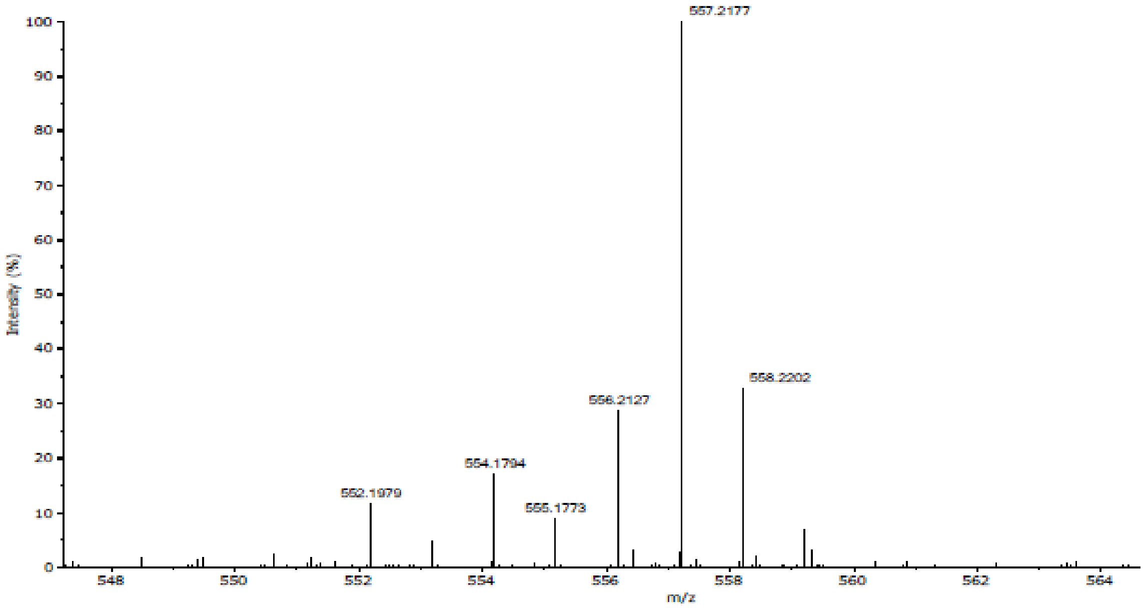

Theoretical Spectrum for C29H25BF2N8Na, Minimum Abundance 0.01%

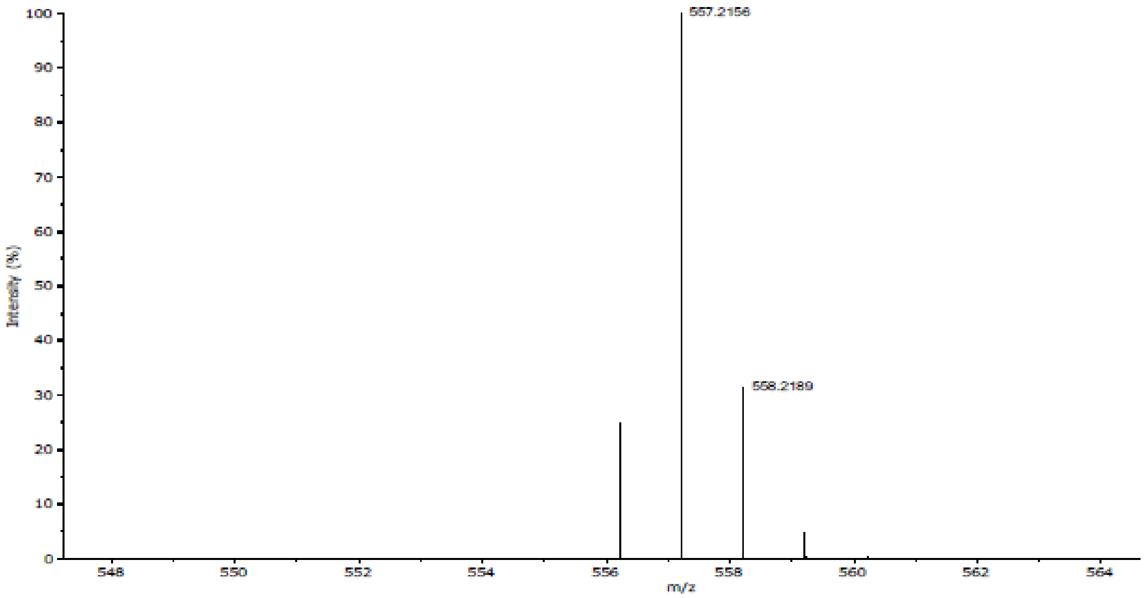

Figure S19. Experimental and theoretical mass spectra of **BDP-Ph•HB.**

BDP-Fc•XB

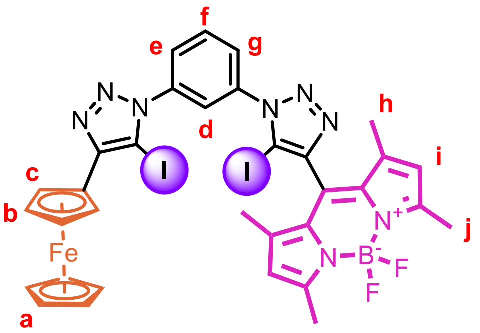

BDP-Fc•XB was prepared from **2** and **3** according to General Synthetic Procedure 2, affording **BDP-Fc•XB** in 81% yield.

^{1}H NMR (600 MHz, $CDCl_3$) δ 7.91 (t, J = 2.0 Hz, 1H, H_d), 7.89 – 7.80 (m, 3H, $H_{e,f,g}$), 6.05 (s, 2H, H_i), 5.09 (t, J = 1.9 Hz, 2H, H_c), 4.41 (t, J = 1.9 Hz, 2H, H_b), 4.20 (s, 5H, H_a), 2.59 (s, 6H, H_h), 1.54 (s, 6H, H_j).

^{13}C NMR (151 MHz, $CDCl_3$) δ 157.89, 151.58, 147.44, 142.58, 138.19, 137.51, 132.59, 130.91, 128.48, 127.45, 127.30, 124.15, 122.20, 82.84, 75.39, 74.44, 69.98, 69.52, 67.83, 15.20, 14.27.

HRMS (ESI +ve) m/z: 894.9925, ([M+H]+, $C_{33}H_{28}BF_2FeI_2N_8$ requires 894.9931).

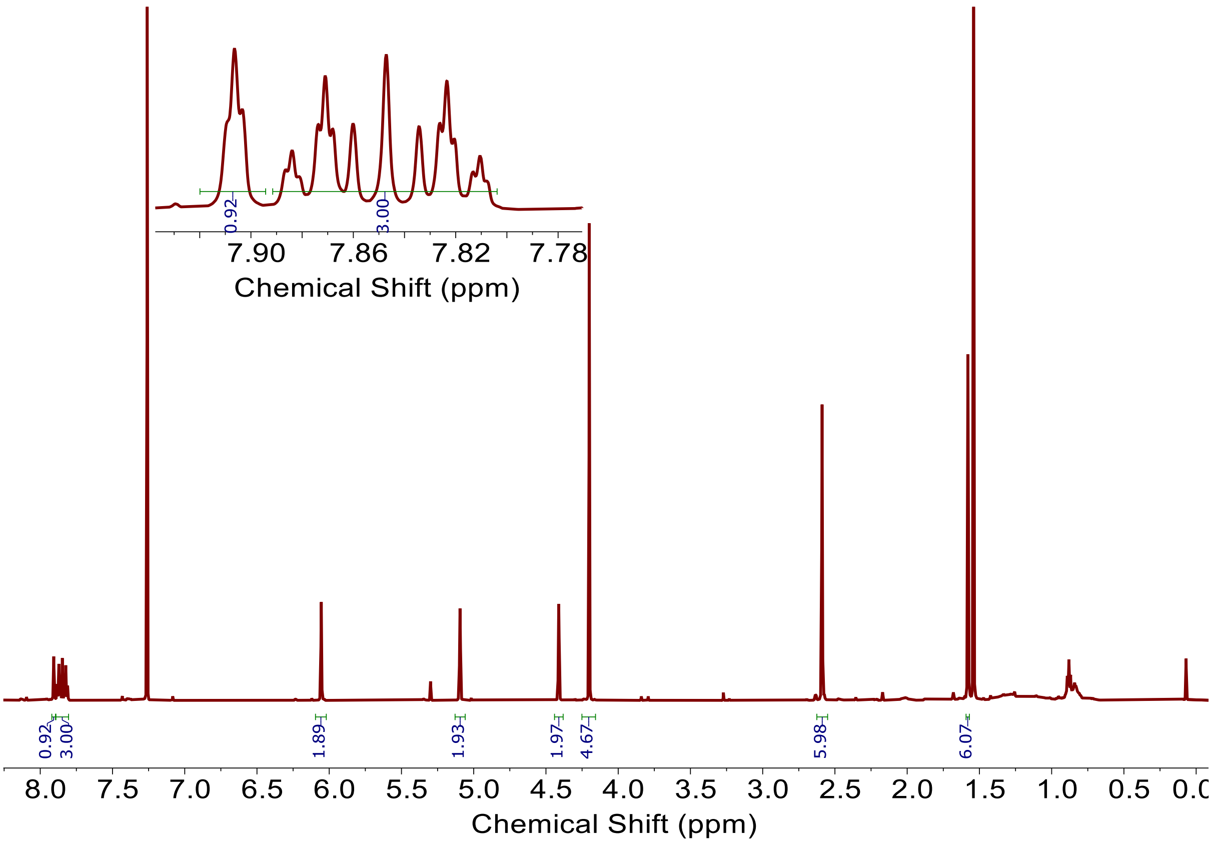

Figure S20. ^{1}H NMR spectrum of **BDP-Fc•XB.**

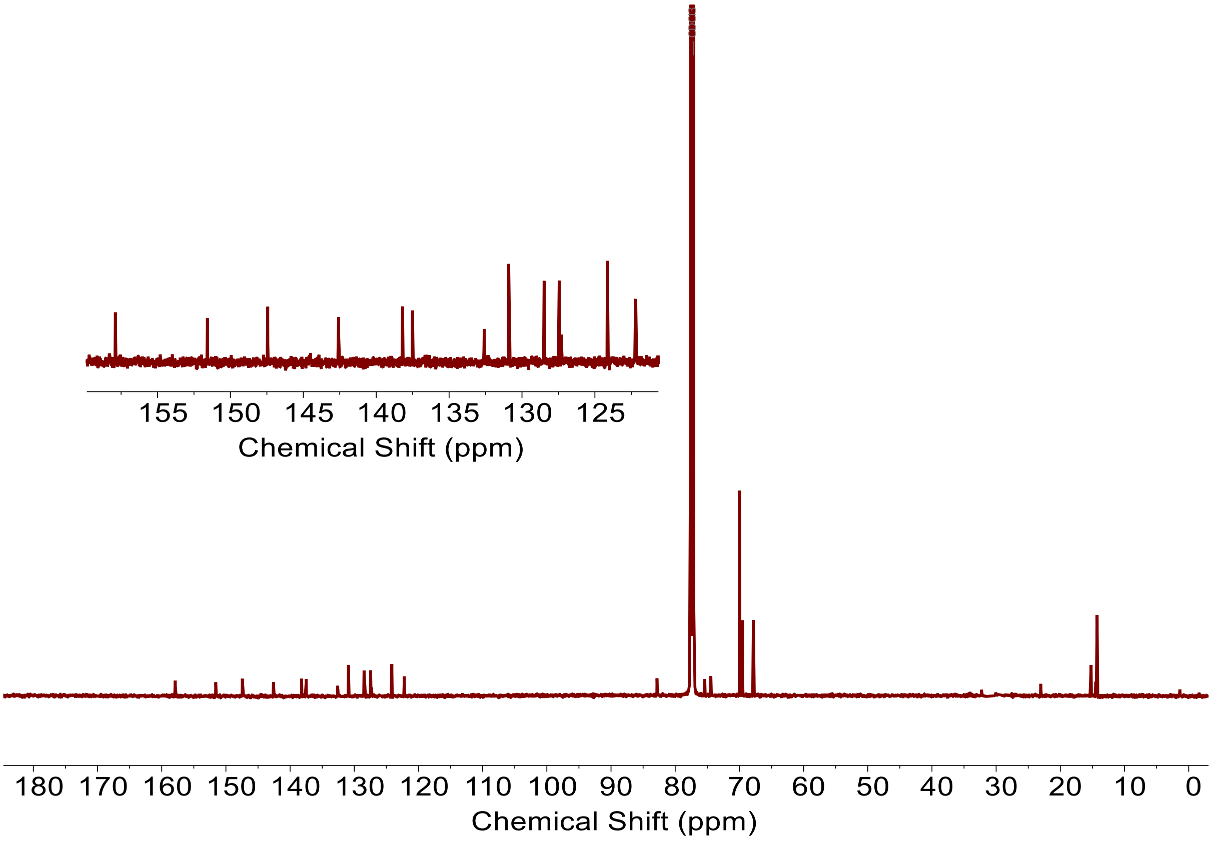

Figure S21. ^{13}C NMR spectrum of **BDP-Fc•XB.**

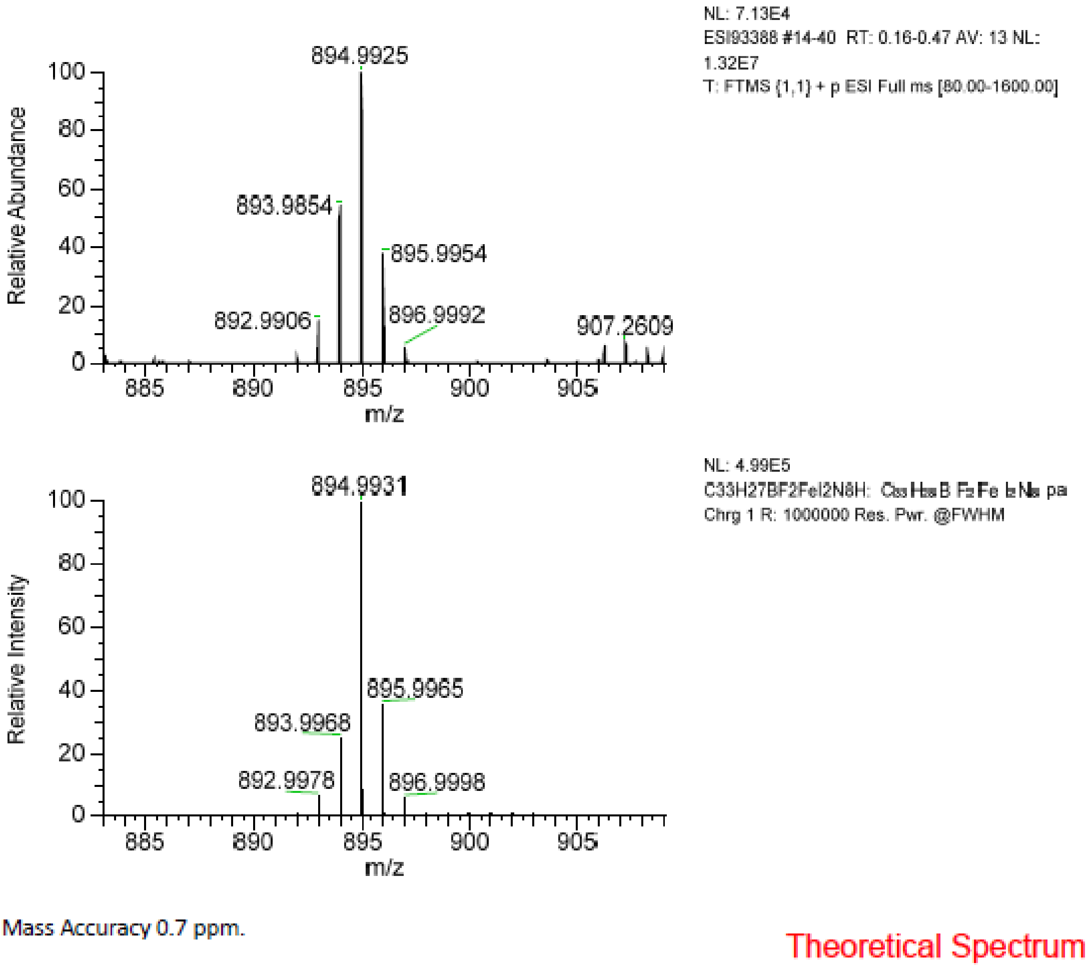

Figure S22. Experimental and theoretical mass spectra of **BDP-Fc•XB.**

BDP_2•XB

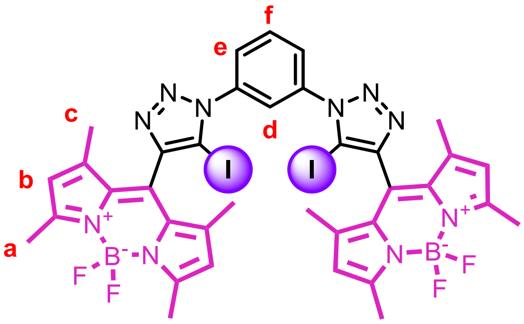

BDP_2•XB was prepared from **1** and **2** according to General Synthetic Procedure 2, affording **BDP_2•XB** in 74% yield.

^{1}H NMR (400 MHz, $CDCl_3$) δ 8.00 – 7.91 (m, 1H, H_d), 7.88 (m, 3H, $H_{e,f}$), 6.06 (s, 4H, H_b), 2.59 (s, 12H, H_c), 1.58 (s, 12H, H_a).

^{13}C NMR (151 MHz, $CDCl_3$) δ 157.79, 147.44, 142.28, 137.58, 132.35, 131.00, 127.63, 126.88, 123.24, 122.04, 82.53, 15.00, 14.08.

HRMS (ESI +ve) m/z: 957.1100, ([M+H]+, $C_{36}H_{33}B_2F_4I_2N_{10}$ requires 957.1107).

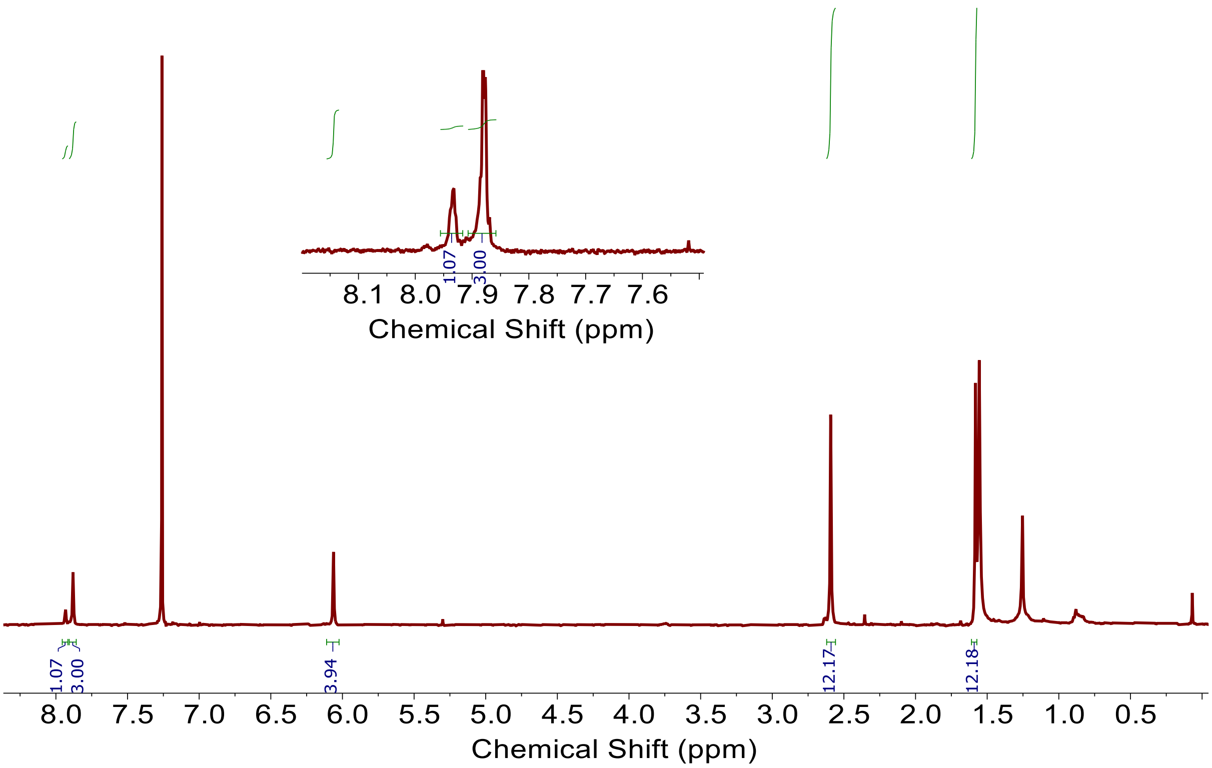

Figure S23. ^{1}H NMR spectrum of **BDP₂•XB.**

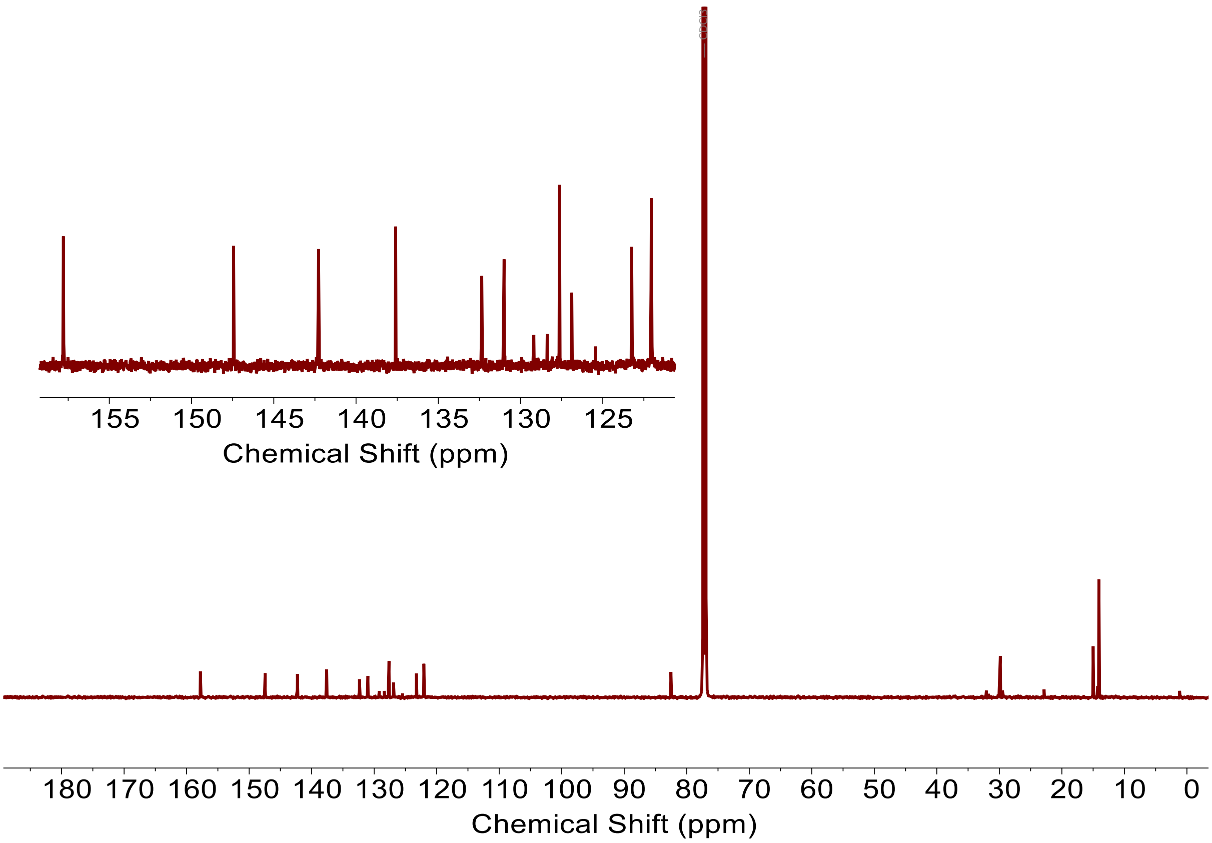

Figure S24. ^{13}C NMR spectrum of **BDP$_2$•XB.**

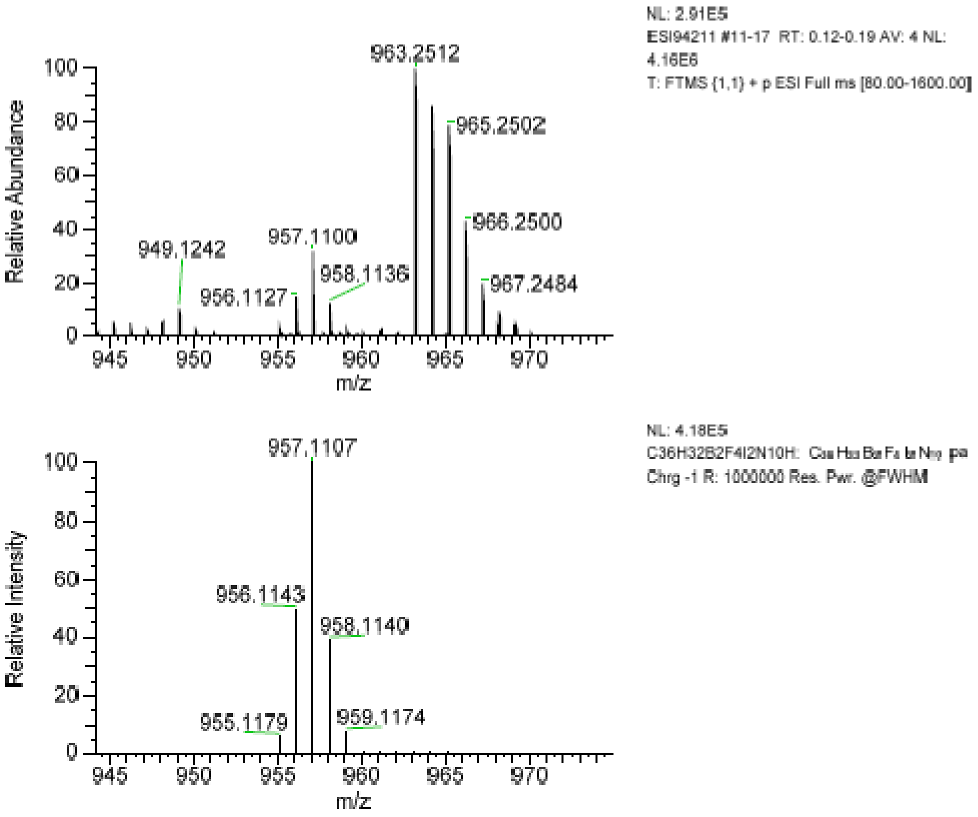

Mass accuracy 0,3 ppm.

Theoretical Spectrum

Figure S25. Experimental and theoretical mass spectra of **BDP₂ •XB.**

BDP-Ph•XB

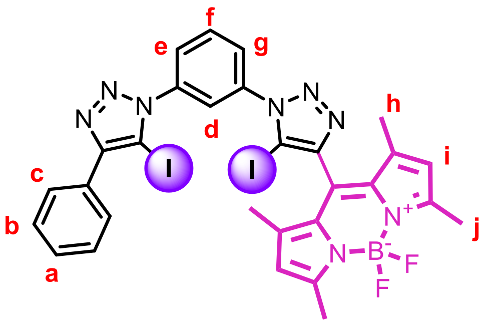

BDP-Ph•XB was prepared from **2** and **4** according to General Synthetic Procedure 2, affording **BDP-Ph•XB** in 93% yield.

^{1}H NMR (400 MHz, $CDCl_3$) δ 8.04 – 7.93 (d, 2H, H_c), 7.93 – 7.80 (m, 4H, $H_{d,e,f,g}$), 7.57 – 7.42 (m, 3H, $H_{a,b}$), 6.05 (s, 2H, H_i), 2.59 (s, 6H, H_h), 1.57 (s, 6H, H_j).

^{13}C NMR (151 MHz, $CDCl_3$) δ 157.69, 151.27, 147.23, 142.37, 138.06, 137.34, 132.37, 130.80, 129.78, 129.24, 128.87, 128.48, 127.96, 127.48, 127.06, 124.18, 122.01, 82.65, 77.57, 14.99, 14.04.

HRMS (ESI +ve) m/z: 787.0272, ([M+H]+, $C_{29}H_{24}BF_2I_2N_8$ requires 787.0273).

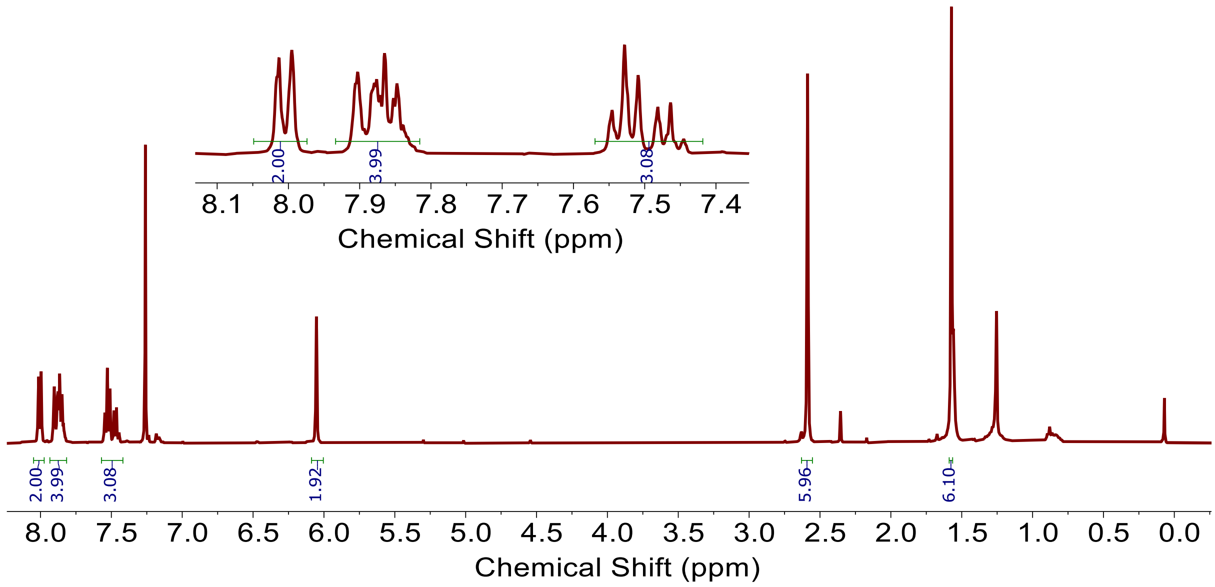

Figure S26. ^{1}H NMR spectrum of **BDP-Ph•XB.**

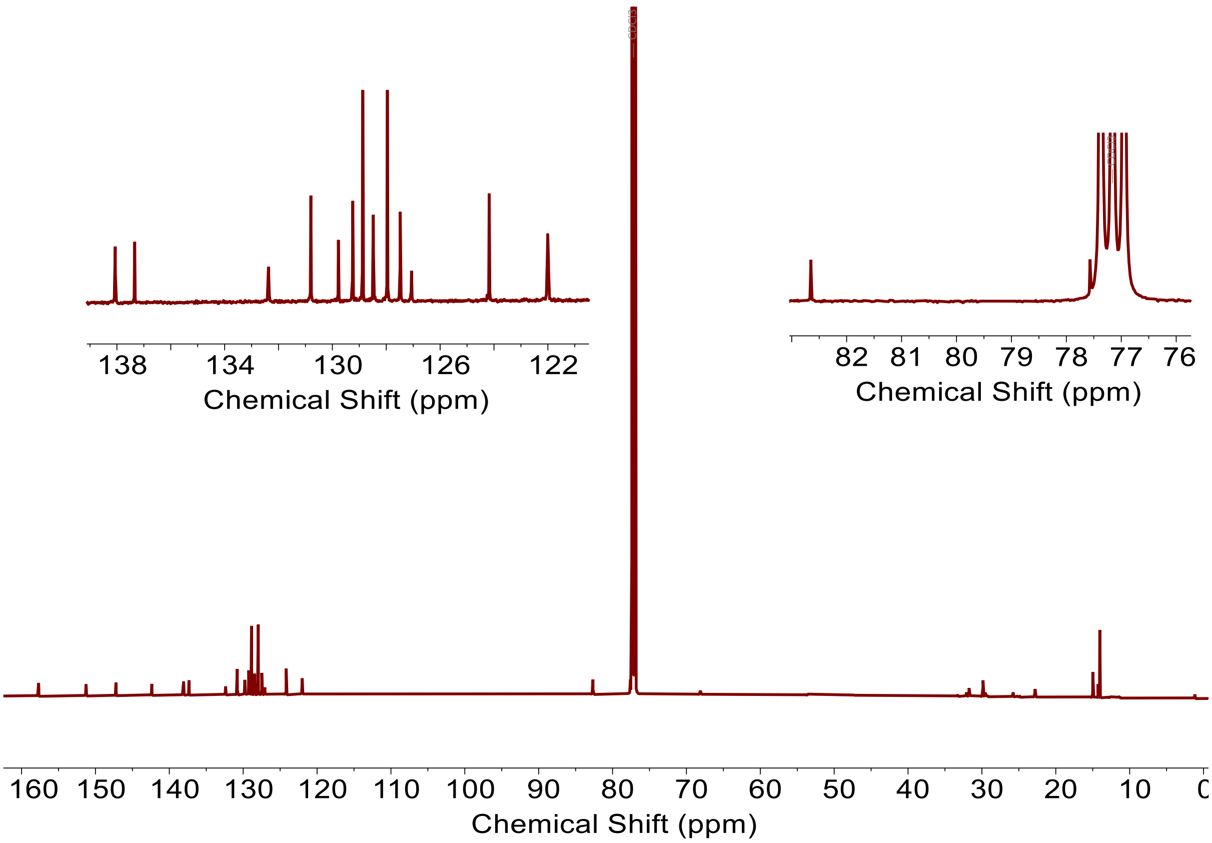

Figure S27. ^{13}C NMR spectrum of **BDP-Ph•XB.**

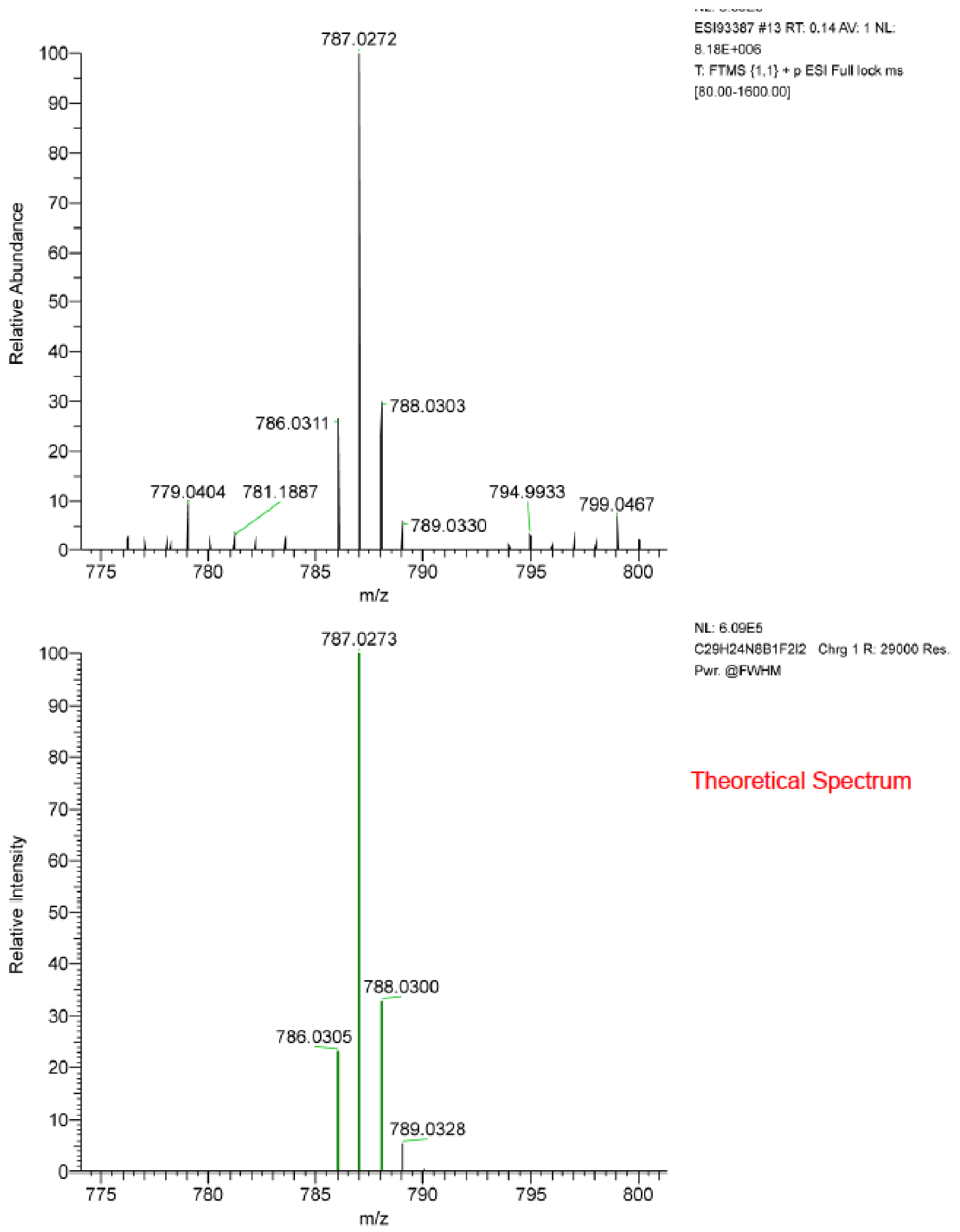

Figure S28. Experimental and theoretical mass spectra of **BDP-Ph•XB.**

3. Effect of Solvent Viscosity on BDP-Ph•HB

In order to show that the emission characteristics of the BODIPY-containing compounds were affected by the ease of rotation about the *meso* bond of the BODIPY fluorophore,[7] we measured the photophysical characteristics of **BDP-Ph•HB** in various mixtures of methanol and glycerol to investigate the effect of increasing solvent viscosity. As the volume fraction of glycerol increased, the emission intensity increased (Figure S29). This was ascribed to a decrease in the degree of rotation about the *meso* bond of the fluorophore and indicates that rotation about this bond is a significant decay pathway for the BODIPY excited state.

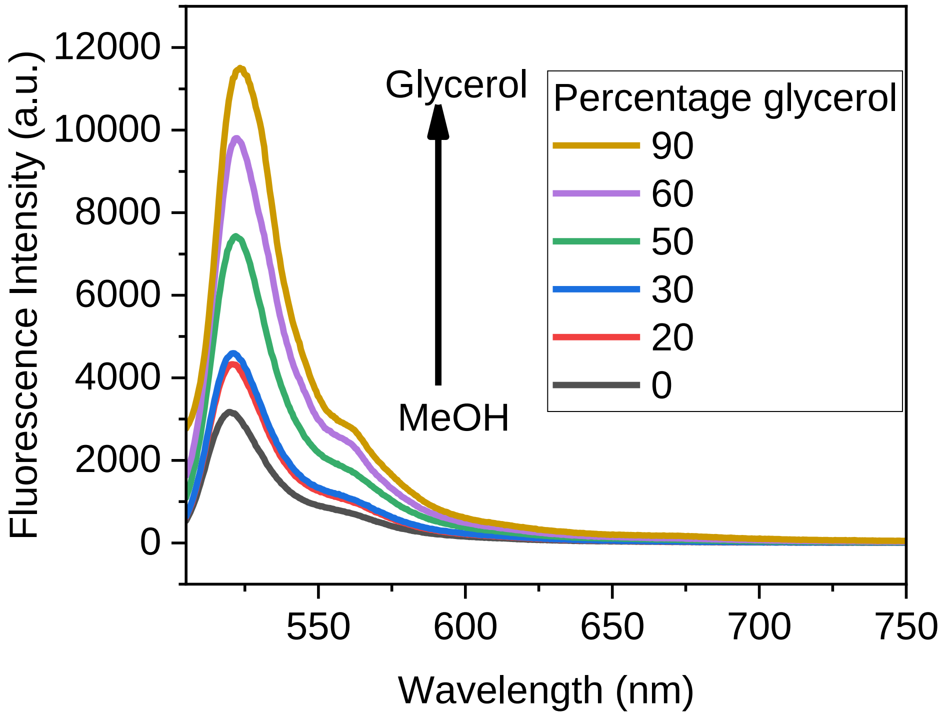

Figure S29. Fluorescence spectra of 1 µM **BDP-Ph•HB** in MeOH:glycerol mixtures.

By integrating the fluorescence intensity between 500 and 650 nm and constructing the plot shown in Figure S30, a viscosity sensitivity constant of 0.22 was determined for **BDP-Ph•HB**, which is smaller than but consistent with BODIPY-based molecular rotors which have more bulky groups in the *meso* position reported previously.[7-8]

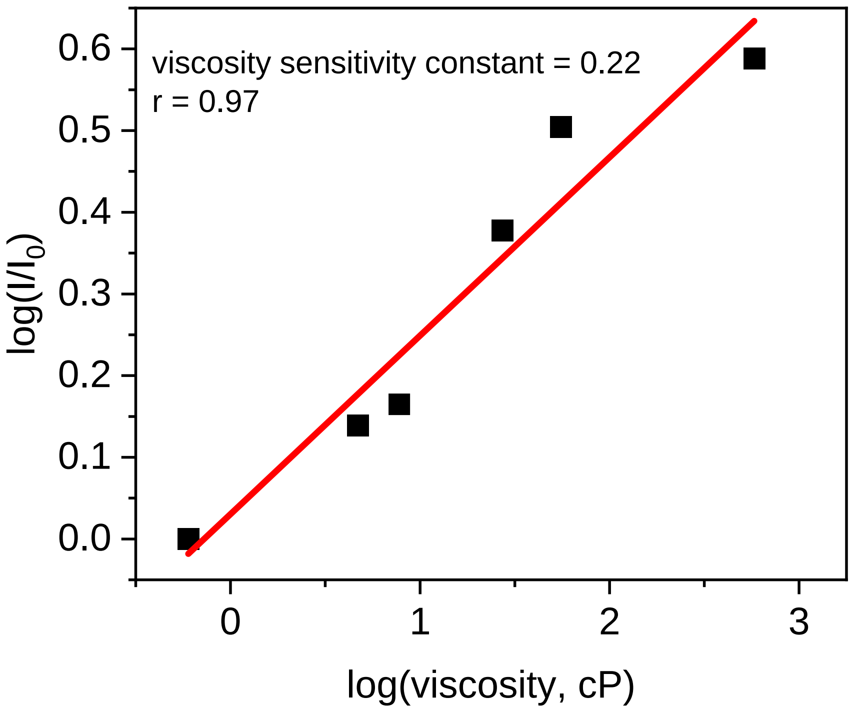

Figure S30. Linear dependency of the log(integrated fluorescence intensity) versus log(viscosity) for **BDP-Ph•HB** in MeOH:glycerol mixtures.

4. ^{1}H NMR Titration of BDP-Fc•HB with TBABr

In order to corroborate the hypothesis that the anion binding affinity of **BDP-Fc•HB** is in a similar range to **BDP-Ph•HB**, we conducted a ^{1}H NMR titration of **BDP-Fc•HB** with TBABr in acetone-d_6.

The ^{1}H NMR titration experiment was conducted on a Bruker AVIII 500 spectrometer at 298K. The host sample was prepared in 0.5 mL volume and at 1.0 mM concentration. A solution of TBABr (100 mM) was added to the host sample as aliquots in increments of 0.0, 0.4, 0.8, 1.2, 1.6, 2.0, 2.4, 2.8, 3.2, 3.6, 4.0, 5.0, 6.0, 8.0, 10.0, 14.0 and 20.0 equivalents. The sample was thoroughly shaken after the addition of each aliquot before recording its spectrum. The bound and unbound species were found to be in fast exchange on the NMR timescale and perturbations were observed in the signals arising from protons *d*, *e* and *i* (Figure S31). Fitting of the binding isotherms (Figure S32) to a 1:1 host : guest binding model afforded a binding constant of 152 ± 1 M^{-1}, which is comparable to the Br^- binding constant for **BDP-Ph•HB** determined *via* fluorescence titration.

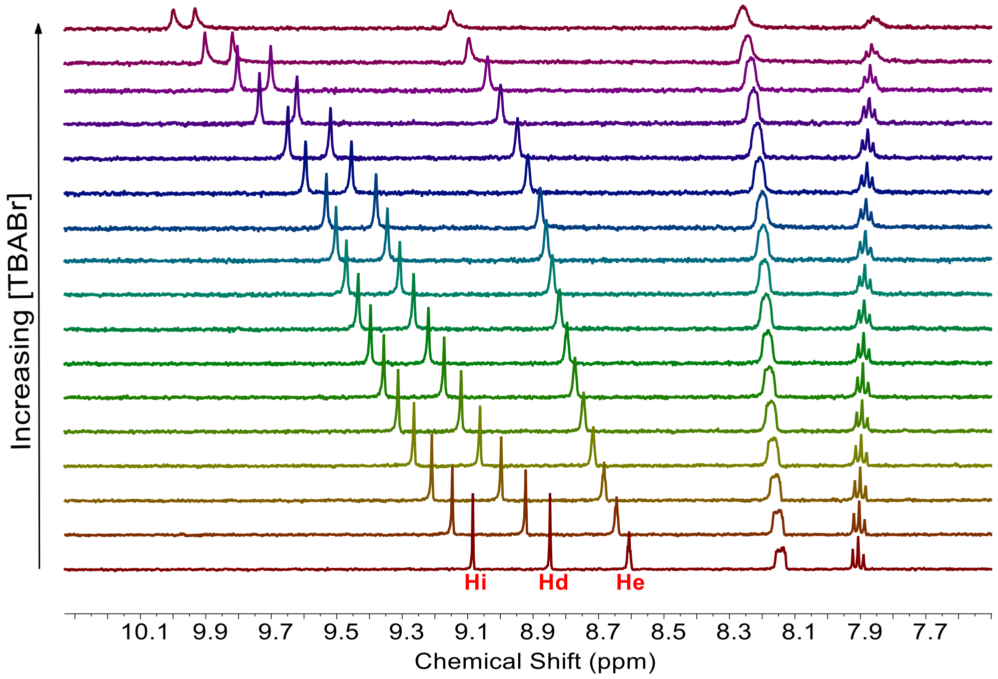

Figure S31. Stacked, truncated ^{1}H NMR spectra of **BDP-Fc•HB** in the presence of increasing equivalents of TBABr in acetone-d_6.

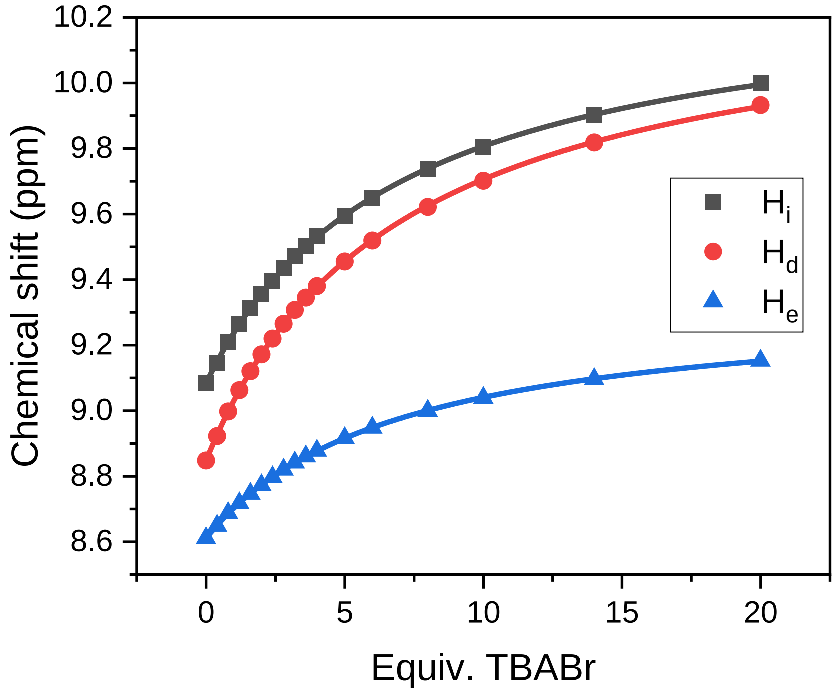

Figure S32. Plot of the chemical shift of protons proximal to the binding cavity of **BDP-Fc•HB** as a function of increasing equivalents of TBABr in acetone-d_6. Solid lines represent fits according to 1:1 host-guest stoichiometric binding.

5. Fluorescence Lifetime Measurements

Fluorescence Lifetimes and Mechanism of Fluorescence Modulation

The fluorescence lifetime τ_f is given by

$$\tau_f = \frac{1}{k_f + k'} \qquad \text{(eqn. S2)}$$

where k_f is the rate of emission from the S_1 state and k' is the combined rate of all other, non-radiative decay processes from the S_1 state. The fluorescence quantum yield Φ_f (which is proportional to fluorescence intensity) is given by

$$\phi_f = k_f \tau_f = \frac{k_f}{k_f + k'} \qquad \text{(eqn. S3)}$$

For any given receptor, τ_f and Φ_f appear to be affected by the same factor A upon bromide binding, namely:

$$\phi_{f,0} = A\phi_{f,Br} \qquad \text{(eqn. S4)}$$

and

$$\tau_{f,0} = A\tau_{f,Br} \qquad \text{(eqn. S5)}$$

where the subscripts *0* and *Br* indicate the absence and presence of 3 mM bromide respectively. (This factor A is not the same across all receptors, but for each receptor, τ_f and Φ_f appear to be affected by the same factor.)

By division, we obtain

$$\frac{\phi_{f,0}}{\tau_{f,0}} = \frac{\phi_{f,Br}}{\tau_{f,Br}} \qquad \text{(eqn. S6)}$$

inserting the relation in equation S3, we obtain

$$k_{f,0} = k_{f,Br} \qquad \text{(eqn. S7)}$$

This result shows that if fluorescence intensity and fluorescence lifetime are affected by the same factor, then these changes must be driven by changes in k', not k_f. Consider the counterfactual: if the increase in fluorescence intensity upon anion binding for **BDP_2•XB** was driven by an increase in k_f, we would expect a concomitant decrease in the fluorescence lifetime, but an increase in lifetime is observed.

As described in the main text, from the fluorescence lifetimes the rate of electron transfer k_{ET} can be calculated[9] by comparison of the Fc-BDP hosts to the “reference” BDP-Ph via:

$$k_{ET} = \frac{1}{\tau_{BDPFc}} - \frac{1}{\tau_{BDPPh}} = 1.66 \times 10^9\ s^{-1} \qquad \text{(eqn. 1)}$$

as exemplified above for the XB system. For the HB receptors this rate is in the same range at $1.88 \times 10^9\ s^{-1}$, however note that the errors associated with lifetime measurements below 1 ns are significantly larger. These values are in the same range as similar known systems.[10] In the presence of bromide the rate of electron transfer expectedly increases for **BDP-Fc•XB**, consistent with more efficient PET quenching:

$$k_{ET,\ Br^-} = \frac{1}{\tau_{BDPFc}} - \frac{1}{\tau_{BDPPh}} = 3.77 \times 10^9\ s^{-1}$$

For the HB system the k_{ET} in the presence of bromide also increases to $4.47 \times 10^9\ s^{-1}$.

We can further calculate the PET efficiency θ via:

$$\theta_{ET,\ XB} = 1 - \frac{\tau_{BDPFc}}{\tau_{BDPPh}} = 0.68 \qquad \text{(eqn. 2)}$$

And in the presence of bromide:

$$\theta_{ET,XB,Br} = 1 - \frac{\tau_{BDPFc}}{\tau_{BDPPh}} = 0.90$$

Similarly, the PET efficiency also increases for the HB system from 0.68 (free host) to 0.80 (in the presence of bromide). Note that the similarly high k_{ET} and θ_{ET} of the HB system in comparison to XB hosts is not in contradiction with the lack of fluorescence (quenching) response of **BDP-Fc•HB** towards anions (Figure 4). This is because these calculations/observations are always relative to the BDP-Ph system, in which fluorescence turn-on due to rigidification is observed. This is to say that in the XB system the anion binding-induced PET quenching enhancement can outcompete the fluorescence turn-on arising from receptor rigidification, while in the HB system the latter is larger and cancels any PET effects such that for **BDP-Fc•HB** no significant anion response is observed.

6. Voltammetric Characterisation of BDP-Fc•XB/HB

Voltammetric characterisation of the BDP-Fc receptors was first carried out in anhydrous, degassed ACN in the presence of 100 mM $TBAPF_6$. This solvent system was chosen as it has a much larger solvent window than acetone and thus enables characterisation of both Fc and BODIPY couples.

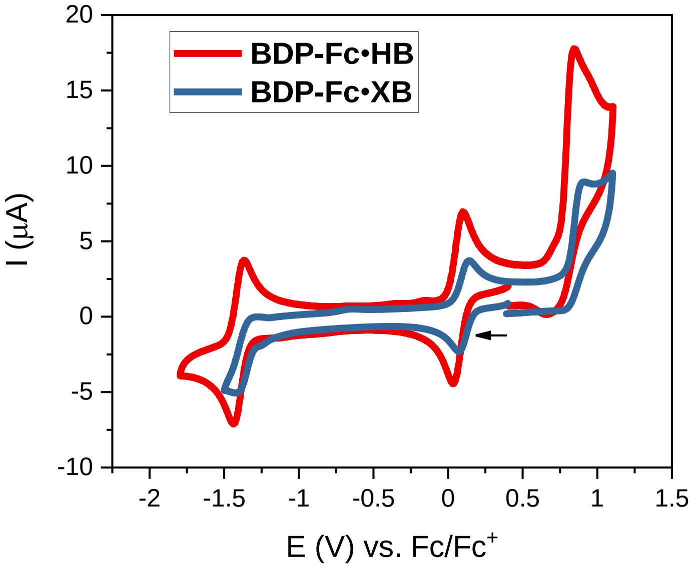

Figure S33. CVs of 0.5 mM **BDP-Fc•XB/HB** in anhydrous, degassed ACN, 100 mM $TBAPF_6$, at a scan rate of 0.1 V/s. The black arrow indicates the initial direction and start point of the scan.

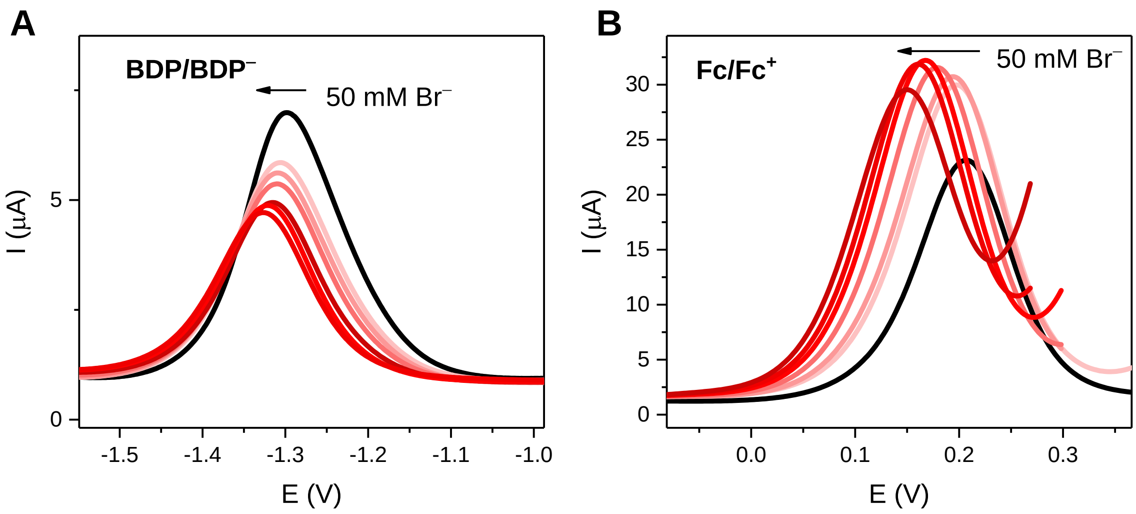

Figure S34. SWVs of 0.5 mM **BDP-Fc•XB** in anhydrous, degassed ACN, 100 mM $TBAPF_6$, upon titration with Br^-. A) Reductive BDP couple. B) Oxidative Fc couple. The overall ionic strength was kept constant at 100 mM throughout.

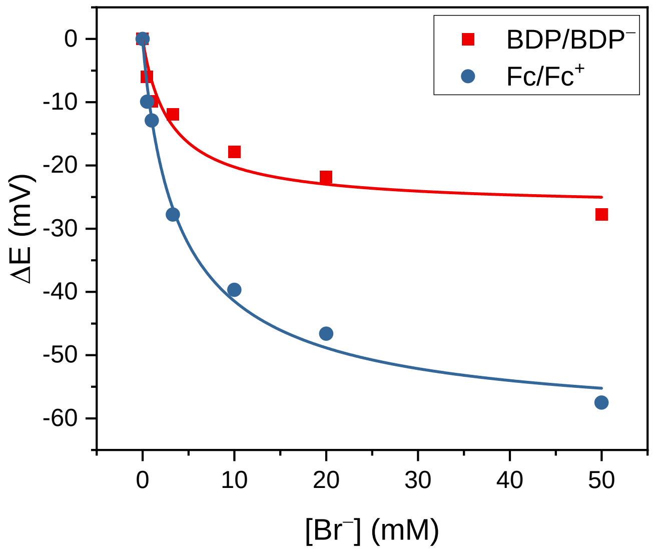

Figure S35. Cathodic voltammetric shift of the BDP/BDP⁻ (red) and Fc/Fc⁺ (blue) redox couples of 0.5 mM **Fc-BDP•XB** in in degassed, anhydrous ACN, 100 mM $TBAPF_6$ upon titration with Br^-. Lines represent fits according to 1:1 host-guest stoichiometric binding model.

As briefly discussed in the main text, the Fc/Fc⁺ redox couple of both receptors displayed a well-defined, reversible single-electron wave, with a slightly more anodic $E_{1/2}$ for the XB sensor (Figure S36).

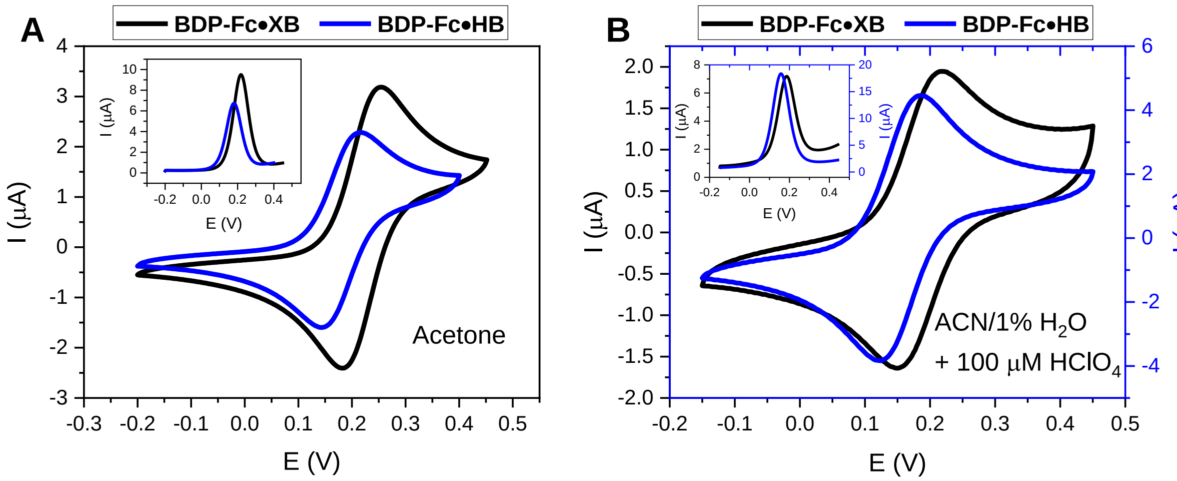

Figure S36. Cyclic voltammograms and square wave voltammograms (see insets) of 0.25 mM **BDP-Fc•XB/HB** in A) acetone and B) ACN/1% H_2O, 100 µM $HClO_4$ (both with 100 mM $TBAClO_4$ as supporting electrolyte). Potentials wrt. Ag|$AgNO_3$.

7. Gibbs Free Energy of Electron Transfer

As described in the main text, the Gibbs free energy of electron transfer ΔG_{ET}, which can be obtained via:[9, 11]

$$\Delta G_{ET} = e\left[E_{Ox,Don} - E_{Red,Acc}\right] - E_{00} - \Delta \mathrm{G_S} \qquad \text{(eqn. 3)}$$

Where, E_{Ox} and E_{Red} are the half-wave potentials of the donor (Fc) and acceptor (BDP), respectively, E_{00} the photoexcitation energy for the BODIPY (also called the vibrational zero electronic energy of the excited partner) and $\Delta \mathrm{G_S}$ is the coulombic energy of the charge separated state.

The photoexcitation energy E_{00} of the BODIPY can, in the case of mirror image spectra (as is the case here), be simply calculated from the midpoint between absorption and emission maxima via $E_{00} = \frac{h * c}{\lambda_{00}}$.[12]

For XB and HB the midpoint between these spectra in acetone is 516.5 and 514 nm, respectively, which gives E_{00} of 2.40 and 2.41 eV, for **BDP-Fc•XB** and **BDP-Fc•HB** respectively.

From this, and the electrochemically determined potentials, ΔG_{ET} can be obtained in good approximation (without a consideration of $\Delta \mathrm{G_S}$, vide infra):

$$\Delta G_{ET,XB} = e[0.097V - -1.400V] - 2.4\ eV - \Delta \mathrm{G_S} = \mathbf{-0.903\ eV} - \Delta \mathrm{G_S}$$

$$\Delta G_{ET,HB} = e[0.073V - -1.402V] - 2.41\ eV - \Delta \mathrm{G_S} = \mathbf{-0.935\ eV} - \Delta \mathrm{G_S}$$

These values confirm that in both cases ET is thermodynamically exergonic and thus favourable.

$\Delta \mathrm{G_S}$, the coulombic energy of the charge separated state, can, for singly charged donor/acceptor (after ET) be described by:

$$\Delta G_S = -\frac{e^2}{4\pi\varepsilon\varepsilon_0 R_{DA}} - \frac{e^2}{8\pi\varepsilon_0}\left(\frac{1}{r_D} + \frac{1}{r_A}\right)\left(\frac{1}{\varepsilon_{EC}} - \frac{1}{\varepsilon}\right) \text{ (eqn. S8)}$$

where ε_{EC} is the dielectric constant of the solvent utilised for electrochemical experiments (here acetonitrile), ε the dielectric constant of the photophysical system, R_{DA} the center-to-

center distance between donor and acceptor and r_D and r_A the radii of donor and acceptor. The latter parameters are not directly available, such that ΔG_S can only be estimated:

Approximation of the first term of eqn. S8, with $\varepsilon = 21$ for acetone and R_{DA} = 0.5 or 2 nm:

$$\Delta G_S = -\frac{e^2}{4\pi\varepsilon\varepsilon_0 R_{DA}} = -1.44 \times 10^{-9} eVm \times \frac{1}{\varepsilon R_{DA}}$$

For R_{DA} = 0.5 nm:

$$\Delta G_S = -0.137\ eV$$

For R_{DA} = 2 nm:

$$\Delta G_S = -0.034\ eV$$

Which in both cases is much smaller than the above-stated values for ΔG_{ET} .

The second term of the ΔG_S equation can also be estimated using literature values of R_d = 0.175 nm and R_a = 0.434 nm as approximate radii of donor and acceptor, and $\varepsilon = 37.5$ for ACN.[11a]

$$\Delta G_S = -\frac{e^2}{4\pi\varepsilon\varepsilon_0 R_{DA}} - \frac{e^2}{8\pi\varepsilon_0}\left(\frac{1}{r_D} + \frac{1}{r_A}\right)\left(\frac{1}{\varepsilon_{EC}} - \frac{1}{\varepsilon}\right) = -\frac{e^2}{4\pi\varepsilon\varepsilon_0 R_{DA}} + 0.121 eV$$

This value is again much smaller than the first term of ΔG_{ET} (≈ –0.9 eV) and is also of opposite sign as the first term of ΔG_S, further reducing the influence of ΔG_S on the overall value of ΔG_{ET} .

As shown in Figures S34-35, titration of **BDP-Fc•XB** with Br^- in acetonitrile induced cathodic voltammetric shifts of both the oxidative Fc couple and the reductive BODIPY couple, whereby the shift in the latter was significantly smaller. In the presence of an increasing concentration of Br^- the *difference* in redox potentials between the ferrocene donor and BDP acceptor thus *decreased* (up to ≈30 mV decrease in $E_{Ox,Don} - E_{Red,Acc}$). This is in turn reflected in an *increase* in the thermodynamic driving force for PET, i.e. a lower ΔG_{ET} (eqn. 3).

Specifically, ΔG_{ET} became more negative: from –0.903 eV in the absence of anion to –0.933 eV in the presence of excess bromide. This indicates that anion binding can thermodynamically favor PET and, at least partially, explain the fluorescence turn-OFF response of **BDP-Fc•XB** to anions. It is important to note that the voltammetric measurements that underpin these calculations were for practicality reasons conducted in acetonitrile, a significantly more polar and competitive solvent than acetone (used for the optical studies). The magnitude of the cathodic voltammetric shift of the redox couples (in particular that of the oxidative Fc couple) is expected to be even larger in the less competitive acetone,[2a, 13] such that the increase in thermodynamic driving force for PET is most likely even larger in acetone.

8. Voltammetric Titrations

The redox-sensing properties of **BDP-Fc•XB/HB** were first probed in acetone, wherein significant cathodic voltammetric perturbations of the Fc/Fc^+ redox couple were observed in the presence of Cl^-, Br^- or HSO_4^- (Figure S37), which is in good agreement with numerous reports on related systems.[2a, 13-14]†

At low anion concentrations in acetone the **BDP-Fc•XB** receptor displayed larger cathodic shift magnitudes towards the halide anions in comparison to **BDP-Fc•HB** (Figure 8), with the maximum response observed in the presence of chloride (ΔE_{Max} = –64 mV, Table S1) and a notably flatter response isotherm in the presence of bisulfate. In contrast, the HB congener displayed a preference for this latter oxoanion with ΔE of up to –73 mV, once again underscoring an inherent XB-halide/HB-oxoanion preference.[2a, 13, 15]

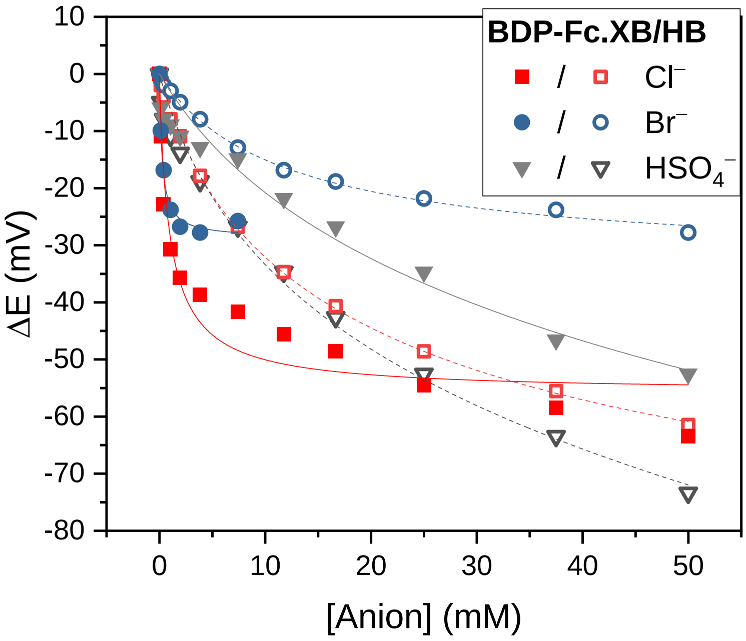

Figure S37. Cathodic voltammetric shifts of the Fc/Fc$^+$ redox couple of 0.1 mM **BDP-Fc•XB** (filled symbols) and **BDP-Fc•HB** (empty symbols) upon anion titration in acetone, 100 mM $TBAClO_4$. Lines represent fits according to 1:1 host-guest stoichiometric binding model.[2a]

We further conducted voltammetric sensing studies in the much more polar, aqueous solvent system ACN/1% H_2O (+100 µM $HClO_4$). These electrolyte conditions were, as discussed in more detail in the main text, chosen to further enhance the chemical stability of the ferrocenium redox-state and to enable spectroelectrochemical experiments under bulk electrolytic conditions (Figure 6). Even in this competitive solvent system both receptors displayed smaller, but still significant cathodic voltammetric shifts of the Fc/Fc$^+$ redox couple upon anion addition (Figure S38). Interestingly, **BDP-Fc•XB** now displayed a preference for Br^- with ΔE_{max} = –26 mV and no response towards bisulfate. In contrast, **BDP-Fc•HB** maintained a preference towards HSO_4^- with ΔE_{max} = –37 mV and unexpectedly also showed a greater cathodic shift than its XB congener towards Cl^- (–30 mV vs. –21 mV), a rare reversal in sensitivity.[16]

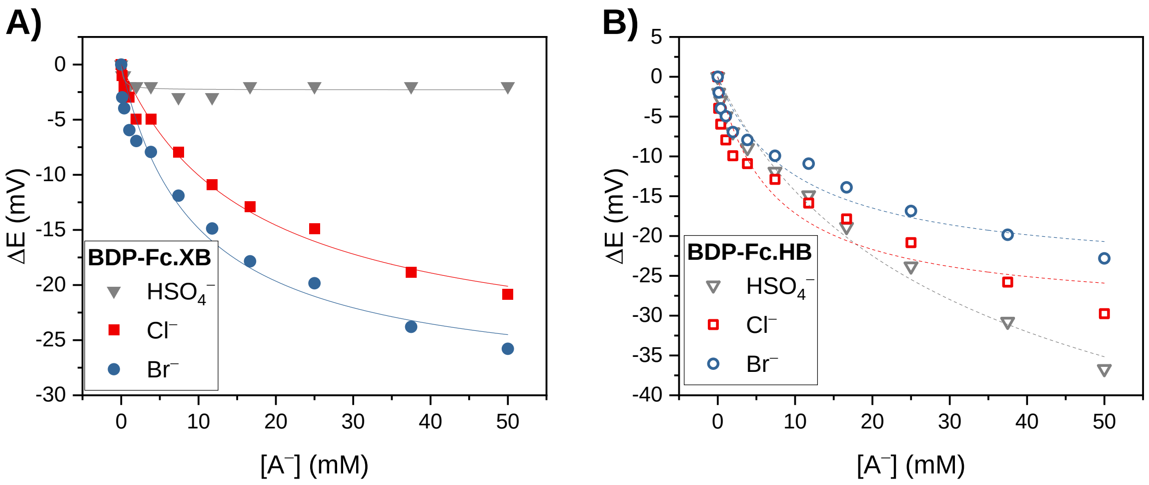

Figure S38. Cathodic voltammetric shifts of 0.25 mM a) **BDP-Fc•XB** and b) **BDP-Fc•HB** in ACN/1% H_2O, 100 mM $TBAClO_4$, 100 µM $HClO_4$ upon titration with various anions (A^- = HSO_4^-, Cl^- and Br^-). The overall ionic strength was kept constant at 100 mM throughout. Lines represent fits according to 1:1 host-guest stoichiometric binding model.

Table S1. Maximum cathodic voltammetric shifts ΔE_{Max} (mV)[a] of **BDP-Fc•XB/HB** in acetone or ACN/1% H_2O in the presence of various anions.

	**Acetone**		**ACN/1% H_2O**	
	**BDP-Fc•XB**	**BDP-Fc•HB**	**BDP-Fc•XB**	**BDP-Fc•HB**
**Cl^-**	−64	−61	−21	−30
**Br^-**	−28	−28	−26	−23
**HSO_4^-**	−53	−73	−2	−37

a – in the presence of 50 mM anion, apart from Br^- for BDP-Fc•XB (at 4 mM). Estimated error ± 2 mV.

9. Bisulfate binding studies with neutral HB receptors

To investigate the bisulfate binding preferences of the neutral receptors, fluorescence binding studies of **BDP-Ph•HB** with HSO_4^- were carried out in acetone. As shown in Figure S39, significantly smaller fluorescence enhancements were observed than for chloride and bromide. Fitting of the binding isotherm revealed weak binding with K = 43 ± 1 M^{-1}.

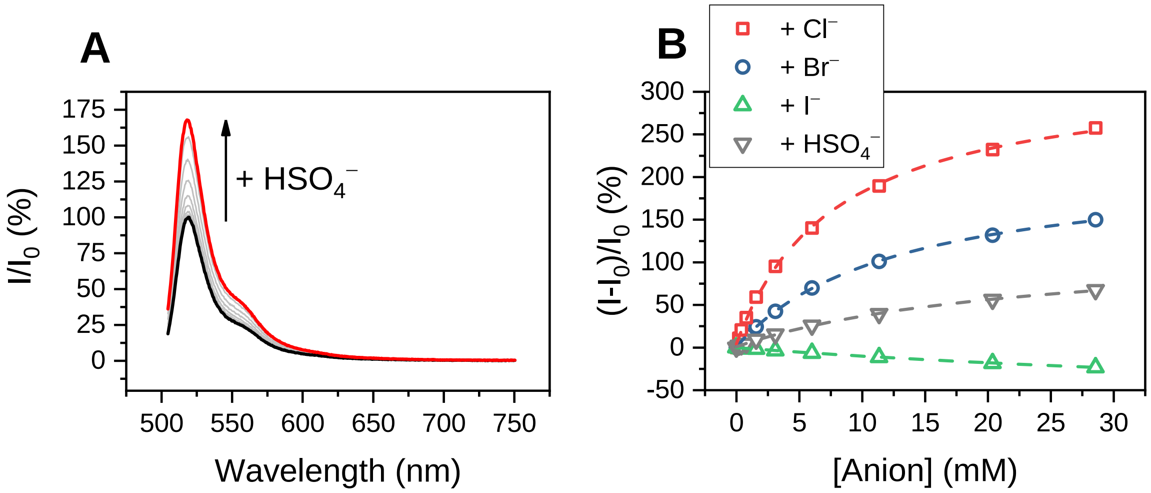

Figure S39. A) Normalised fluorescence emission response of 1 µM **BDP-Ph•HB** upon addition of increasing concentrations of HSO_4^- (up to ≈28 mM) in acetone. B) Relative fluorescence emission response of 1 µM **BDP-Ph•HB** upon addition of various anions in acetone. Dotted lines represent fits according to 1:1 host-guest stoichiometric binding model.

In the more competitive spectroelectrochemical solvent system (acetonitrile containing 100 mM $TBAClO_4$ and 200 µM $HClO_4$) bisulfate binding is thus expected to be negligible. This was confirmed by addition of up to ≈28 mM HSO_4^- to **BDP-Ph•HB** which, as shown in Figure S40, induced only minimal changes in the emission spectrum, which were too small for quantitatively analysis. We therefore conclude that HSO_4^- is not appreciably bound by **BDP-Ph•HB** in this solvent system, and therefore, due to their similar binding affinities, HSO_4^- is also not appreciably bound by **BDP-Fc•HB** in its neutral, reduced state under the conditions of the spectro-electrochemical experiments, which is also in agreement with the negligible changes in emission of the neutral **BDP-Fc•HB** in the same spectroelectrochemical solvent system (Figure S41). Further evidence for negligible binding to the neutral state of the receptor was also obtained by fitting of the voltammetric response isotherms in ACN/H_2O 99:1 to a Nernstian 1:1 host-guest stoichiometric model (see Figure S38B),[2] revealing negligible binding in the reduced receptor state with $K_{Neutral}$ = 7 ± 5 M^{-1}, but significant binding in the oxidised receptor state with K_{Ox} = 89 ± 15 M^{-1}.

This highlights the importance of the redox-modulation of binding and the in-situ generation of a charged, cationic receptor state for the recognition of HSO_4^- by **BDP-Fc•HB** during the spectro-electrochemical experiments as discussed in the main text.

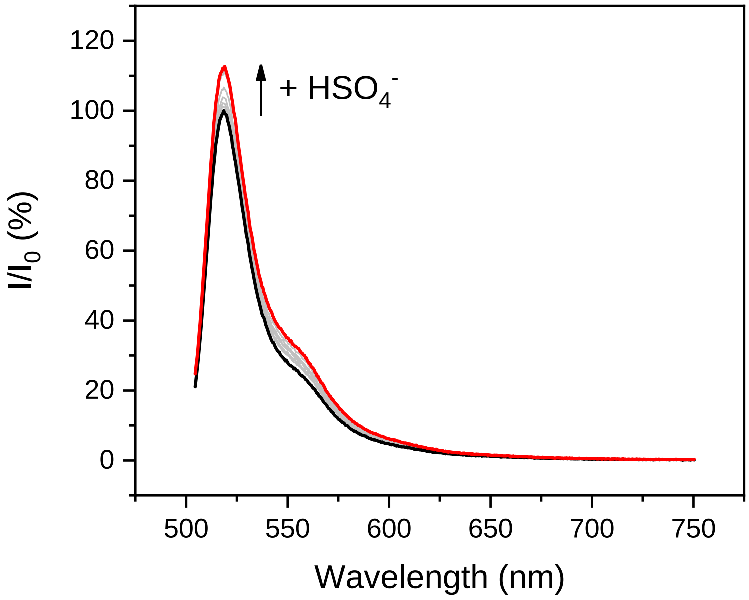

Figure S40. Fluorescence emission response of 1 µM **BDP-Ph•HB** in ACN, 100 mM $TBAClO_4$ and 200 µM $HClO_4$ upon addition of increasing concentrations of HSO_4^- (up to ≈28 mM).

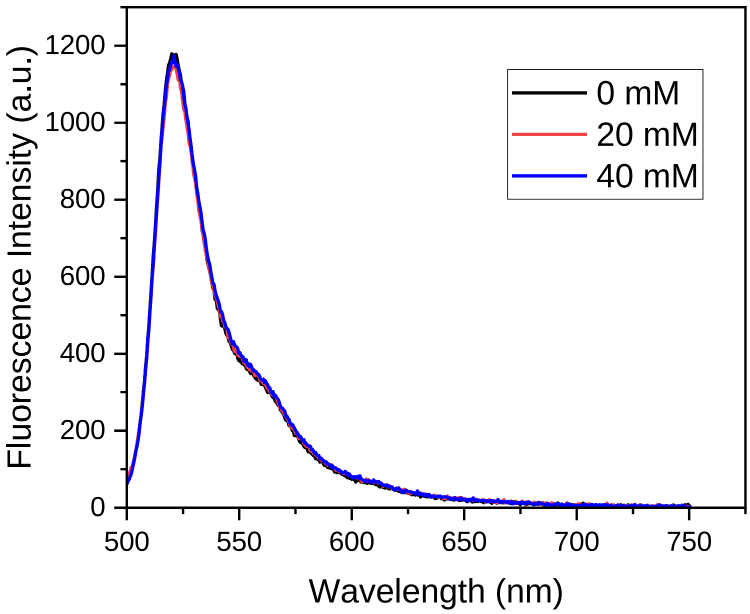

Figure S41. Emission spectra of 500 nM **BDP-Fc•HB** in ACN, 100 mM $TBAClO_4$ and 200 µM $HClO_4$ in the presence of increasing concentrations of $TBAHSO_4$ (see also Figure 7A in the main text).

References

† Iodide was not tested as an anion in these voltammetric titrations as its inherent redox activity overlaps with that of the ferrocene reporter group. We instead included HSO_4^- as an additional analyte.

[1] C. Würth, M. Grabolle, J. Pauli, M. Spieles, U. Resch-Genger, *Nat. Protoc.* **2013**, *8*, 1535-1550.

[2] a) R. Hein, X. Li, P. D. Beer, J. J. Davis, *Chem. Sci.* **2021**, *12*, 2433-2440; b) S. C. Patrick, R. Hein, M. Sharafeldin, X. Li, P. D. Beer, J. J. Davis, *Chem. Eur. J.* **2021**, *27*, 17700-17706.

[3] M. Albrecht, A. Lippach, M. P. Exner, J. Jerbi, M. Springborg, N. Budisa, G. Wenz, *Org. Biomol. Chem.* **2015**, *13*, 6728-6736.

[4] H. Gallardo, A. J. Bortoluzzi, D. M. P. De Oliveira Santos, *Liquid Crystals* **2008**, *35*, 719-725.

[5] K. M. Bąk, S. C. Patrick, X. Li, P. D. Beer, J. J. Davis, *Angew. Chem. Int. Ed.* **2023**, *62*, e202300867.

[6] A. S. K. Hashmi, R. Döpp, C. Lothschütz, M. Rudolph, D. Riedel, F. Rominger, *Adv. Synth. Catal.* **2010**, *352*, 1307-1314.

[7] Y. V. Zatsikha, N. O. Didukh, R. K. Swedin, V. P. Yakubovskyi, T. S. Blesener, A. T. Healy, D. E. Herbert, D. A. Blank, V. N. Nemykin, Y. P. Kovtun, *Org. Lett.* **2019**, *21*, 5713-5718.

[8] S. Toliautas, J. Dodonova, A. Žvirblis, I. Čiplys, A. Polita, A. Devižis, S. Tumkevičius, J. Šulskus, A. Vyšniauskas, *Chem. Eur. J.* **2019**, *25*, 10342-10349.

[9] J. Ding, K. Feng, C.-H. Tung, L.-Z. Wu, *J. Phys. Chem. C* **2011**, *115*, 833-839.

[10] X. Wu, W. Wu, X. Cui, J. Zhao, M. Wu, *J. Mater. Chem. C* **2016**, *4*, 2843-2853.

[11] a) X. Wu, W. Wu, X. Cui, J. Zhao, M. Wu, *J. Mater. Chem. C* **2016**, *4*, 2843-2853; b) Y. V. Zatsikha, T. S. Blesener, A. J. King, A. T. Healy, P. C. Goff, N. O. Didukh, D. A. Blank, Y. P. Kovtun, V. N. Nemykin, *J. Phys. Chem. B* **2020**, *125*, 360-371.

[12] a) K. Vandewal, J. Benduhn, V. C. Nikolis, *Sustain. Energy Fuels.* **2018**, *2*, 538-544; b) R. Marcus, *J. Phys. Chem* **1989**, *93*, 3078-3086.

[13] a) R. Hein, P. D. Beer, J. J. Davis, *Chem. Rev.* **2020**, *120*, 1888-1935; b) S. C. Patrick, R. Hein, A. Docker, P. D. Beer, J. J. Davis, *Chem. Eur. J.* **2021**, *27*, 10201-10209.

[14] a) R. Oliveira, S. Groni, C. Fave, M. Branca, F. Mavre, D. Lorcy, M. Fourmigue, B. Schollhorn, *Phys. Chem. Chem. Phys.* **2016**, *18*, 15867-15873; b) C. Fave, B. Schöllhorn, *Curr. Opin. Electrochem.* **2019**, *15*, 89-96.

[15] a) J. Y. C. Lim, P. D. Beer, *Chem* **2018**, *4*, 731-783; b) J. Pancholi, P. D. Beer, *Coord. Chem. Rev.* **2020**, *416*, 213281.

[16] R. Hein, P. D. Beer, *Chem. Sci.* **2022**, *13*, 7098-7125.
